# Divinylazobenzene-Q-Cage Silyloxy-Silsesquioxane-Based Reversible Photoactuatable Network Polymer Sponges

**DOI:** 10.1021/acs.macromol.5c03635

**Published:** 2026-07-21

**Authors:** Cory B. Sims, Ethan T. Chandler, Herenia Espitia Armenta, Mehdi Erfani Jazi, Andrew C. Deller, Joseph C. Furgal

**Affiliations:** Department of Chemistry and Center for Photochemical Sciences, Bowling Green State University, Bowling Green, Ohio 43403, United States

## Abstract

The development, analysis, characterization, and solvent preference of photoresponsive hybrid 4,4’-divinylazobenzene and octa­(dimethylsiloxy)­silsesquioxane (Q_8_M_8_^H^) networks were investigated. The dynamic gel systems are formed through hydrosilylation chemistry and respond to visible and UV light, expanding and contracting to give them sponge-like properties. Their solvent preference and loading capabilities are analyzed and compared to an analogous system, and general characteristics are illustrated through UV–vis cycling, FTIR, TGA, DMA, and SEM imaging. Analysis using advanced spectroscopic methods, such as SAXS and USANS, to further understand the structural and polarity changes in these materials upon light irradiation has been unsuccessful due to the stochastic linking and reactivity of these systems. We find that using a shorter, more rigid azobenzene yields a photoresponsive sponge comparable to that of 4,4’-diallyloxyazobenzene, with a higher initial shrinkage response. SAXS spectroscopic analysis shows little overall change in structural size between static and UV-irradiated samples in their toluene-swollen states, but there are distinct changes in overall structural organization, as evidenced by the scattering profiles and, in limited cases, decreases in the water contact angles on dried gel surfaces, which are also consistent with swelling and UV–vis studies.

## Graphical abstract

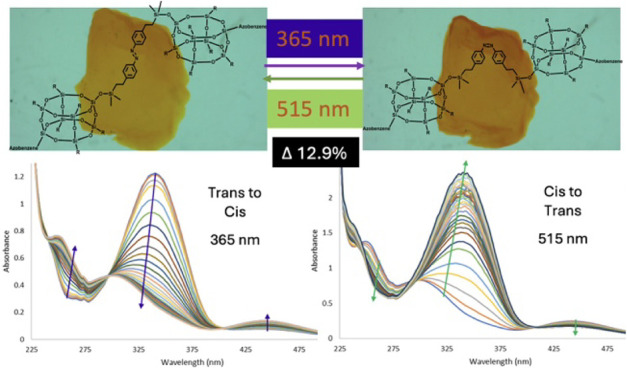

## Introduction

The ability to instill responsiveness to mechanical, chemical, thermal, or light stimuli is often desired in polymers. These polymer materials have been found to have a wide range of applications, such as photoresponsive thin films^1–3^ and gels,^4–9^ photoswitchable adhesives,^10^ pollutant absorbents,^7–9^ and photohealable networks.^11,12^ Photoswitching materials use distinct wavelengths of light (UV/vis) to isomerize, and there are various types of these monomers, including spiropyrans,^13–15^ diarlyethenes,^16^ and azobenzenes.^17–19^ Among these, azobenzenes are the only group that undergoes a structural change without complete bond cleavage, making them less susceptible to adverse reactivity. These molecules absorb UV light, exciting the N=N double bond and causing a reversible isomerization from the trans to the cis isomer.^19–21^ Absorbance of visible light, when combined with the strain of the cis isomer, allows the reverse conformational change back to the thermostable trans form. The activation wavelengths of cross-linkers have been modified by substituting different positions on the benzene rings of the system. This is primarily done to enable a red-shift of azobenzene’s absorption, making it possible for trans→cis conversion under visible light for use in biological systems, but often at the expense of cis→trans efficiency.^21–24^ Adjustments of the general azobenzene structure have also enabled the addition of reactive sites to integrate these photoswitches into polymers.^25–28^ The photoinduced actuation of these molecules results in a 3.5 Å change in distance from the 4 and 4’ para carbons (Figure 1), which is amplified by each instance of the group within a material (i.e., as a cross-linker), causing an alteration in the overall material and providing a system-wide photoresponse.^1,29^ An extensive range of polymeric functionalities has been imparted on azobenzene to incorporate them into materials, including poly­(ether sulfones), polyaniline,^2^ polyacrylates,^8,9^ polyureas,^3^ alkenes,^30,31^ and siloxanes.^11^ When photoswitches are added to form 3D structures, they can be used as effective, reusable absorbents, like sponges. Previous studies have used acrylates as polymer bases to capture oils,^22,23^ while hydrogels have been used to create sponge-like materials that can actuate by up to 20% in aqueous systems.^29,31^ However, these materials are less selective for pollutants. To improve these materials, they must be compatible with nonaqueous liquids while retaining photofunctionality.

**Figure 1:**
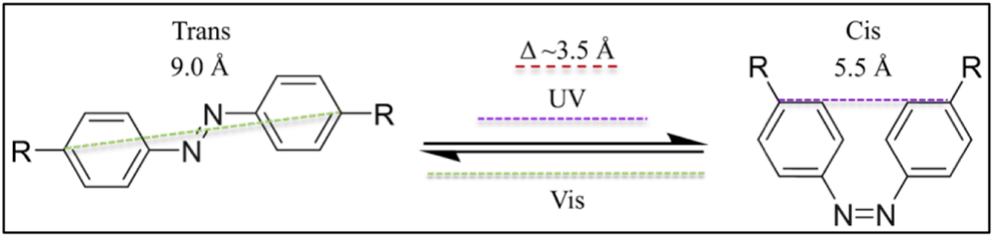
Change in distance between the 4 and 4’ para carbons of azobenzenes is caused by the reversible, light-induced isomerization. An approximate change in distance of 3.5 Å was reported by Kumar and Neckers.^29^

An instrumental group of polymeric building blocks is silsesquioxanes. These materials are composed of the formula (RSiO_3/2_)_n_, which yields 3D “cage” structures that are readily integrated into polymers via their organic R-group-terminated corners.^32–38^ One distinct derivative, often referred to as silsesquioxanes, are Q-type silica cages, which have a slightly altered structure where an oxygen is directly attached on each corner (ROSiO_3/2_)_n_ and provide more flexibility/rotation of the cage corner groups.^39,40^ One such system is octakis-dimethylsiloxy-silica (referenced herein as silsesquioxane, Q_8_M_8_^H^, Q_8_), which has corner terminations of −O–Si­(CH_3_)_2_H allowing hydrosilylation reactivity with alkene groups.^41–44^ Prior research has shown successful integration of these Q_8_M_8_^H^ cages with azobenzenes to develop composites,^26,45^ films,^46^ and gels^5,6,47^ with photoresponsive behaviors. Networks containing 4,4’-diallyloxy-azobene and Q_8_ resulted in sponges exhibiting an ∼18% reversible shrinkage-expansion change in nonaqueous solutions after irradiation, and are believed to be due to the near-zero-energy rotation of the dimethylsiloxy group on the cage corners, which reduces the barrier to isomerization.^5^ These results were comparable to azobenzene-hydrogel materials but show an improved route to pollutant remediation due to their hydrophobicities.

Herein is a study of the Q_8_ −4,4’-divinyl-azobenzene (DVA) gel development and comparison to the Q_8_ 4,4’-diallyloxy-azobenzene (DAA) system.^5^ The vinyl system was chosen to compare the influence a shorter linker would have over the shrink and expand properties. The new materials are formed using hydrosilylation chemistry with Karstedt’s catalyst, creating a bond between the vinyl and silane (Figure 2).^5^ Effects of different solvents on the swelling and actuation of the materials by 365 nm (contract) and 515 nm (expand) irradiation will provide insights for their potential use as pollutant “sponges” which can be “wrung out” without physical interactions (Figure 3). Preferences for absorbing solvents over water will identify which materials are ideal for selectively removing pollutants from aqueous environments. Synthesis and characterization of the DVA system by UV–vis, FTIR, TGA, and SEM provides further insights into the material’s functionality. The enclosed work focuses on a 1:2 mol ratio of Q_8_:DVA materials due to the superior performance of this ratio reported by Hu and Furgal^5^ Despite this, several select experiments were conducted on the swelling efficiency of the 1:3 DVA (Q_8_:Azo) gels to investigate if changing the Q_8_:DVA photoswitch linking density affects the “sponge” efficiency observed. The effects on their solvent uptake, load, and preference for various environmental pollutants are detailed below, and general characteristics are illustrated using UV–vis, FTIR, TGA, and SEM imaging. Further advanced characterization to quantify structural/polarity features was undertaken, including Ultrasmall-Angle Neutron Scattering (USANS),^48–50^ small-angle laser light scattering (SALS),^51^ Small Angle X-ray Scattering (SAXS),^52^ dye sensitivity studies,^53^ and water contact angle measurements of dry surfaces.

**Figure 2:**
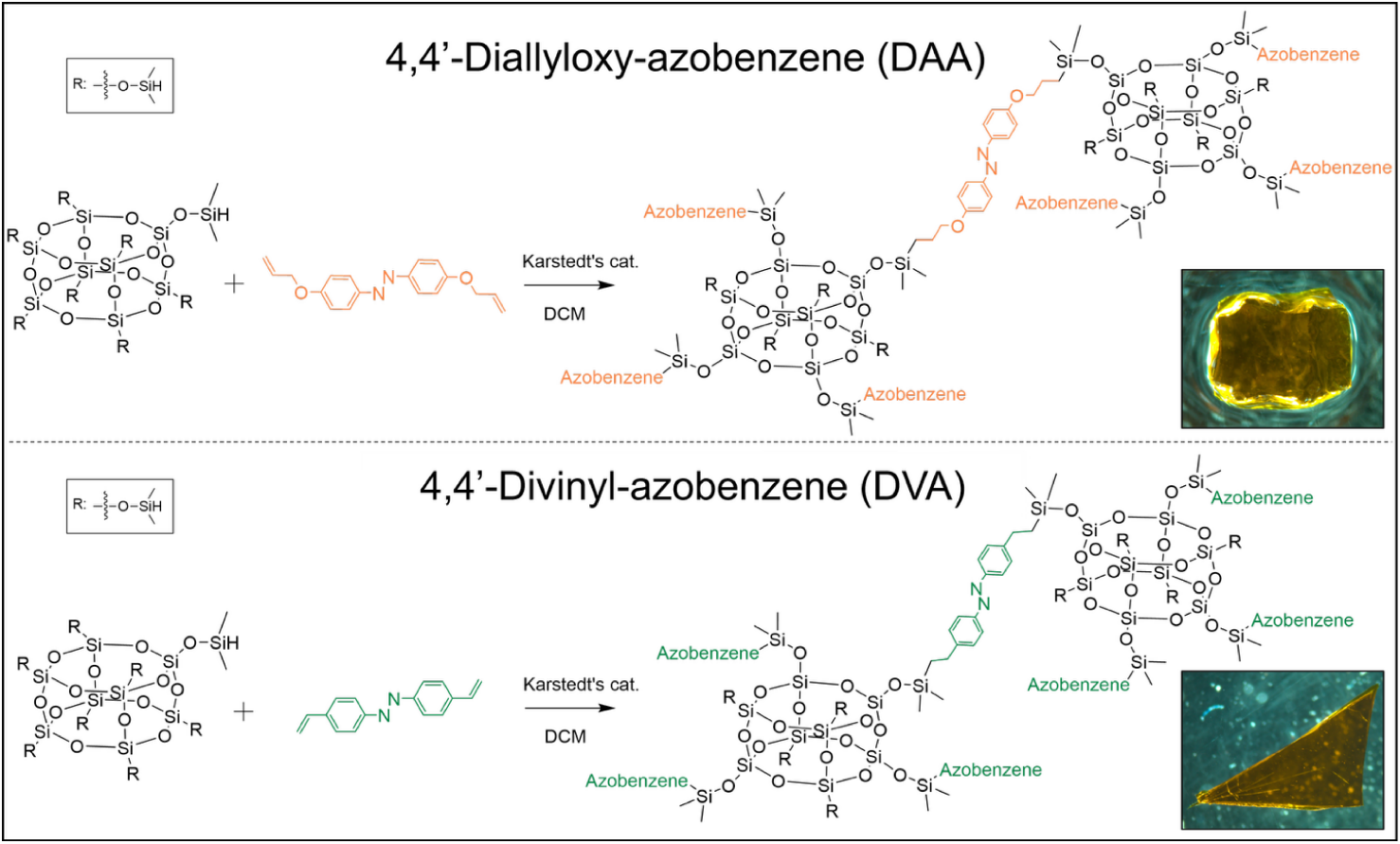
Polymerization reaction of octa­(dimethylsiloxy)­silsesquioxane (Q_8_M_8_^H^) with 4,4’-diallyloxyazobenzene (DAA) and (DVA) with Karstedt’s catalyst in DCM to form photoresponsive gels. This depicts the bonding that occurs in every azobenzene derivative molecule and shows the differences between the monomers.

**Figure 3:**
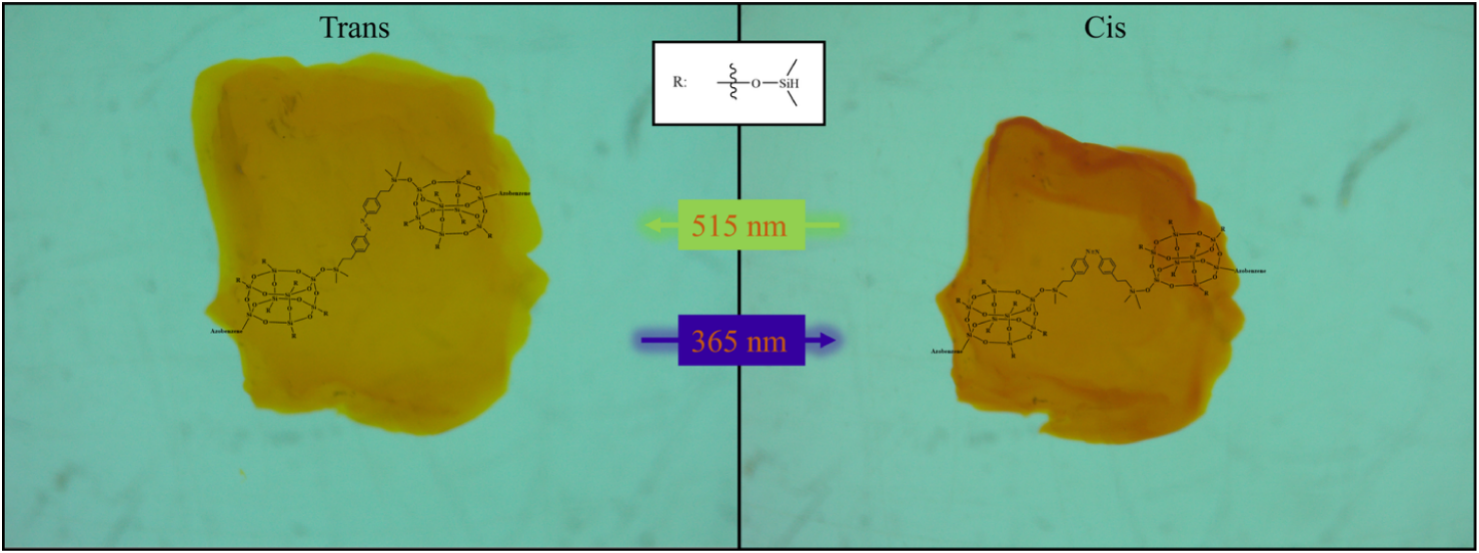
An example of morphological changes of the Q-cage/4,4’-divinyl-azobenzene gel when exposed to UV (365 nm) and green visible (515 nm) light, causing transformations between the trans and cis forms.

## Experimental Methods

### Materials

All materials and items used in this project were obtained from trusted vendors or donated and used as received without any alterations or modifications. Dichloromethane (DCM) and acetone were procured from EMD Chemicals, while tetra-*n*-butylammonium fluoride (1 M in THF), chloroform-d (NMR, 99.8+, 0.3 v/v % TMS), and acetonitrile (LCMS/MS GR) were obtained from Acros Organics. Palladium­(II) bromide (39.8–40.1% Pd) was sourced from AK Scientific, and JohnPhos (2-(di*tert*-butylphosphino)­biphenyl, 97%) was bought from AmBeed. Toluene and THF (tetrahydrofuran, HPLC GR) were purchased from Supelco, while D_4_V (2,4,6,8-tetramethyl-2,4,6,8-tetravinylcyclotetrasiloxane, > 97%) and hexafluorobenzene (99.0%) were obtained from TCI America. Reichardt’s dye was purchased from Signma Aldrich Inc. Sigma-Aldrich provided anhydrous acetonitrile (99.8%), while Karstedt’s catalyst (platinum-divinyltetramethyldisiloxane complex, 2% Pt in xylene) was procured from Gelest Inc. Alfa Aesar was the source of 4-bromoaniline (98+%), manganese­(IV) oxide (90%), and 9-fluorenone (98+%). Both gasoline (regular unleaded) and diesel were obtained from Marathon, and we used reverse osmosis purified water in all our testing. Irradiation was conducted with a 365 nm lamp from ThorLabs (COP1-A Olympus—M365LP1-C1 15.0 V Lamp) and a 515 nm flashlight from Lumenshooter. Mayaterials Inc. provided us with octakis­(dimethylsiloxy)­silsesquioxane (Q_8_M_8_^H^, Q_8_), while 4,4’-diallyloxy-azobenzene was provided by Hu and Furgal.^5^

### Synthesis of the 4,4’-Divinyl-Azobenzene Monomer

#### Synthesis of 4,4’-Dibromo-Azobenzene

Syntheses of the 4,4’-dibromo-azobenzene were achieved by the oxidative cross-coupling of 4-bromoanaline by MnO.^54,55^ In a 500 mL round-bottom flask, 6.8821 g of 4-bromoaniline (40.008 mmol) was mixed with 20.8379 g of manganese­(IV) oxide (239.681 mmol) in 200 mL of toluene. This mixture was refluxed for 3 days at 135 °C. Next, the solution was filtered and rinsed with boiling toluene. The filtrate was then rotovaped until a precipitate formed, allowed to reach room temperature, and then placed in the freezer. This purification process was repeated after 24 h, and then a final filtration was performed to collect 4.9700 g of an orange crystalline product at 73% yield. Products confirmed by ^1^H NMR (Figure S1). ^1^H NMR (500 MHz, CDCl_3_, δ ppm): 7.59–7.72 (d, 4H, Ar–H), 7.88–7.91 (d, 4H, Ar–H). ASAP-Q-TOF-HRMS (Figure S2): [M+1]^+^ calcd for C_12_H_8_Br_2_N_2_ 338.9132 *m*/*z*; Found: [M+1]^+^ 338.9899 *m*/*z*.

#### Synthesis of 4,4’-Divinyl-Azobenzene

Vinylation of the 4,4’-dibromo-azobenzene is carried out by palladium-catalyzed Heck reactions, derived from methods of Denmark and Butler^56–58^ A solution was prepared by combining 11.8 mL of TBAF (1 M in THF), 1.0143 g (2.943 mmol) D_4_V (2,4,6,8-tetramethyl-2,4,6,8-tetravinylcyclotetrasiloxane), and 20 mL THF in a 100 mL round-bottom flask. The mixture was stirred at 50̊C for 30 min. A second solution containing 1.0065 g (2.960 mmol) 4,4’-dibromo-azobenzene, 79.0 mg (0.297 mmol) palladium bromide, and 189.4 mg (0.635 mmol) of John Phos (2-(di*tert*-butylphosphino)­biphenyl) was made and then slowly added to the first solution. The entire solution was stirred for 24 h at 50 °C. The crude product was purified by column chromatography on silica gel using a 1:4 DCM:hexane mixture, and the second band was collected as the pure product. The resulting precipitate was filtered by vacuum filtration to yield 0.3149 g of product, a 45% yield. Products confirmed by ^1^H NMR (Figure S3). ^1^H NMR (500 MHz, CDCl_3_, δ ppm): 5.35–5.38 (d, 2H, −CH=CH_2_), 5.84–5.90 (d, 2H, −CH=CH_2_), 6.74–6.84 (m, 2H, −CH=CH_2_), 7.54–7.91 (d, 4H, Ar–H), 7.88–7.91 (d, 4H, Ar–H). ASAP-Q-TOF-HRMS (Figure S4): [M+1]^+^ calcd for C_16_H_15_N_2_ 235.1235 *m*/*z*; Found: [M+1]^+^ 235.1246 *m*/*z*.

### Gel Preparation and Solvent Exchange

#### Preparation of the Vinyl-Octa­(dimethylsiloxy)­silsesquioxane (Q_8_M_8_^H^/Azo) Gels

The method for preparing the 1:2 and 1:3 mol ratios of Q_8_M_8_^H^/Azo gels was adapted from techniques reported by Hu and Furgal.^5^ To prepare the polymer, 0.0564 g (0.055 mmol) Q_8_M_8_^H^ and 0.0260 g (0.111 mmol – 1:2, 1 cage [8 reactive groups] to 2 azo [4 reactive groups]) or 0.0389 g (0.166 mmol – 1:3, 1 cage [8 reactive groups] to 3 azo [6 reactive groups]) of 4,4’-divinyl-azobenzene were combined in a 20 mL vial and dissolved in 2 mL DCM. In a separate vial, 1 drop of Karstedt’s catalyst (0.0007 mmol Pt) was added to 1 mL of DCM and mixed thoroughly before being added to the first solution. The mixture was then heated to 36 °C to initiate polymerization. Then, 2 mL of DCM was added to prevent evaporation. Impurities were removed through solvent exchange (see below) before the material was cut and analyzed.

#### Preparation of the Allyloxy-Octa­(dimethylsiloxy)­silsesquioxane (Q_8_M_8_^H^/Azo) Gels

The method for creating the 1:2 mol ratio Q_8_M_8_^H^/Azo gels used the techniques employed by Hu and Furgal.^5^ To prepare the polymer, 0.192 g (0.2 mmol) Q_8_M_8_^H^ and 0.13 g (0.4 mmol) 4,4’-diallyloxy-azobenzene were combined in a 20 mL vial and dissolved in 2 mL DCM. In a separate vial, 1 drop of Karstedt’s catalyst (0.0007 mmol Pt) was added to 1 mL of DCM and mixed thoroughly before being added to the first solution. The mixture was heated to 36 °C to initiate polymerization, and then 2 mL of DCM was added to prevent evaporation. Impurities were removed through solvent exchange before the material was cut and analyzed.

#### Solvent Exchange and Analysis

During the analysis, the gels underwent solvent exchange for purification and studies in other solvent systems. The existing solvent was decanted and replaced with a new solvent (10 mL, ∼90% of the total volume). This promotes the diffusion of unused reagents and encourages solvent replacement. Samples were left in ambient conditions with the new solvent for 2 h, then the procedure was repeated for 3 cycles. The process was carried out at 24 h postpolymerization and after each solvent-swelling study. Light microscopy was utilized to capture images during the actuation process. Initial photos were captured before the samples were irradiated with 3 cycles of 365 nm (17 mW/cm^2^, ThorLabs (COP1-A Olympus—M365LP1-C1 15.0 V, 24 mW/cm^2^ Lamp) and 515 nm light (15 mW/cm^2^, Lumenshooter), each for 30 continuous minutes, with new images captured between exposure wavelength changes. When a gel needed to be replaced due to degradation, a new sample was imaged in DCM as a reference before running tests, and future calculations used this as the maxima reference point.

#### Solvent Preferability Analysis

Solvent preferences were demonstrated using DCM and THF. A partially dried sample of 1:2 DVA gel was placed in a 0.014 M 9-fluorenone solution in DCM (25 mg in 10 mL) and allowed to swell. After 24 h, the sample was placed on a glass Petri dish and encapsulated in water using a Pasteur pipet. UV light was applied to the sample for ∼30 min while the process was recorded with light microscopy to observe the expulsion of the solution. To understand the selective uptake of mixed solvents, a 1:2 DVA sample was dried and placed on a Petri dish. Four drops of a 1 M 9-fluorenone mixture (1 mL water:1 mL THF) were added to the sample and then exposed to a green light (515 nm) while recording through light microscopy. Time-lapse images were obtained from each video.

#### UV–Vis Cycling and Photostationary State Analysis

56.5 mg Q_8_M_8_^H^ (0.06 mmol), 26.0 mg (0.11 mmol) 4,4’-divinylazobenzene in 1.2 mL dichloromethane were mixed in a vial. In a separate vial, 3–5 drops of the solution were mixed with 1 drop of 2% Karstedt’s catalyst in xylenes and swirled thoroughly. One to two drops of the polymerizing mixture were deposited onto a quartz plate. Another quartz plate was set on top of it and pressed gently, so that the polymerization was completed between the quartz plates, and the sample was allowed to cure for 10 min with hand palm warmth. The sample path length was measured to be ∼6 μm. The thickness of the gel was determined by digital micrometer (Mitutoyo IP65, Mitutoyo, Kawasaki, Japan) by comparing two quartz plates before and after sandwiching the gel. Once the plates could not be pulled apart easily, they were set in the Cary 60 UV–vis. Consecutive UV–vis were then taken of them while under continuous 365 and 515 nm irradiation until the spectra remained stationary, ∼30 s between capture points. The same experiment was conducted with the 1:3 Q_8_M_8_^H^:DVA mixture, except the masses were 56.4 mg Q_8_M_8_^H^ (0.06 mmol) and 32.8 mg (0.14 mmol). Absorbances at 365 and 450 nm were then plotted as a function of cycle number. The photostationary state for each gel was calculated using a combined^1^H NMR and UV–vis method.^59–61^ ^1^H NMR was used to determine the base trans/cis ratio in the 4,4’-divinylazobenzene monomer to establish a starting point for the azobenzene ratio within the material. This was used as the basis for the initial concentration ratio of trans peak in UV–vis of the material. A molar absorptivity of 8,311 M^–1^cm^–1^ was used from a model 4,4’dimethylazobenzene as determined at 365 nm.^60^ The change in absorption upon irradiation with 365 nm LED, 24 mW/cm^2^ for ∼5 min (or until the spectrum stopped changing) to reach a photostationary state (PSS). The concentrations of the trans isomer were calculated from Beer–Lambert law at the beginning, before irradiation (^1^H NMR ratio), and at the final state (PSS) at 365 nm. Combining the initial state and change enabled estimation of the ratios at the PSS. The molar absorptivity of the cis form at 365 nm was ∼1% of that of the trans form and was considered negligible for an estimation of the states.^60^

### Light Microscopy and Surface Area

Images were taken using a Zeiss Stemi 2000-C light microscope (Carl Zeiss Meditec Inc., Dublin, CA, USA) fitted with an AxioCam ERc5s camera at a magnification of 0.65x. Using Adobe Photoshop (V. 25.1.0) Magic Wand Tool, the edges of the gels and backdrop were highlighted, and the difference between the selected pixels and the entire image was calculated as the material surface area. The results were used to determine the fluctuations in surface area swelling in response to both solvents and irradiation. All images had an area of 307,200 pixels, and all percentages discussed were derived from pixel counts.

### Dry-Swelling Analysis

After allowing the gels to dry entirely for over a day, each was reswelled in DCM, and photos of the materials in both states were taken. The estimated change in surface area by % of the dried sample was evaluated using the microscope images.

### Nuclear Magnetic Resonance (NMR)

^1^H NMR spectra were collected on a Bruker Avance 500 MHz spectrometer with a cryoprobe and Topspin software. The minimal sample was dissolved in 0.5 mL of CDCl_3_ (0.3 v/v% TMS), and spectra were acquired from 16 scans over a 12 ppm range. Postrun analysis was performed with MestreNova.

### High-Resolution Mass Spectrometry (HRMS)

Mass spectrometry was performed on a Waters Xevo G3 Q-ToF (Milford, MA, USA) equipped with an Atmospheric Solids Analysis Probe (ASAP) in positive ionization mode. Solid samples (∼5 μg) were added to a glass capillary and heated to 300 °C over 5 min at a corona current of 2 μA to ionize them for analysis. A simultaneous lockmass spray of leucine enkephalin at 556.2775 *m*/*z* was used to verify mass accuracy simultaneously to sample volatilization.

### UV–Vis Spectroscopy

A series of absorbance spectra was collected using an Agilent (Santa Clara, CA, USA) Cary 60 UV/vis. The 4,4’-divinyl-azobenzene monomer was dissolved in acetonitrile (anhydrous, 99.8%) and diluted (<100 μM). A series of scans was taken from 200 to 800 nm every 7 s until actuation ceased. Microsoft Excel was used to plot an overlay of absorbance vs wavelength during the conformational change under 365 nm (17 mW/cm^2^, ThorLabs (COP1-A Olympus—M365LP1-C1 15.0 V Lamp) or 515 nm light (15 mW/cm^2^, Lumenshooter). For the gel system, a thin sample of the material was suspended in a cuvette filled with acetonitrile, and the process was repeated.

### Fourier Transform Infrared Spectroscopy–Attenuated Total Reflection (ATR-FTIR)

FTIR spectra were acquired using Thermo Scientific ATR-FTIR with their OMNIC Spectra software (Nicolet iS5 Fourier Transform Infrared Spectrometer iD7 Attenuated Total Reflection, Thermo Scientific, Waltham, MA, USA). Dried samples were used directly with ATR methods, placed on the ZnSe crystal. Each spectrum was generated from 16 scans in a 400–4000 cm^–1^ range with a 0.121 cm^–1^ data spacing. Graphing was done using Microsoft Excel (V. 2305).

### Raman Spectroscopy

An Advantage 532 Benchtop Raman Spectrometer (SciAps, Inc., Andover, MA, USA) with Nuspec Basic software, with an integration time of 1 s, was used to obtain spectra of dried 4,4’-divinyl-azobenzene monomer and each DVA gel.

### Thermogravimetric Analysis (TGA)

A Hitachi (Santa Clara, CA, USA) STA7200 Thermal Analysis System and NEXTA were used for thermogravimetric analysis. Samples were dried at 50 °C, under vacuum, for 48 h before analysis. The ceramic crucible was loaded with 5–20 mg of material. It was steadily heated at a rate of 10 °C/min, under constant airflow of 200 mL/min, from an initial temperature of 25 °C to a maximum of 1000 °C. Graphing was done using Microsoft Excel (V. 2305).

### Dynamic Mechanical Analysis (DMA)

A PerkinElmer DMA 8000 with Pyris Software (PerkinElmer, Inc. Hopkinton, MA, USA) was used to measure the mechanical characteristics of the DVA materials. Gel solutions were prepared according to the experimental protocol, with a reduced amount of DCM (1.2 mL), and cast in a two-piece 42 × 13 × 3.3 mm Teflon mold (base and rectangular side walls). Once a gel was formed, it was moistened with DCM, a razor was used to trace the gel, and then the top portion of the mold was removed, leaving a rectangle that was subjected to solvent exchange into THF for DMA analysis. Samples were loaded and clamped in a single-cantilever geometry, with the instrument inverted to submerge the material in a room-temperature THF bath during analysis to prevent drying. All measurements were taken at 20 °C, with a frequency of 1.0 Hz, a force multiplier of 1.5, static force of 2.0 N, dynamic force of 2.0 N, strain of 0.050 mm, and exposure to 4.78 mW cm^–2^ at 365 nm and 1.80 mW cm^–2^ at 515 nm irradiation. A 1:2 DVA gel (30.2 × 11.0 × 1.6 mm) was run for a total of 130 min, with 10 min of ambient analysis, followed by 20 min of UV and visible light exposure for 3 cycles. The 1:3 DVA gels were run under the same time and irradiation parameters, with dimensions of 36.8 × 11.1 × 2.7 mm.

### Scanning Electron Microscopy (SEM)

The 1:2 DVA gel was dried and swollen with an alcohol-based coating^62^ before exposure to 365 nm light. The cured sample was fractured and prepared for SEM imaging. An adhesive strip was used to adhere the materials to the sample specimen, and a Hummer VI-A Sputter Coater (gold/palladium) was used to apply a thin, conductive coating. A Hitachi S-2700 Scanning Electron Microscope with a lanthanum hexaboride source (Hitachi, Tokyo, Japan) was used to image the mounted samples at an aperture of 4, working distance of 13–14, 15 kV, and a magnification of 70×. Further SEM analysis was conducted on all nonirradiated gel samples after a solvent exchange process from DCM to DMSO, followed by freeze-drying (from LN_2_) of the samples on a high vacuum line over the course of 2 days.

### Small-Angle X-ray Scattering (SAXS)

SAXS was performed on DVA and DAA gel samples that were maximally swollen in toluene. The samples were analyzed using a Rigaku Ultima III high-resolution XRD instrument (Rigaku Americas, The Woodlands, TX 77381) in transmission SAXS configuration (University of Toledo Center for Materials and Sensor Characterization). A Cu Kα source (40 kV, 44 mA), a wavelength of 1.54059 Å, scanned over 0.109–7.989 degrees 2θ at a rate of 0.02°/100 s, and I­(p)=33,352/65 were used for analysis. Samples were placed in a plastic pouch for containment and inserted into a 1 mm path length stainless-steel holder in transmission mode for analysis over 12 h. For each sample, static and UV (365 nm, 20 mW/cm^2^) were analyzed (see Figure S20 for instrument sample configuration images and SI for acronym meanings). Scans were averaged, and the plastic pouch was subtracted from the data. All data analysis was conducted using the BioXTAS RAW 2.4.1 software package,^63,64^ including Guinier, GNOM Indirect Fourier Transform, and Porod volume models.

### Static Water Contact Angle Measurement

Contact angle measurements were performed to evaluate the surface wettability of dried gels before and after 10 min of UV irradiation. Images were taken immediately after ∼10 μL droplet was left on the surface in both instances. A Zeiss Stemi 2000-C light microscope (Carl Zeiss Meditec Inc., Dublin, CA, USA) equipped with an AxioCam ERc5s camera (Carl Zeiss Meditec Inc., Dublin, CA, USA) was used at 0.65× magnification to capture images. Images were analyzed using ImageJ software (version 1.54g) with the contact angle plug-in (manual points method), developed by the National Institutes of Health (Bethesda, MD, USA).

## Results and Discussion

Octakisdimethylsiloxy-silica (Q_8_M_8_^H^, Q-silsesquioxane) – 4,4’-divinyl-azobenzene (DVA) and 4,4’-allyloxy-azobenzene (DAA) photoresponsive gels were synthesized according to the methods described above using hydrosilylation in DCM. The photo actuation of the DVA gel efficiency in the presence of different solvents will be discussed below. The 1:2 Q_8_M_8_^H^-DVA (mol ratio) was the primary focus of this study, as prior research demonstrated that gel polymers of this ratio performed optimally in the DAA system, exhibiting the greatest actuation compared to other formulations.^5^ Additionally, the effect of DVA loading ratios was investigated using a 1:3 Q_8_-DVA gel for initial testing. In addition to ATR-FTIR, TGA, and SEM, materials were evaluated by swelling and actuation with 365 and 515 nm light in various solvents. A comparative analysis is presented to understand the structural, thermal, and geomorphic characteristics of polymerized materials with different azobenzene moieties. Comparisons are provided between the 1:2 and 1:3 DVA gels and the 1:2 DAA and are discussed herein.

### Gel Characterization

UV–vis analysis of the DVA monomer and 1:2 gel shows trans–cis conversion under 365 nm UV light and cis–trans conversion under 515 nm visible light. The DVA monomer (nongel), when dissolved in ACN, completed conversion to the cis form within 175 s of exposure to UV. In contrast, the reverse reaction was expectedly much slower, not completely converting after 350 s, suggesting that the solvent stabilizes the intermediate structure more than in the gel network (Figure 4A). When azobenzenes are bound to Q_8_ cages, the actuation becomes more efficient. This was achieved by suspending thin slices (∼1–2 mm thick) of each gel in ACN for spectral analysis. Conversion of the 1:2 DVA gel from trans to cis occurs within 595 s under UV irradiation, with the primary absorption wavelengths occurring at 260 and 335 nm. The reverse transformation takes 700 s with 515 nm light to completely return to the original state. The absorption under 515 nm is more pronounced in the bound form, with an increase in absorption occurring in the cis form between 400 and 525 at 450 nm (Figure 4B). The stronger π–π* absorbance during the cis-to-trans conversion is attributed to the materials undergoing their first photoisomerization from a thermally relaxed state, which may have slightly more cis conformation, allowing for a more complete conversion under irradiation than in the original state.^20^ When the 1:3 DVA gel was analyzed, the π–π* peaks could not be differentiated from the absorption between 325 and 365 nm, and the change of absorption under UV occurred within 231 s (Figure 4C), while the visible light change occurred in 700 s. The absorption bands of the 1:2 DVA gel are slightly shifted toward the UV range compared to the 1:2 DAA gel (Figure 4D), which has its primary absorption band at 365 nm, with a conversion from trans to cis taking 175 s and from cis to trans taking 350 s. Overall, the efficiency of transitions in the gel forms is attributed to the low rotational barrier imposed by the dimethylsiloxy units at the cage corners, which allows switching, as well as to the rigid spatial network of the cage, which reduces excited-state energy transfer to the solvent.

**Figure 4:**
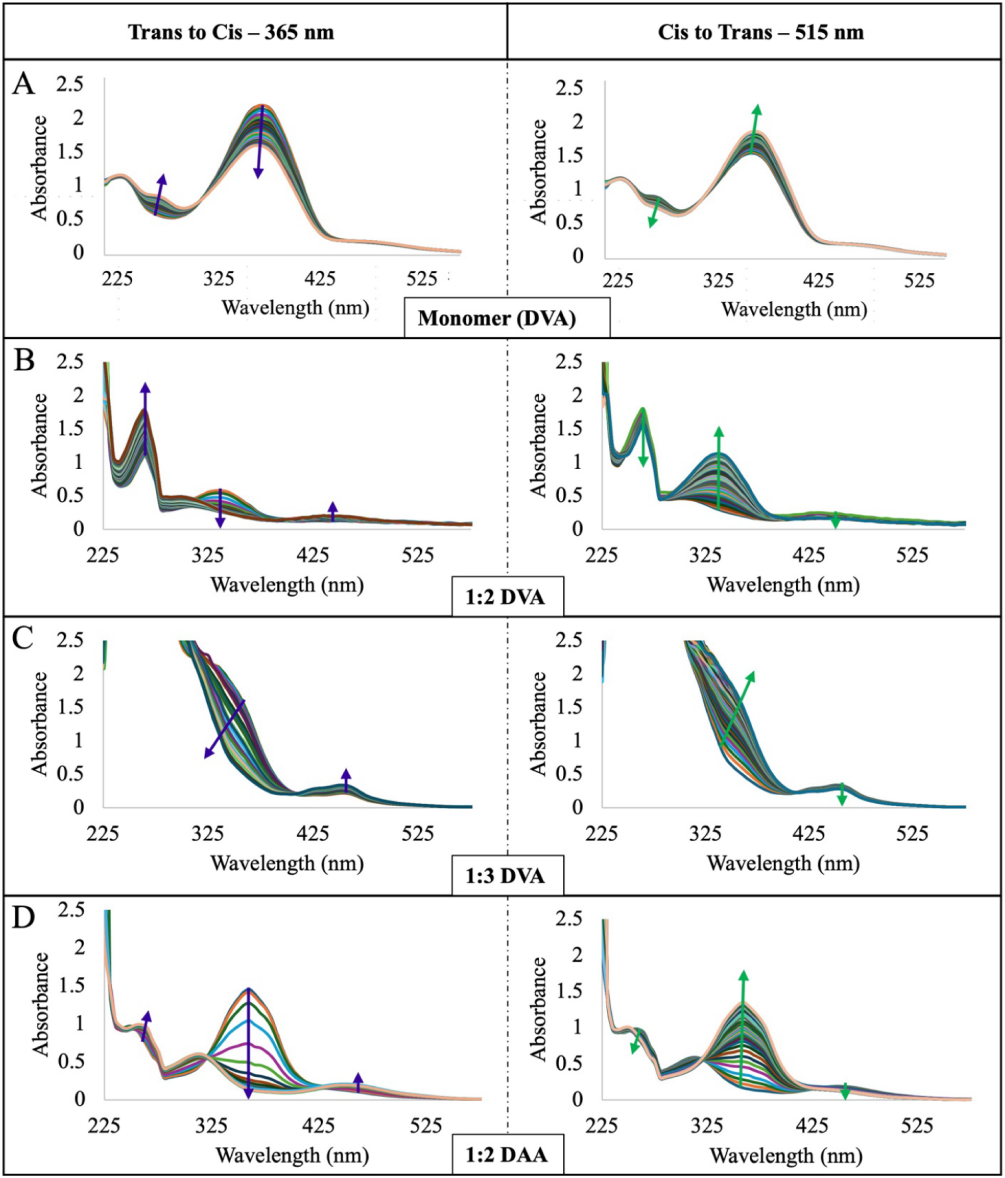
A: UV–vis spectra of the 4,4’-divinylazobenzene monomer and the Q_8_M_8_^H^ – Azobenzene gels while under light exposure and transitioning (UV – 175 s; Vis – 350 s), B: 1:2 DVA (UV – 595 s; Vis – 700 s), C: 1:3 DVA (UV – 231 s; Vis – 700 s), C: 1:2 DAA (UV – 175 s; Vis – 350 s). Each spectral line was taken at 5-s intervals, with a 2-s delay between readings, for a total interval of 7 s.

In addition to a baseline study to investigate UV–vis performance over a single cycle. Multiple-cycle analysis using DCM as the solvent was performed to assess repeated performance of the 1:2 Q_8_:DVA and 1:3 Q_8_:DVA gels (Figure 5A and B, respectively, Figures S5–S8, Tables S1 and S2), which had not previously been evaluated for UV–vis isomerization cycling.^5^ The absorption at 365 nm (max for the lamp) was used to follow these materials within the π–π* band. We find that, after an initial hysteresis likely due to unrelaxed networks in the materials after synthesis, the cycles are relatively consistent for the 1:2 derivative through 12 iterations. For the 1:3, however, after 3 cycles, we start to observe convergence in performance, with each additional cycle showing smaller changes. This suggests that the network is likely becoming irreversibly locked in a state that no longer allows efficient light-induced switching, with the samples noticeably darker in orange color than the starting state. The changes in state were also followed by the 450 nm n−π* transition (Figures S7–S8) and achieved similar findings.

**Figure 5:**
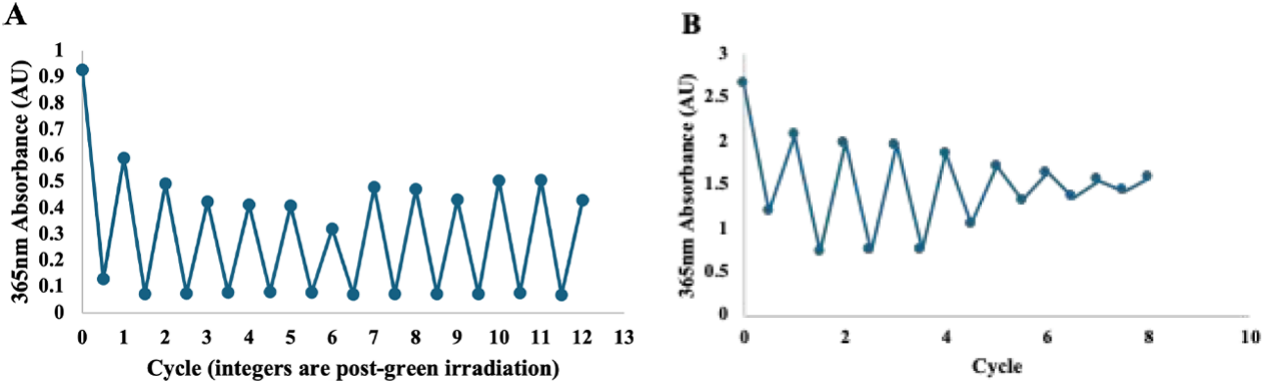
A. 1:2 Q_8_:DVA gel, and B. 1:3 Q_8_:DVA gel, UV–vis cycling study with 365 and 515 nm over 8+ cycles.

In addition to cycling the network gels, the photostationary states (PSS) were estimated for the 1:2 and 1:3 gels.^59,60^ The procedure is outlined in the Experimental Methods section. The concentrations of the separate isomers in these solutions were calculated from ^1^H NMR integration (Figure S9); the 4,4’-divinylazobenzene used for polymerization contained approximately 92% Trans and 8% Cis-isomers. This gave us a starting ratio for the study, assuming that the synthesis does not alter the isomer mixture. A molar absorptivity literature value for 4,4’-dimethylazobenzene of ε­(365 nm) = 8311 M^–1^ cm^–1^ for the π–π* transition was used to estimate the relative concentrations of the Trans and Cis isomers in the gel, accounting for a found gel thickness of ∼6 μm.^60^ For the Q_8_:DVA materials, the initial absorbance at 365 nm plus the NMR integration was used to establish the initial concentration of the trans and cis isomers. For the study, the first round of UV–vis cycling was used, because the after-synthesis “dark” ratio should most resemble this state. The samples were then irradiated until spectral changes no longer occurred, and the final Trans:Cis ratios were calculated.

In the 1:2 material, the initial concentration of Trans-DVA was calculated to be ∼0.19 M and the Cis-DVA concentration 0.017 M (Trans:Cis 92:8). The final concentrations of these isomers was calculated to be 0.027 and 0.18 M for the Trans and Cis isomers, based on their absorbances at the PSS and gave a ratio of ∼13:87, consistent with many azobenzene systems.^65^ Such a significant change in the isomer ratios from maximum to minimum, as well as strong cycling persistence, is evidence for relatively unrestricted isomerization of the azobenzenes within the polymer network, corroborating findings from our earlier papers, where the dimethylsilyl group enables free rotation, unless the network is too cross-linked.^5,66^ For the 1:3 material, the initial concentration of Trans-DVA was calculated to be 0.56 M and Cis-DVA 0.049 M. The final concentrations were calculated to be 0.25 and 0.36 M for the Trans and Cis isomers, respectively, with a Trans:Cis ratio of 41:59, suggesting that the overall efficiency of the isomerization, as expected, is lower than that for the 1:2 due to more bridging groups.

FTIR analysis was carried out on gels in the dry state. For comparison, dried-out samples of all gels were analyzed by ATR-FTIR (Figure S10). The Si–O–Si (1130–1000 cm^–1^), Si–H (2280–2080, 950–800 cm^–1^), and Si–CH_3_ (∼2960, ∼2870, ∼1380, and 1275–1245 cm^–1^) peaks from the Q_8_ were present in each material. Note that in these materials, we did not end-cap the remaining Si–H groups, so they are expected to be present. Beyond the presence of aromatic carbons from the azobenzene rings, the absence of other unsaturated carbon peaks supports polymerization of both the vinyl and allyloxy groups. The FTIR results demonstrate that both ends of the azobenzenes react, leaving no partially bridged sites.

Raman spectroscopy corroborates the FTIR analysis (Figure S11). When the 4,4’-divinyl-azobenzene monomer is compared to both the 1:2 and 1:3 gels, several changes can be observed. In the monomer, the vinyl C=C is clearly observed at 1650 cm^–1^, but is absent in both polymerized materials. All three materials still indicate the presence of the trans azo N=N from 1440 to 1450 cm^–1^, but the cis azo at 1570–1590 cm^–1^ makes up about 3–5% of the total in the thermally relaxed state, regardless of monomer or gel.

Thermal gravimetric analysis (TGA) was performed on both 1:2 gel systems and the 1:3 DVA material. A 100 °C difference in the decomposition temperatures at 5% weight loss (T_d5%_) indicates greater thermal stability in the 1:2 vinyl system than in DAA (Figure 6). However, a small amount of oxidation is observed by the weight increase at 375 °C. The 1:3 gel, while less stable than the 1:2 system, still exhibits a higher T_d5%_ than the DAA material. Still, the reduction in its stability is attributed to a lower cross-linker ratio (Q_8_). The observed ceramic yields (CY) are as follows, with the 1:2 DVA suggesting a ratio of 1:1.9, 1:3 is 1:2.9, and 1:2 DAA is 1:2.1; however, we have also found variations may occur due to the dimethylsilane groups becoming oxidized during degradation (higher CY) or not turning to SiO_2_ (burning off, lower CY). Overall, all systems exhibit high thermal stability and the expected functionalization ratios, suggesting that DVA gels are more suitable for applications at higher temperatures.

**Figure 6:**
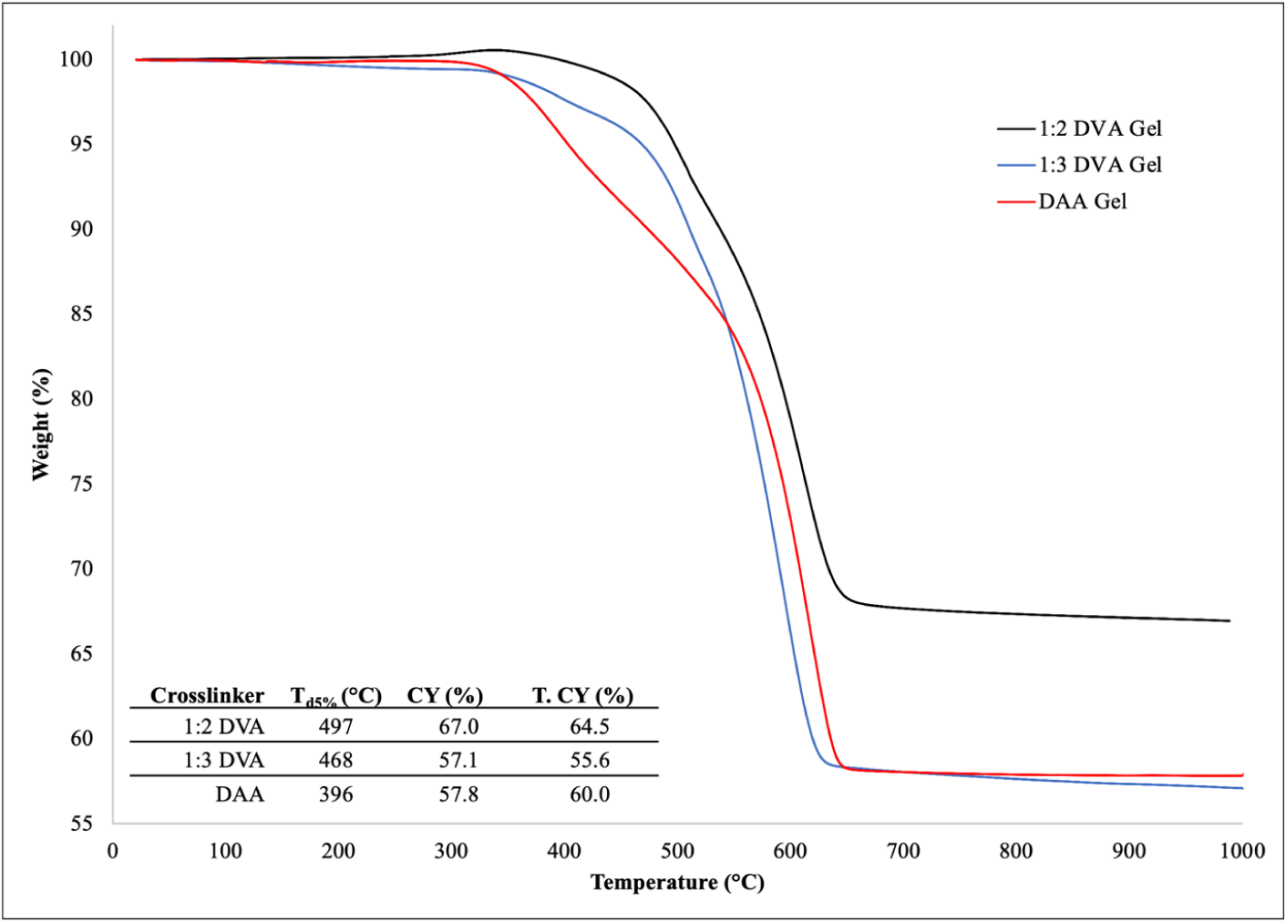
Thermal gravimetric analysis (TGA) of the three gels is used to compare their thermal stability. The T_d5%_, ceramic yield (CY), and theoretical ceramic yields (T. CY) are given for each material.

SEM analysis of air-dried 1:2 and 1:3 DVA gels (Figure S12) did not show discernible macropores despite surface roughness, in contrast to the macropores observed previously for the 1:2 DAA.^5^ When freeze-drying from high vacuum/LN_2_/DMSO instead of air-drying, some pockets/pores (>1 μm) do become evident in the fracture regions of gels (Figure S13), however we are uncertain as to whether these are “real” or an artifact of the high vacuum process. Regardless, we suspect that the interstitial space of each gel expands reversibly to accommodate certain fluid substances, forming a gel-like pore structure, as detailed below. SEM analysis of swollen gels was not performed due to instrument limitations, but the SAXS analysis below can provide some insight into the network structures.

### Gel Swelling Characteristics

The 1:2 DAA, 1:2 DVA, and 1:3 DVA gels were dried and reswollen in DCM (Figure 7). A comparison of the dry and swollen surface areas assessed the materials’ X-Y axis expansion, and a percentage of swelling was calculated. 1:2 DAA showed a surface area change of 413%. The swelling efficiencies of each material were calculated relative to itself and calibrated against the DAA material for comparison, as this material exhibited the most efficient change in size (Table 1). A visual comparison of the swelling efficiencies of each material is shown in Figure 8, with 1:2 DVA showing a 214% surface area change and 1:3 offering a 330% surface area change, which may be counterintuitive but could result from higher interactions between organic components in the material.

**Figure 7:**
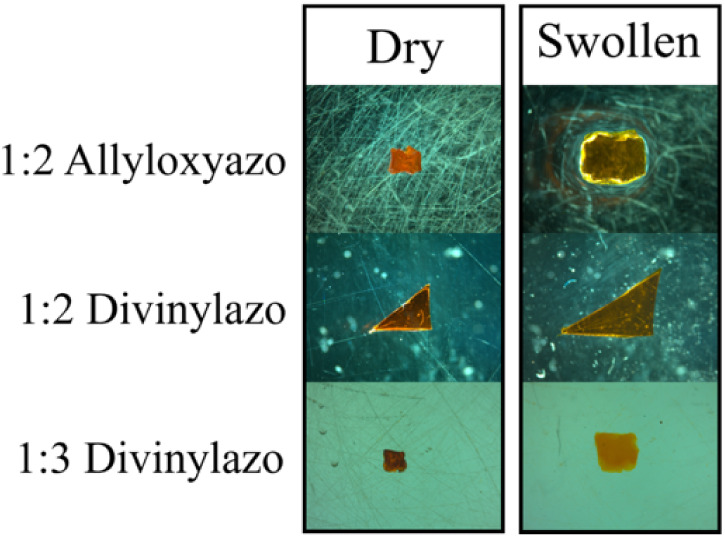
Images of the dry (left) and swollen (right) DAA and DVA gels. These images were used to analyze the change in surface area and volume, where applicable. The 1:2 allyloxyazobenzene images were also used in a previous ACS publication.^66^

**Table 1:** Change in Gel Surface Area with Drying Vs. Swelling in Dcm^a^

Swollen-Dry Gel	Change in Area (%)	Compared to Allyloxy (%)
1:2 Allyloxy	413	100
1:2 Vinyl	214	51
1:3 Vinyl	330	79

a These values were used to compare the DVA to the DAA system. The location was found by dividing the swollen sample by the dried gel surface area.

**Figure 8:**
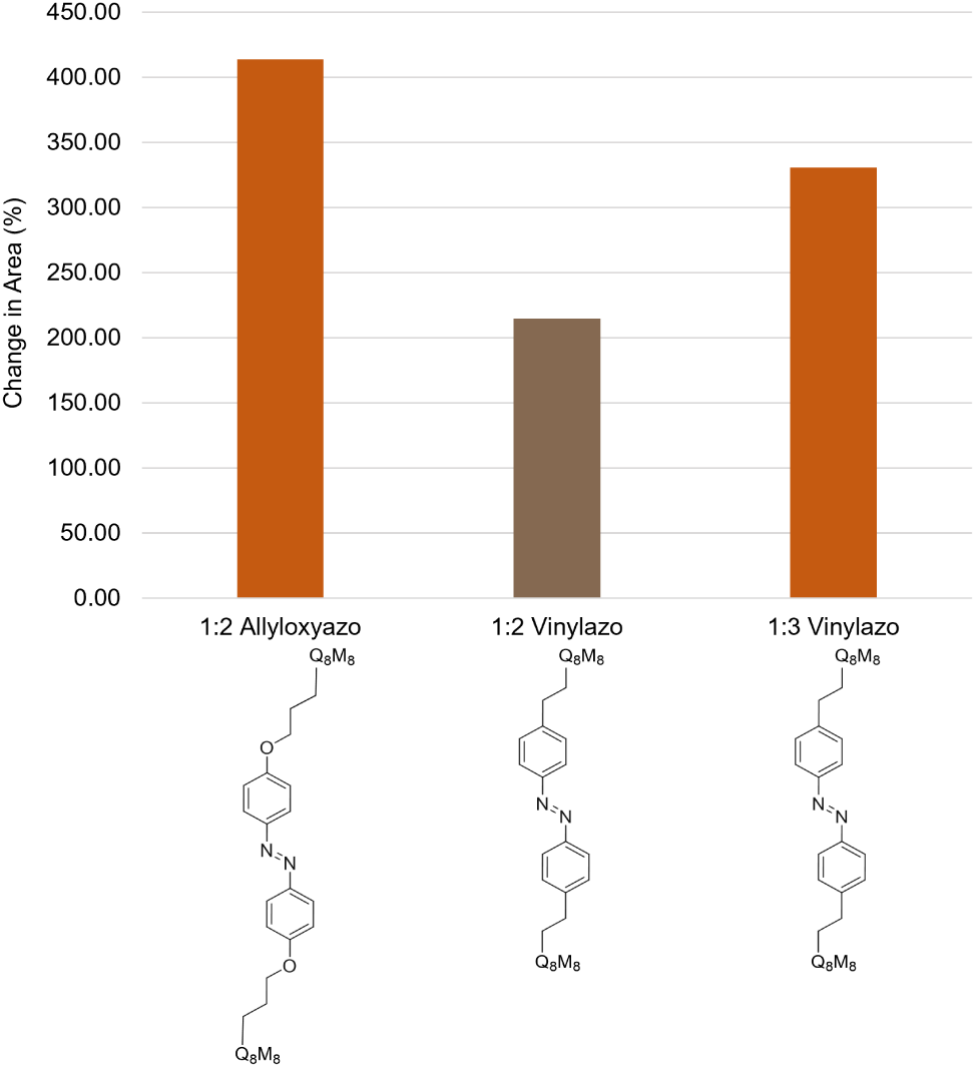
Graphical comparison of 1:2 DAA, 1:2 DVA, and 1:3 DVA gels, showing the change in observed surface area when swollen in DCM. Q_8_M_8_ in the figures represents the silsesquioxane attachment—data derived from Table 1.

The initial swelling efficiency of the DVA and DAA systems in different solvents is given in Table S3, and a graphical representation is shown in Figure 9. In this reflective analysis, using DCM as the maximum for each material, there is a ∼57% difference in toluene swellability between the 1:2 and 1:3 DVA systems, with the former preferring acetone over toluene. Still, the latter swells more efficiently into toluene than the DAA material (∼6%). Additionally, in this direct comparison, the 1:3 material has a higher preference for THF than the 1:2 DVA gel but swells only slightly less than the DAA material (∼1.3%).

**Figure 9:**
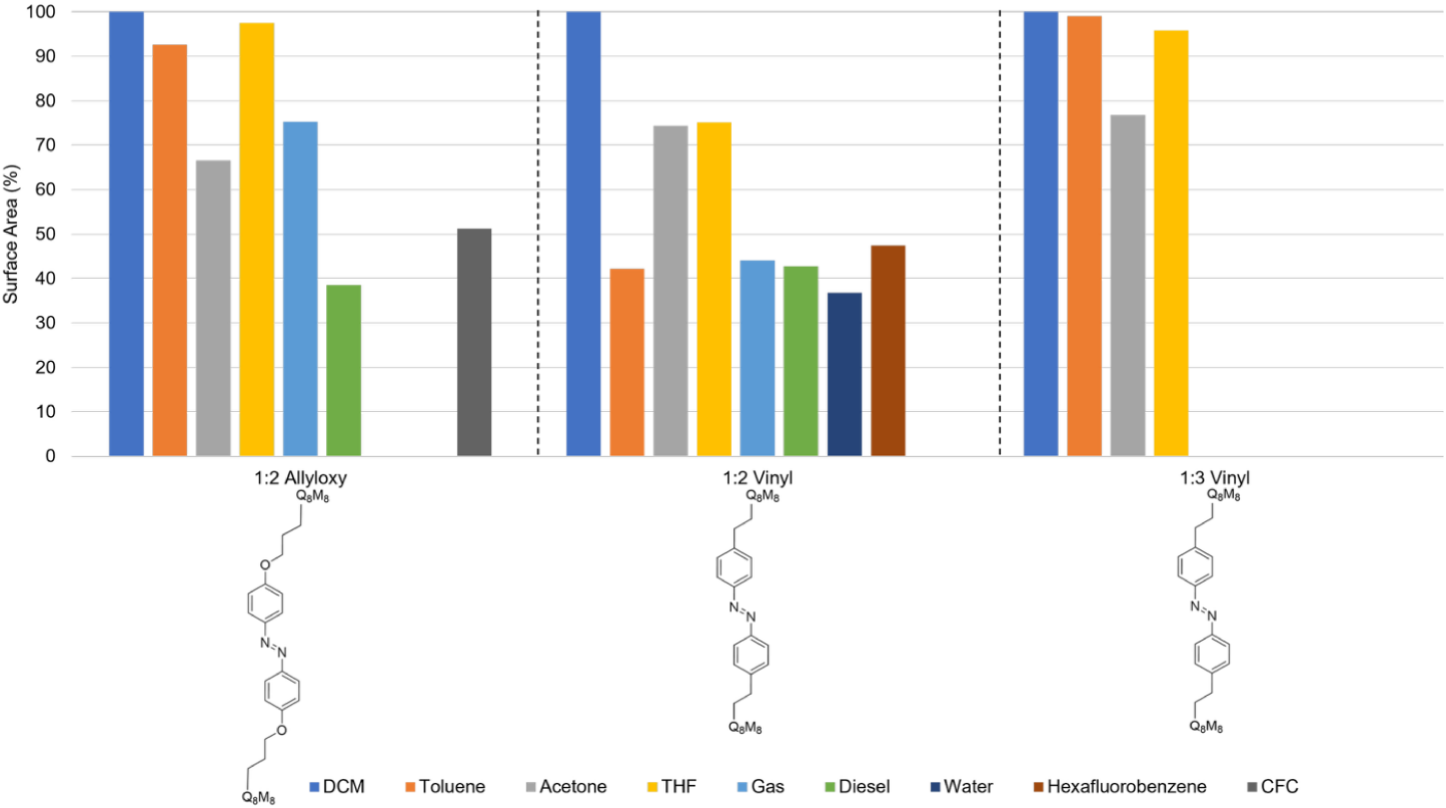
Initial, unactuated surface areas of each gel in the given solvents, using each material’s surface area in DCM as the reference maximum. Graph derived from Table S3.

The effect of photoswitch density becomes apparent when the maximum swell of the DAA material is used to adjust both DVA gels. Using the comparative maximums of the gels shown in Table 1, the surface area of each DVA material, when swollen in different solvents, changes substantially, with the DCM maximum of the 1:2 DAA gel dropping to ∼52% and the 1:3 gel dropping to ∼80%. Figure S14 and Table S4 provide a visual model showing a significant difference in relative swelling efficiencies. The 1:2 DVA system is the least efficient, with all non-DCM solvents causing swelling between ∼19–40% of the DAA system. Counterintuitively, the 1:3 system shows greater swelling in all four solvents (∼60–80% of DAA) and only slightly lower efficiency for acetone (∼5%). This provides some insights into the internal swelling mechanisms of these materials. In the DAA system, the oxygens of the allyloxy groups allow for rotation and less strain, resulting in a more adaptable/expandable internal structure. The 1:2 gel shows lower overall swelling, which may arise from variations in trapping efficiency due to polarity effects and the larger spacing between cross-linkers. The 1:3 DVA system shows greater swelling, likely due to its greater ability to fill smaller voids formed by higher cross-linking, providing more structural stability to the swollen material.

### Photoactuation, Efficiency, and Mechanics

The maximum swelling among all three gels was determined in the 1:2 DAA system, and this value was used to generate the comparative analysis mentioned above. The solvent photoactuation analyses were conducted for 3 UV–visible light cycles (see Experimental Methods). Examples of the actuation process are shown in Figures S15 and S16, where the 1:2 and 1:3 DVA systems are cycled in DCM, toluene, and acetone. The surface area of these results was derived to detail the size changes introspectively (Table S5) and comparatively with the DAA system (Table S6).

When the changes in surface area are plotted, it becomes easier to see some interesting occurrences (Figure 10). The 1:2 DVA system has the most significant initial actuation to 67.6% of its initial area, a change of 32.3% [16.7% vs DAA, accounting for max swell potential] in DCM, with a final expansion recovery to 85.2% of its maxima after 3 cycles. This suggests the material may have been highly strained upon formation, causing a greater contraction, and the internal structure relaxed over the subsequent 2 cycles. An actuation of ∼15% of its maximum is relatively high, as the DAA system boasts an average 18% reversible actuation;^5^ however, the 1:2 DVA has demonstrated the most significant initial shrinkage in response to light, indicating potential for efficient solvent release. The results from DCM UV–vis cycling (Figure 5A) correlate with the changes in swelling size; however, they show more consistent cycling between Trans and Cis isomers, suggesting that network structural changes occur beyond those strictly due to isomerization. In the 1:2 DVA gel, both THF and acetone swell the material to ∼75% [∼38%] of its maximum initial swell; however, actuation causes a steady increase in THF of +3.4% [+1.7%] and a decrease in acetone of −15.1% [−7.8%]. This suggests the 1:2 DVA system has an overall preference to swell in both DCM and THF; however, all other solvents tested caused the material to decrease in size or lose actuation, with the lowest material surface area resulting from water (36.8%, or 19.10% vs DAA) and minimal alteration to the size of the material during photoactuation (<1%). A poor interaction with water is ideal for a pollutant sponge, as it suggests selectivity for the uptake of compatible pollutants and lower water absorption.

**Figure 10:**
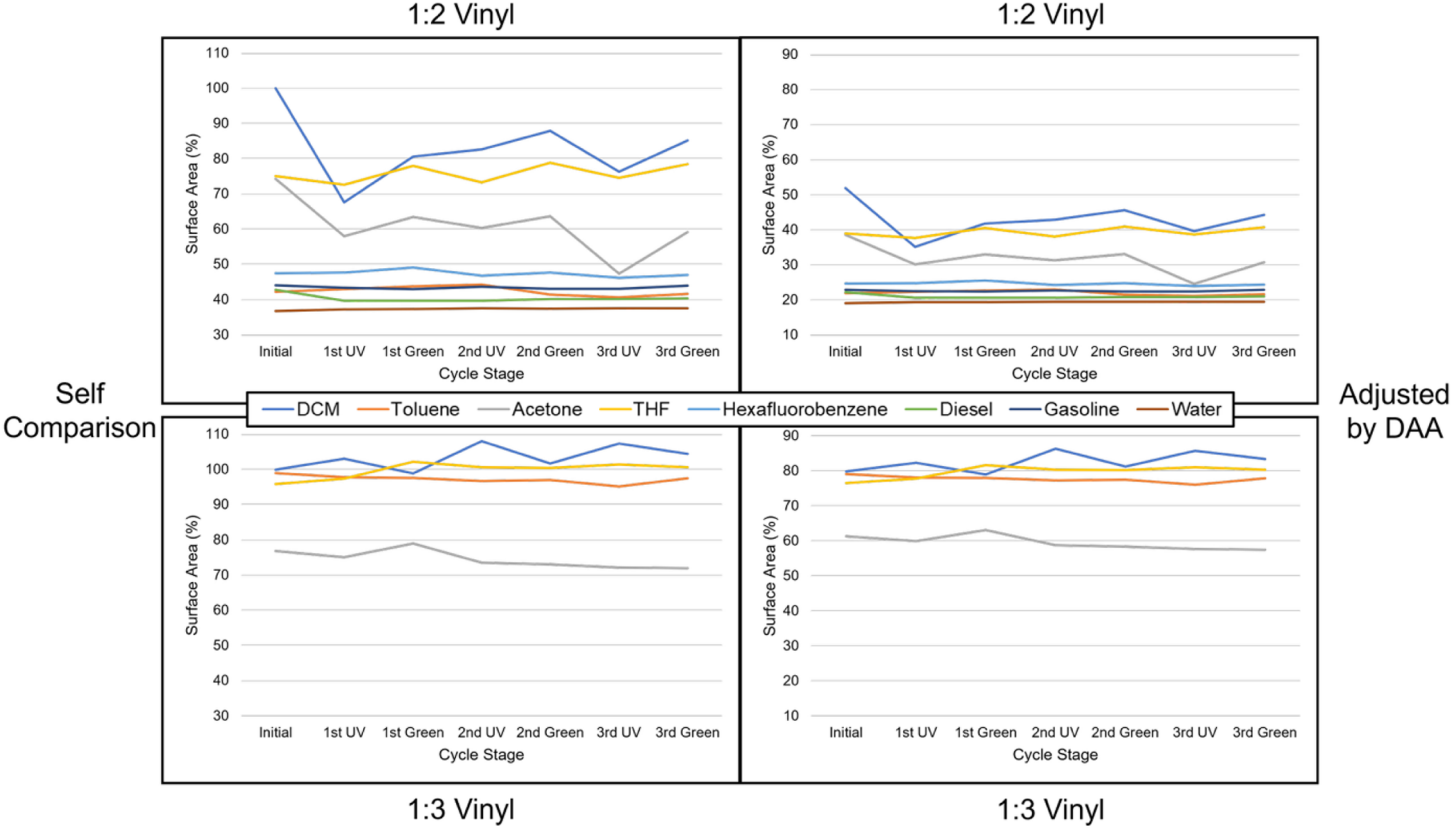
Plots depicting the actuation of the 1:2 and 1:3 DVA gels in various solvents across three cycles. Graphs on the left indicate the reflective analysis, while the graphs on the right have been adjusted for comparison against DAA. Data was extrapolated from Tables S5 and S6; ranges of 20–110% (left) and 10–90% (right) are shown.

In DCM, the 1:3 DVA gel exhibited an inverted action, expanding to ∼108% under UV light and shrinking under visible light, reaching 104.4% of its initial surface area after all cycles. By UV–vis (Figure 5B), this is surprising since trans–cis isomerization takes place, albeit not to a complete extent, showing that some other effect, potentially polarity or network relaxation changes, accounts for this phenomenon here. This system has a higher cross-linking density (i.e., more attached points), which may lead to greater interlocking and internal strain in the trans form than in the cis isomer, at least in DCM. Still, since the networks are formed randomly, only inferences can be made in this current study. While the 1:3 DVA material swelled efficiently in toluene (∼99.0%, 79.88%), there was minimal response to actuation, with a total change in material surface area of 1.5% [1.2%]. A similar effect is observed in the acetone analysis, where the material exhibits minimal size shift of <5% [<3.8%] across all cycles. In THF, the 1:3 gel undergoes a larger increase after the first cycle, to 102.2% (∼6.4%, 5.1%), but remains relatively unresponsive, returning to ∼4.8% [3.8%] of its initial size. These analyses show a lack of photoresponse in the 1:3 DVA system compared to both the 1:2 DVA and DAA materials, indicating that this system may have too many photoswitches to behave as a sponge but may be used as a photoswollen absorbent, leaving an area for future investigation.

DMA was conducted on both the 1:2 and 1:3 DVA systems in the swollen form to understand the fundamental mechanical properties of the gels. To achieve this, the gels were submerged in a solvent bath during analysis to prevent drying out. THF was chosen for analysis as the gels needed to remain swollen during analysis, and THF offers optimal compatibility with all studied systems. Once loaded, samples were analyzed without irradiation for 10 min, then underwent three cycles of UV (365 nm, 20 min) and visible (515 nm, 20 min) light exposure (Figure 11). This allows detection of differences in the storage and loss moduli of each material resulting from azobenzene isomerization, which makes the material firmer under UV due to reduced interstitial space. The storage modulus of the 1:2 DVA gel is initially ∼10 MPa, then fluctuates between ∼10.7 (365 nm) and 8.5 MPa (515 nm) during actuations. In the 1:3 DVA gel, the base storage modulus is ∼7.2 MPa before irradiation, increases under UV to ∼8.1 MPa, and decreases to ∼6.3 MPa with visible light after a cycle. This material shows a gradual decline in fluctuations, with the final actuations ranging from 6.3 to 7.6 MPa. This reduction in efficiency suggests either actuation hysteresis or a reduced ability of the increased photoswitch ratio to actuate the material. In both cases, the material’s swelling makes the data appear “noisy”. In all cases, the loss modulus does not fluctuate with actuation and remains low due to energy dispersion to the solvent. Compared with 1:2 DAA (160 to 500 kPa UV/vis cycles),^5^ the new DVA gels exhibit an increase in storage modulus by over an order of magnitude, thereby enhancing their applicability to photoinduced mechanical actuation systems such as soft robotics. Comparison with UV–vis cycling experiments (Figure 5) over the first three actuation cycles is consistent with the findings from DMA and is more consistent than the swelling results. Unfortunately, attempts to study the dried-out DVA gels by DMA were unsuccessful.

**Figure 11:**
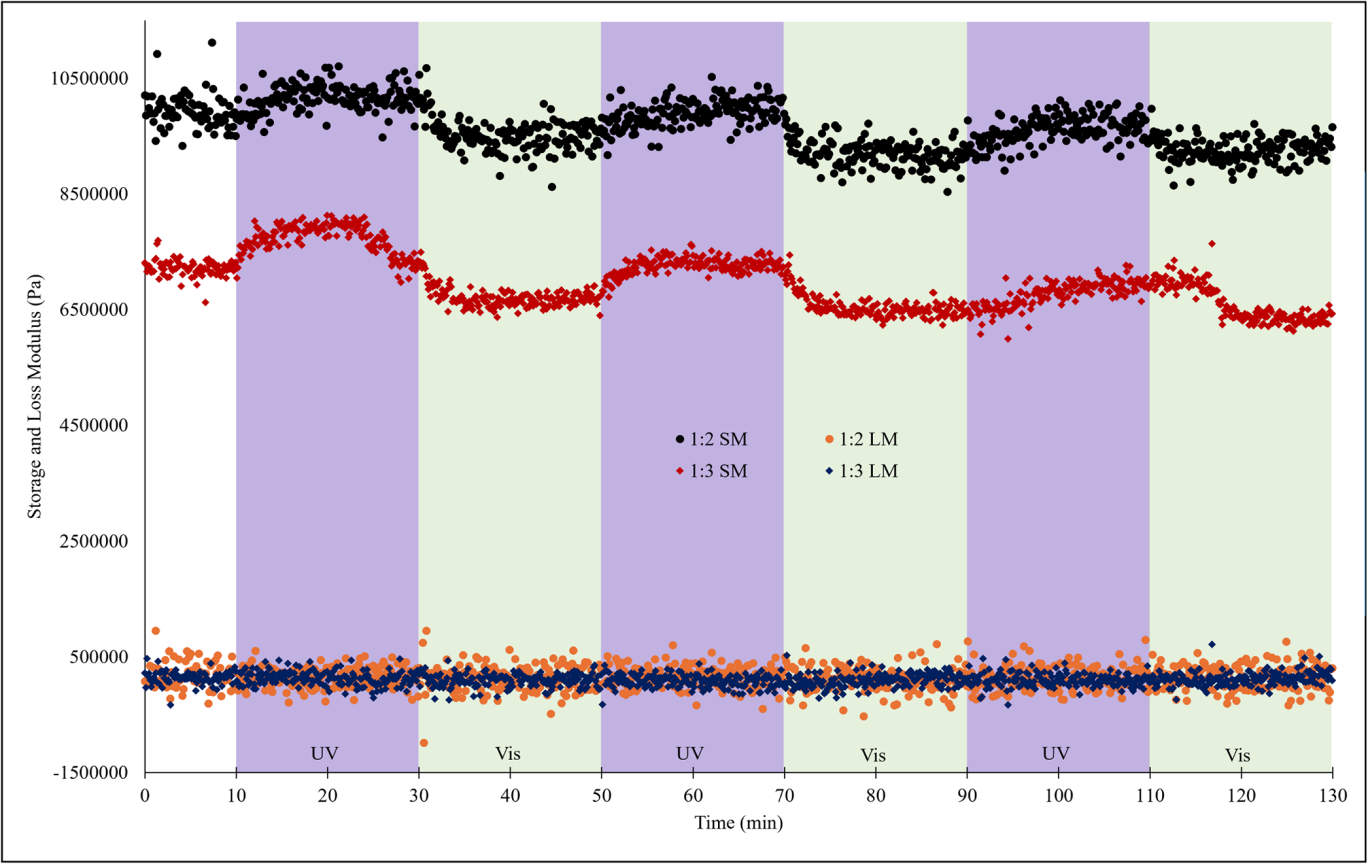
DMA actuation measurements of the DVA gels showing the changes in the storage modulus (SM) over time when exposed to UV (365 nm) and visible (515 nm) light.

Multiple experiments were conducted to understand the structural and polar inner workings of these materials, particularly regarding static-to-UV size/shape changes. For example, to understand the change in structure upon light irradiation and why solvents are expelled on demand so readily. Five experiments, including Ultrasmall Angle Neutron Scattering (USANS),^50,67,68^ Small Angle Light Scattering (SALS),^51^ polarity study with Reichardt’s dye (B30), Small Angle X-ray Scattering (SAXS), and contact angle measurement, were performed.^53^ The first three methods failed to provide a greater understanding of pore or swollen structural features. SALS and USANS (Figures S17–S19) suggested that materials may be so dispersive in structural connection points at larger length scales that any information is difficult to discern amid instrument backgrounds, regardless of irradiation wavelength. This means that the materials likely behave less like a porous network and more like a swollen, randomly (dis)­ordered polymer, causing significant variation in scattering, or that we are missing the size range over which the features exist.^69^ However, some discernible features were found using SAXS analysis of the gel materials as discussed below.

To assess whether smaller structural changes were responsible for the observed macroscopic properties and/or porosity, small-angle X-ray scattering (SAXS) was used in a simple,^34,52,70^ noncomprehensive manner to draw structural inferences. SAXS analysis was conducted on 1:2 DAA, 1:2 DVA, and 1:3 DVA gel samples, maximally swollen in toluene (Table 1), both with and without UV irradiation, to further investigate changes in these systems and the cause of solvent expulsion under UV irradiation. Swollen samples were used to show the system’s behavior under ideal conditions,^71^ but also because of difficulties with the large amounts of material needed to effectively fill the sample holder with dried-out samples. The SAXS data are given in Figure 12, Table 2, and Figures S20–S37, which show log­(intensity) vs q (Figure X), Guinier analysis, GNOM analysis, and Kratky plots for each sample.^63,64,72–74^ Overall, the intensity vs q plots for all samples show little evidence of aggregation, typically exemplified by upturns at low-q, and no dramatic shape changes between non-UV- and UV-irradiated samples. Guinier analysis (low-q linear fitting) provides a relatively good fit to the data for determining *R*_g (Guinier)_, as shown in Table 2 (Figures S26–S31), with values 77–92 Å range or average distances between scattering centers of ∼15–18 nm, much beyond the length scales of azobenzene bridges alone ∼1.4 nm trans (full linker),^20^ showing significant swelling. The data suggest no clear correlation between *R*_g_ (radius of gyration, average scattering radius), whether the samples were UV-irradiated or not, indicating that any induced structural changes are minimal at the ∼1–100 nm length scale. Interestingly, there is a drop off in the slope toward the region intermediate between the Guinier and Porod region (∼0.03 to 0.5 q), of the scattering profile (Figure 12) for UV irradiated samples, which suggests reorganization in the structure,^75^ even if the overall dimensions in the Guinier and Porod regions do not change much by modeling. The Guinier data are also in reasonable agreement with the GNOM-IFT analysis (Figures S32–S37), which uses indirect Fourier transforms to better fit noisy scattering data.^64,76^ The *R*_g_ values are similar across methods, with no clear correlation between UV and non-UV studies. The fits from this model yielded “reasonable solutions” to the data, so the overall fits are not perfect and are slightly above the ideal χ^2^ value of 1 (Table 2). The GNOM program provides additional information about the overall Pair Distance Distribution (P­(r)), i.e., the distances between electron centers such as highly X-ray-active silicon atoms (Figure S22).^76,77^ From this analysis, we observe a well-shaped bell curve that approaches the baseline at higher r (radius) values, suggesting roughly spherical or globular structures. Only subtle, inconsistent differences are observed with UV irradiation, but nothing that would be considered a major change of 10 s of nm to effect macroscopic changes. The distribution also approaches the baseline without long tails, suggesting that highly elongated or irregular structural motifs are absent. The data suggest a wide range of distances, consistent with the presence of both cages and spaces between them. The *D*_max_ values represent the maximum distances observed in the system between any two centers of electrons (mass). For the DVA systems, *D*_max_ decreases with UV, whereas for the DAA system, *D*_max_ increases slightly with UV, suggesting both are within a range of error or their mechanisms of action are truly different. One surprising observation from the data is that, across all structure size parameters (*R*_g_, *D*_max_), the DAA (longer azo bridge) system was smaller than the DVA (shorter azo bridge) system, suggesting that the flexibility of the longer linker may lead to smaller spacing in the material.

**Figure 12:**
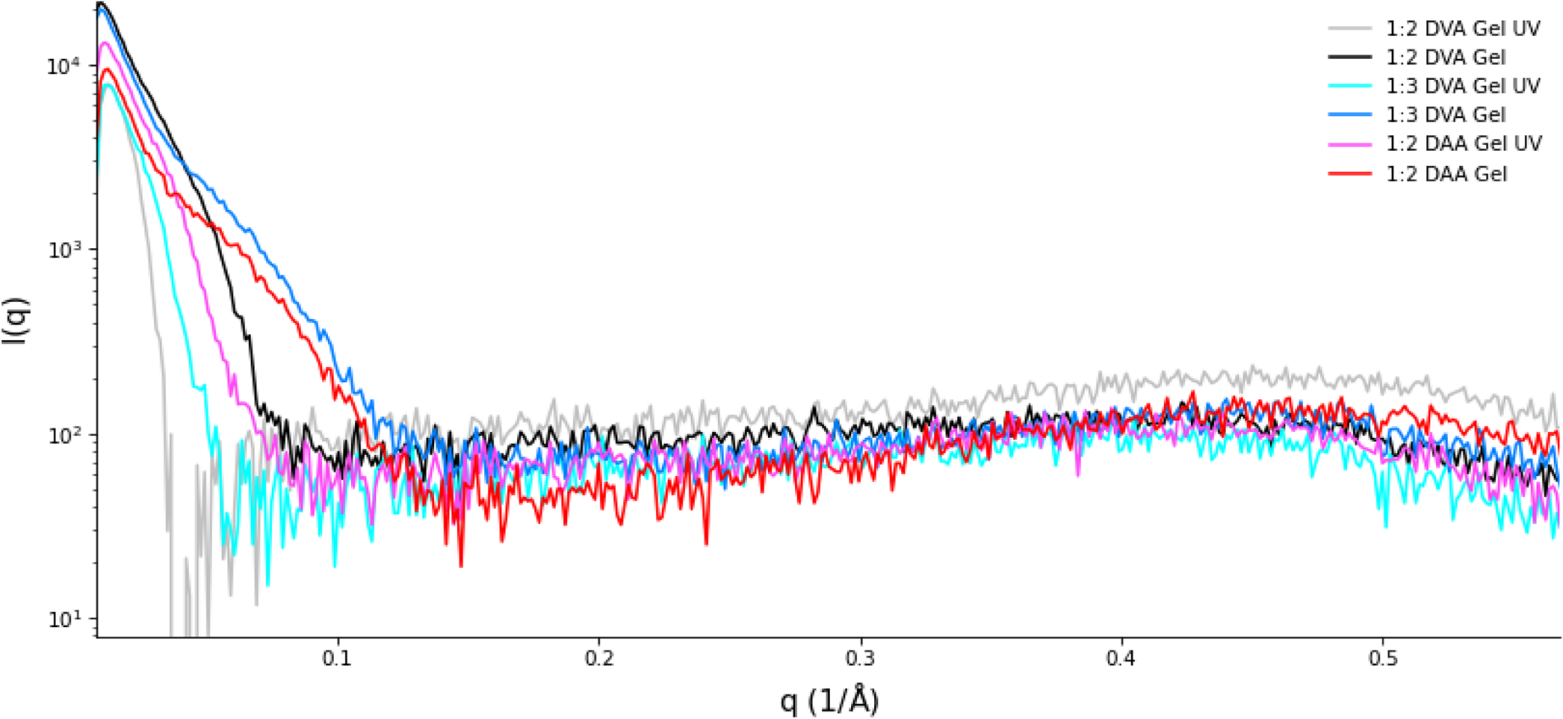
Log I­(q) versus q (Å^–1^) for gel samples swollen in toluene, encapsulated into a Mylar pouch to prevent evaporation, and compared between static and UV-irradiated forms.

**Table 2:** SAXS Analysis Data for Swollen and Swollen + UV-Irradiated Gel Samples

	*R*_g (Guinier)_ (Å)	Guinier *R* ^2^ (Fit)	I(0)	*R*_g (GNOM)_ (Å)	GNOM χ^2^ (Fit)	*D*_max_ (Å)	*V*_p_ (Å^3^)
1:2 DVA	83.6 ± 0.9	0.996	2.76E^+4^ ± 3.07E^+2^	99 ± 1.8	1.002	346.0	1.46E^+5^
1:2 DVA UV	92.3 ± 0.9	0.996	1.31E^+4^ ± 2.53E^+2^	90.2 ± 0.64	1.36	231.0	2.40E^+6^
DA 1:3	86.0 ± 0.7	0.996	2.52E^+4^ ± 2.11E^+2^	96 ± 1.84	1.23	343.0	1.19E^+5^
DA 1:3 UV	79 ± 1.3	0.993	1.09E^+4^ ± 2.06E^+2^	98 ± 3.69	1.23	337.0	1.63E^+6^
DAO 1:2	77.0 ± 0.7	0.996	1.26E^+4^ ± 1.52E^+2^	79.6 ± 0.74	1.21	218.0	4.78E^+5^
DAO 1:2 UV	77.7 ± 0.7	0.996	1.31E^+4^ ± 2.53E^+2^	89 ± 1.35	1.18	286.0	1.21E^+6^

To obtain a clearer overall picture of potential changes in the system, a Kratky and Porod volume analysis was conducted.^78^ Figures S23–S25 show the dimensionless Kratky plots, which demonstrate that samples under both conditions remained compact, likely globular, and well-organized, as no plateaus or continuous rise are observed in the high-q data and no upturn at low-q is observed, indicating no significant aggregation or clustering with UV irradiation. However, two distinct peaks are observed in the Kratky plots for each sample, indicating a change not apparent from other analyses but potentially correlating with the change in slope in the mid-q region in Figure 12. The peaks in the high-q region (smaller structural components such as periodicity and cage components) show low correlation between UV and non-UV, whereas the peaks in the low-q region tend to shift toward larger structure-size parameters (to the left) under UV irradiation in all cases. albeit subtle. The increase in a structural size/compactness parameter was very surprising since this runs counter to what would be expected based on the macroscale properties of the materials. We have observed behavior that follows this in dried films of 1:2 DAA, where a UV-irradiated film appears to expand rather than shrink,^5^ and there seems to be a precedent in the literature for this occurring in azobenzene systems.^79,80^ We have further confirmed this feature by examining the Porod volume (*V*_p_, Table 2), which provides information on the swollen hydrodynamic volume of a network structure.^81^ In each of the UV irradiation cases, an increase in the *V*_p_ volume by about an order of magnitude, 10^5^ to 10^6^ Å^3^ was observed. Both run counter to the idea that UV irradiation should reduce the structure’s overall volume, as observed at the macroscopic level. The static non-UV *V*_p_ also deviates slightly from the swell results in Table 1. The 1:2 DAA has about 2x the swell capacity and more than 2x the *V*_p_ of the 1:2 DVA system, but the 1:3 shows greater swell and a lower *V*_p_. However, just because they expand at a specific length scale (diameter ∼14–20 nm) does not rule out other macroscale changes in size or even polarity as the reason for solvent expulsion, but these should have been observed clearly in the laser light scattering or USANS experiments. A rarer alternative explanation is that the electron-density contrast between the scatterers and the matrix could increase due to UV irradiation-induced shrinkage, resulting in an observed increase in the Porod volume, or a “springback” effect, which could stem from excessive UV irradiation of the sample over 12 h.^71,82^ In this case, we would expect to see an increase in scattering I(0), but the scattering stays mostly the same or decreases with the UV systems.

For a clearer understanding of what is happening in the samples across dried, static-swollen, and UV environments, synchrotron X-ray imaging, solid-state film analysis, or further SANS analysis may be needed to achieve better signal-to-noise contrast and to verify or refute the findings here. The gel actuation performance is likely a combination of small structural changes acting in unison and minor polarity differences induced by trans–cis isomerization, though this remains speculative.

To investigate changes in polarity, two studies were conducted. The first was to use Reichardt’s dye to investigate the polarity changes associated with trans–cis isomerization. It was found that the dye is rapidly bleached by the azo system, and we were unable to use dye color changes to discern changes in dipole and internal polarity upon UV irradiation (Figures S38–S40). Since this experiment failed to provide insight into the internal polarity, we sought to investigate the surface polarity of the dried-out DCM gels, at least to correlate trans vs cis effects on water contact angles (CA) as a proxy for polarity. We examined water droplets on the surfaces of each gel material before and after 10 min of 365 nm UV irradiation. The contact angles for the gels were investigated, specifically 1:2 and 1:3 DVA (Figure S41). Though the materials are not as flat as we would prefer in the solid state upon drying, we find that between non-UV and UV irradiated (365 nm) 1:2 DVA sample ∼10° drops in CA are observed from 87 to 77°, suggesting an increase in polarity. However, for the 1:3 DVA sample (50% more cross-linked), no change was observed between non-UV and UV exposure, and the 1:2 DAA did not dry into large enough/flat enough pieces for effective analysis. Overall, the results are somewhat ambiguous but suggest that an increase in polarity with UV may be present and may help explain some of the swelling and expulsion observations observed.

### Application Performance

The 1:2 DVA material exhibits selectivity for solvents over water and has a swollen surface area 63% [32%] higher in DCM and 38% [19%] higher in THF. When an immiscible, denser solvent such as DCM is absorbed by the gel, the material behaves like the pollutant, settling below the water surface, due to DCM’s density. Actuation of gels that contain immiscible pollutants will cause the solution to form pockets around the gel. The demonstration below shows a 0.014 M 9-fluorenone in DCM solution that was swelled into the gel. UV light was used to shrink the material after encapsulation in water, thereby expelling the DCM (Figure 13). Selective removal of pollutants was demonstrated with a THF–water solution using the same dye (1 M 9-fluorenone). When a dried 1:2 DVA gel was exposed to this solution and to 515 nm light, it facilitated the uptake and removal of THF from water, as evidenced by the increased dye concentration around the gel (Figure 14). This illustrates the potential of these materials to interact preferentially, absorbing pollutants within aqueous environments, and provides insights into how they may behave when used for a specific environmental target with water. Additionally, using an insoluble dye that remains in solution demonstrates potential routes for solvent-assisted capture of solid wastes with limited water solubility and/or for their filtration.

**Figure 13:**
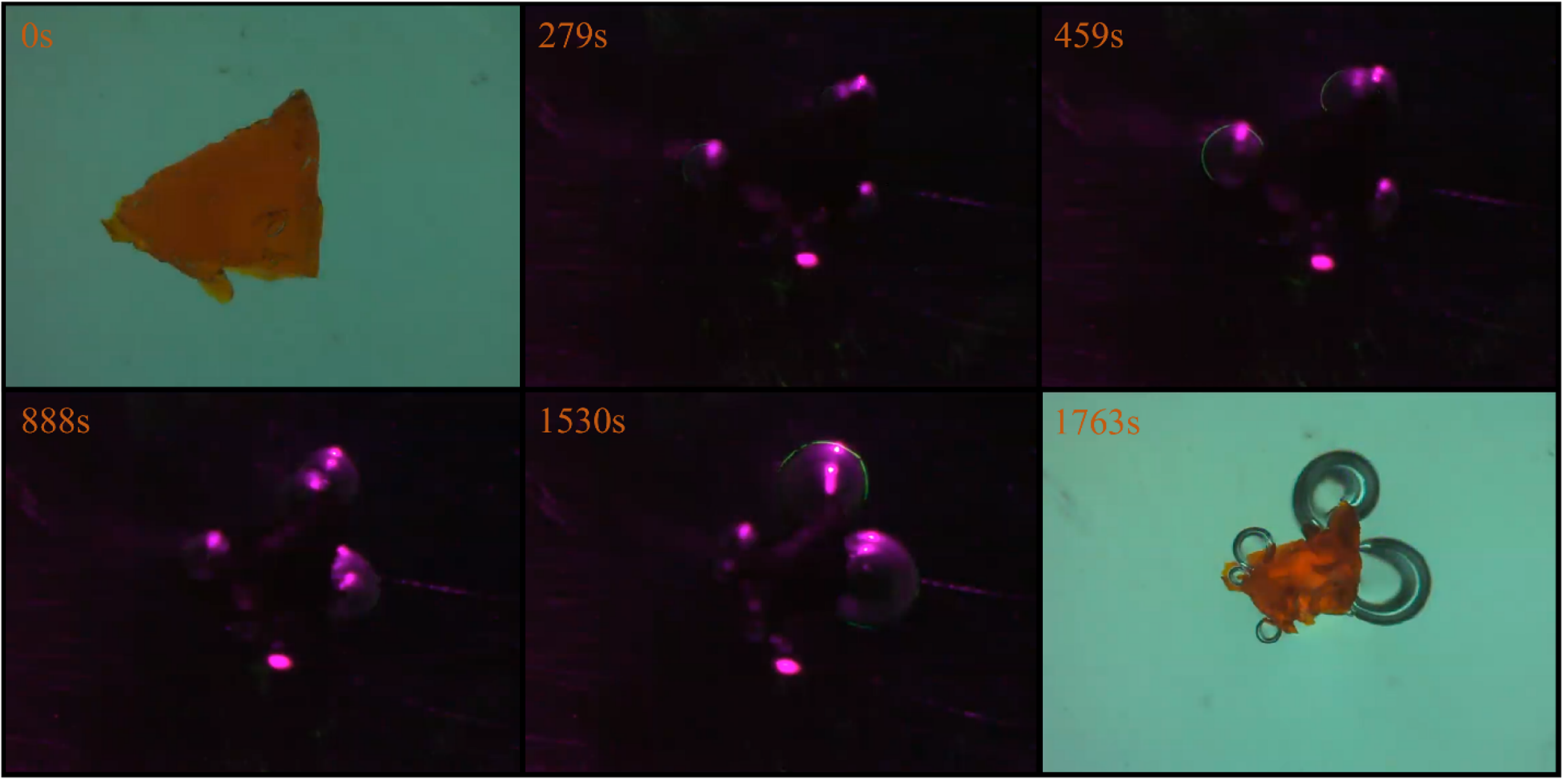
Still frame images of a 1:2 DVA gel swollen in a 9-fluorenone in DCM solution, which was encapsulated in water and underwent actuation from the trans to cis forms by UV (365 nm) light. Bubbles of DCM form around the gel within the first few minutes and continue to grow for ∼ 30 min, with some edge fluorescence of the 9-fluorenone observed on the bubbles. *Note that even without UV irradiation, some DCM does get pushed out of the gel by the pressure of being submerged in water and by the high density of DCM, but UV light aids in increasing the rate and magnitude of change*.

**Figure 14:**
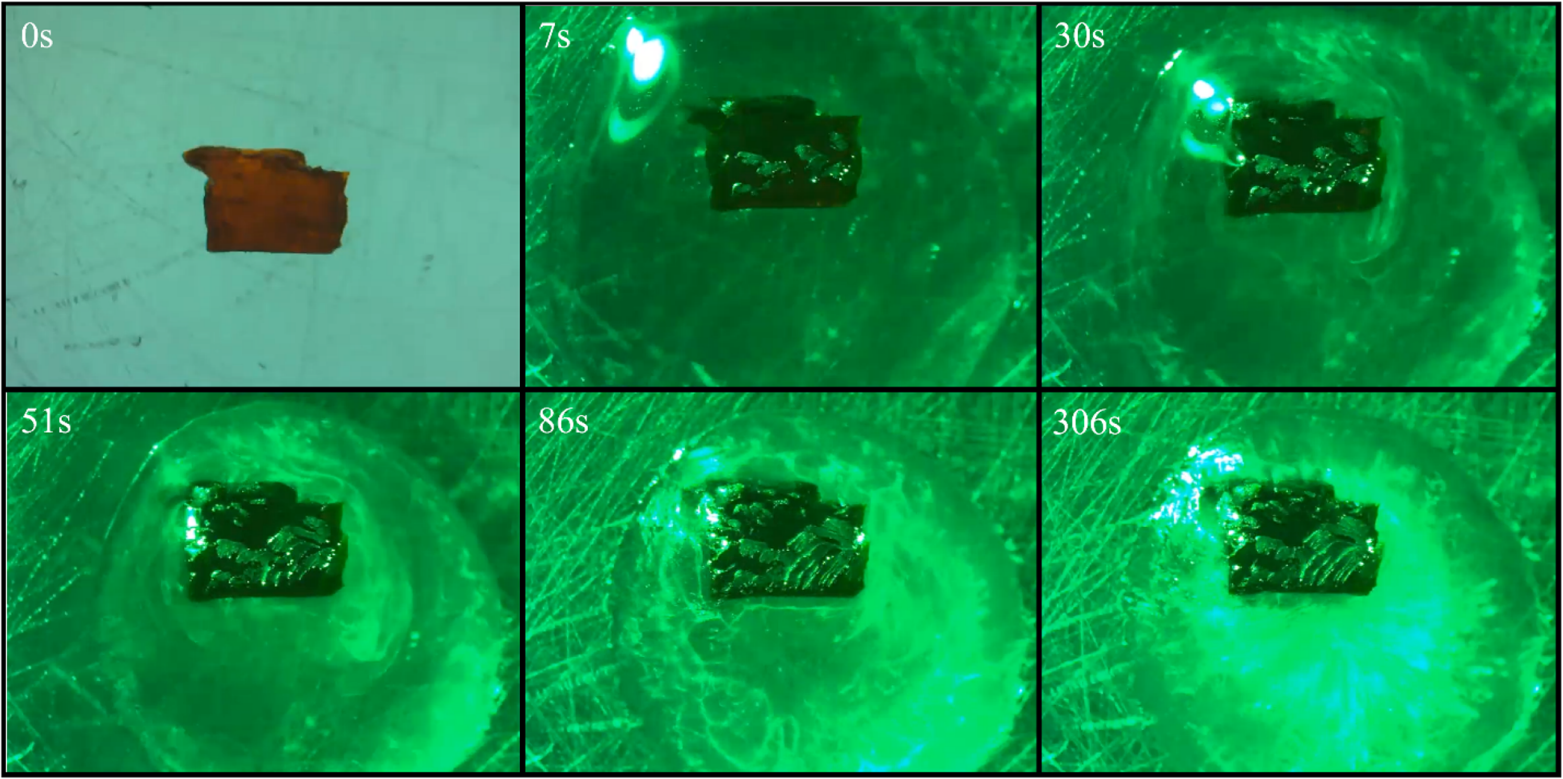
Still frame images of a dried-out 1:2 DVA gel exposed to green light (515 nm) while a 9-fluorenone solution (1:1 THF–water) was added. Within approximately 5 min of contact, the gel facilitates the removal of THF from the mixture, concentrating the dye in water. Time points are given from the time of solution addition.

## Conclusions

Investigation of the 1:2 and 1:3 4,4’-divinylazobenzene-Q_8_M_8_ (DVA) gels shows their potential as pollutant remediators analogous to the 4,4’-diallyloxyazobenzene-Q_8_M_8_ (DAA) system. The 1:2 DVA gel has exhibited a large initial photoinduced “wringing” of 32.3% [16.7% vs DAA] and 10+ cycle UV–vis reversibility in DCM, making it ideal for the remediation and expulsion of hazardous chemicals. While the 1:3 material offers lower actuation (∼50% isomerization under UV light) and reduced cycling stability (<4 cycles), it presents an opportunity for further research as a lower-photoactivation absorbent. We have also demonstrated that 9-fluorenone-containing DCM and THF solvents can be selectively retained/captured from aqueous solutions, thereby establishing the environmental potential of the 1:2 DVA gel. The authors believe that advancing the study above may help reduce the hazards associated with the cleanup of manufacturing or environmental contamination involving DCM and other hydrocarbon substances. Two analogs of azobenzene-coupled silsesquioxane sponges have been discussed above, each with its own benefits and caveats. Studies to determine structural and internal polarity changes have been challenging, but knowledge gained from SEM, SAXS, and contact angle experiments suggests the presence of large pores, swellable structures, and a potential decrease in polarity. We will continue to explore other methods to better understand the fundamental aspects of shape-change efficiency. The developments presented have expanded the repertoire of azobenzene-based functional smart materials and opened the door to further study of targeted pollutant removal, biotechnology, and increased overall actuation potential.

## Supplementary Material



## Notes

The Supporting Information is available and includes additional gel swelling, actuation, and characterization data, including images, tables, UV–vis cycling behaviors, ^1^H NMR, Mass Spectrometry, FTIR analysis, reduced USANS data, SALS, SAXS, Water Contact Angle, and Reichardt’s dye studies. Raw data available upon request­(PDF)

## #

Cory B. Sims – Materials Science and Engineering, University of Michigan, Ann Arbor, MI 48109, United States

## ∇

Herenia Espitia Armenta – Danish Polymer Center, Danish Technical University, Lyngby, Denmark
