# Supplementary material for: Divinylazobenzene-Q-Cage Silyloxy-Silsesquioxane-Based Reversible Photoactuatable Network Polymer Sponges
Source: Macromolecules. 2026 Jul 21;59(15):8591–606. doi: 10.1021/acs.macromol.5c03635 (PMC13472184; doi:10.1021/acs.macromol.5c03635)
Supplement: Supplementary file 1 [file ma5c03635_si_001.pdf]

Supporting Information

Divinylazobenzene-Q-Cage Silyloxy-Silsesquioxane-based Reversible Photoactuatable Network Polymer Sponges

Sims, C.B.; Chandler, E.T.; Espitia Armenta, H.; Erfani Jazi, M.; Deller, A.C.; Furgal, J.C.*

Department of Chemistry and Center for Photochemical Sciences, Bowling Green State University, Bowling Green, OH 43403, USA

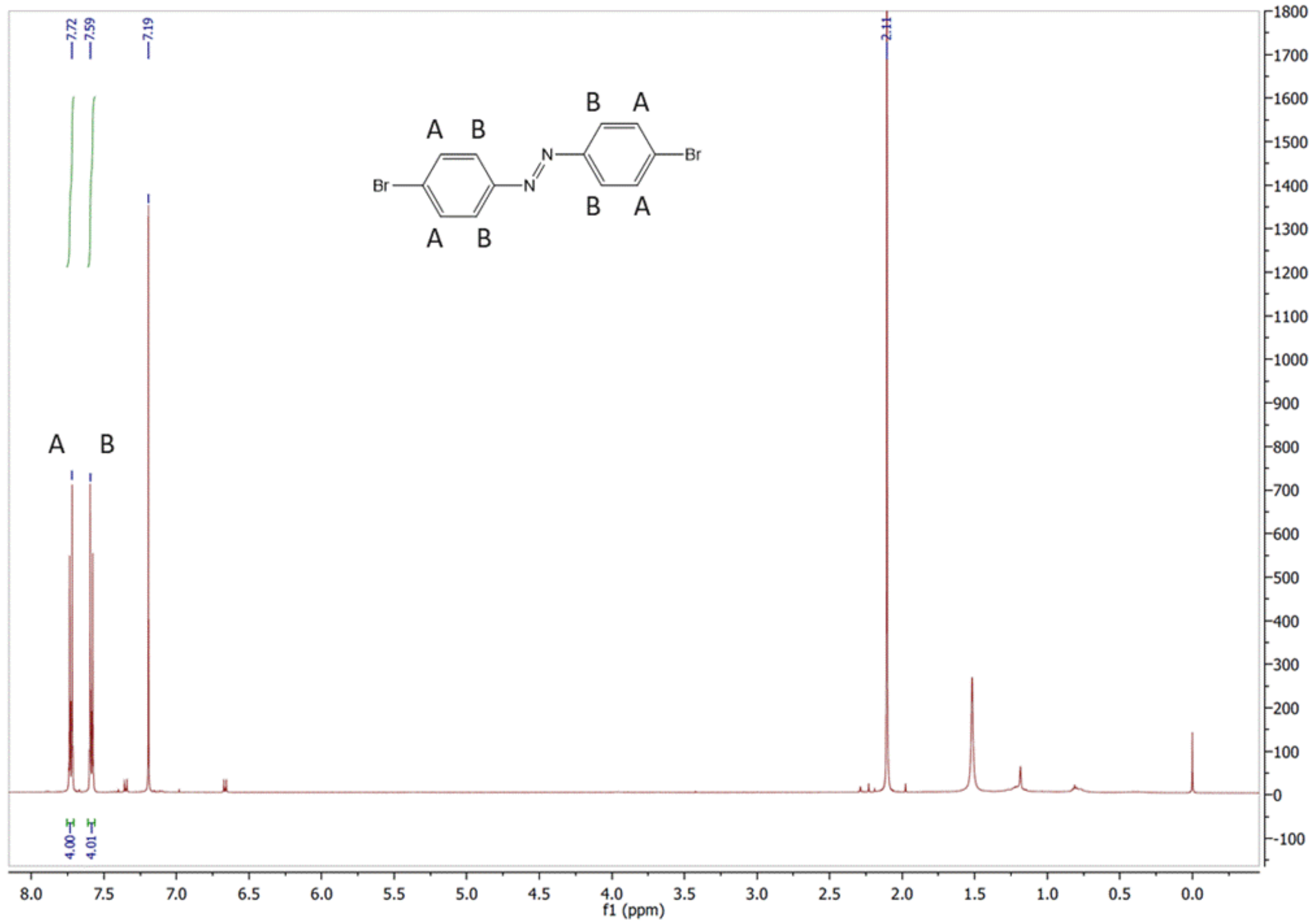

Figure S1. ^{1}H NMR of the 4,4'-dibromo-azobenzene monomer.

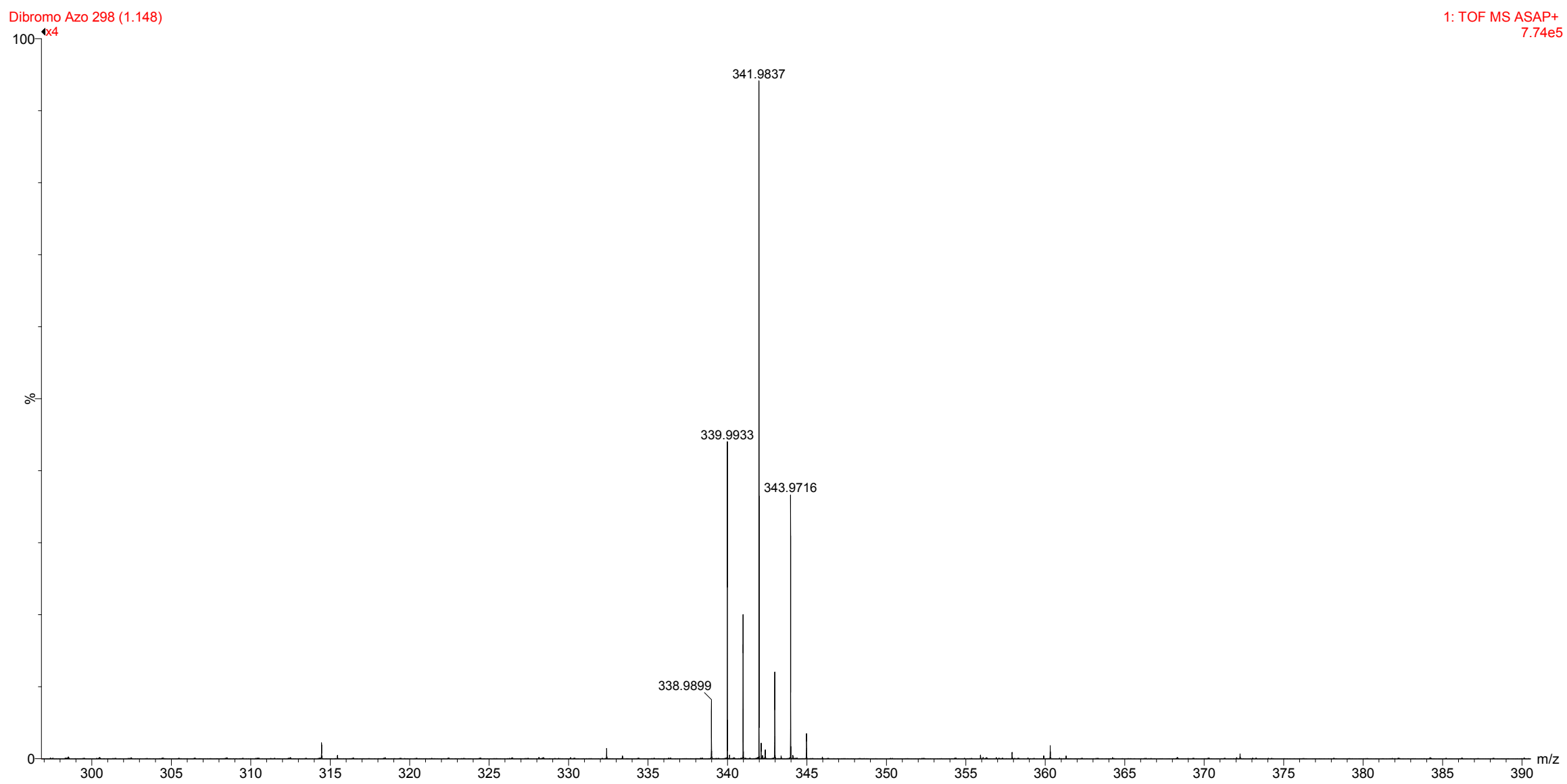

Figure S2. ASAP-HRMS analysis of the 4,4'-dibromo-azobenzene monomer.

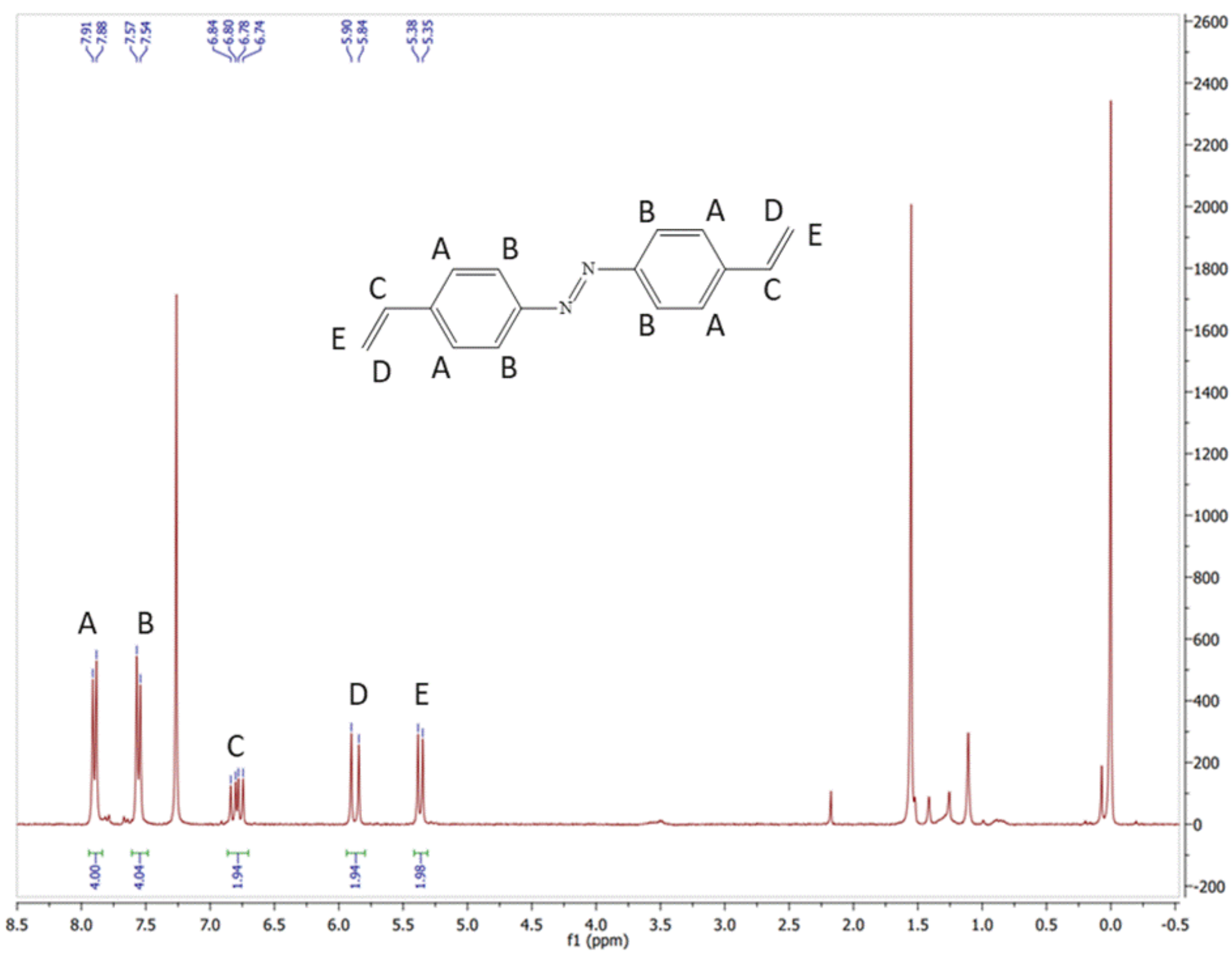

Figure S3. ^{1}H NMR of the 4,4′-divinyl-azobenzene monomer.

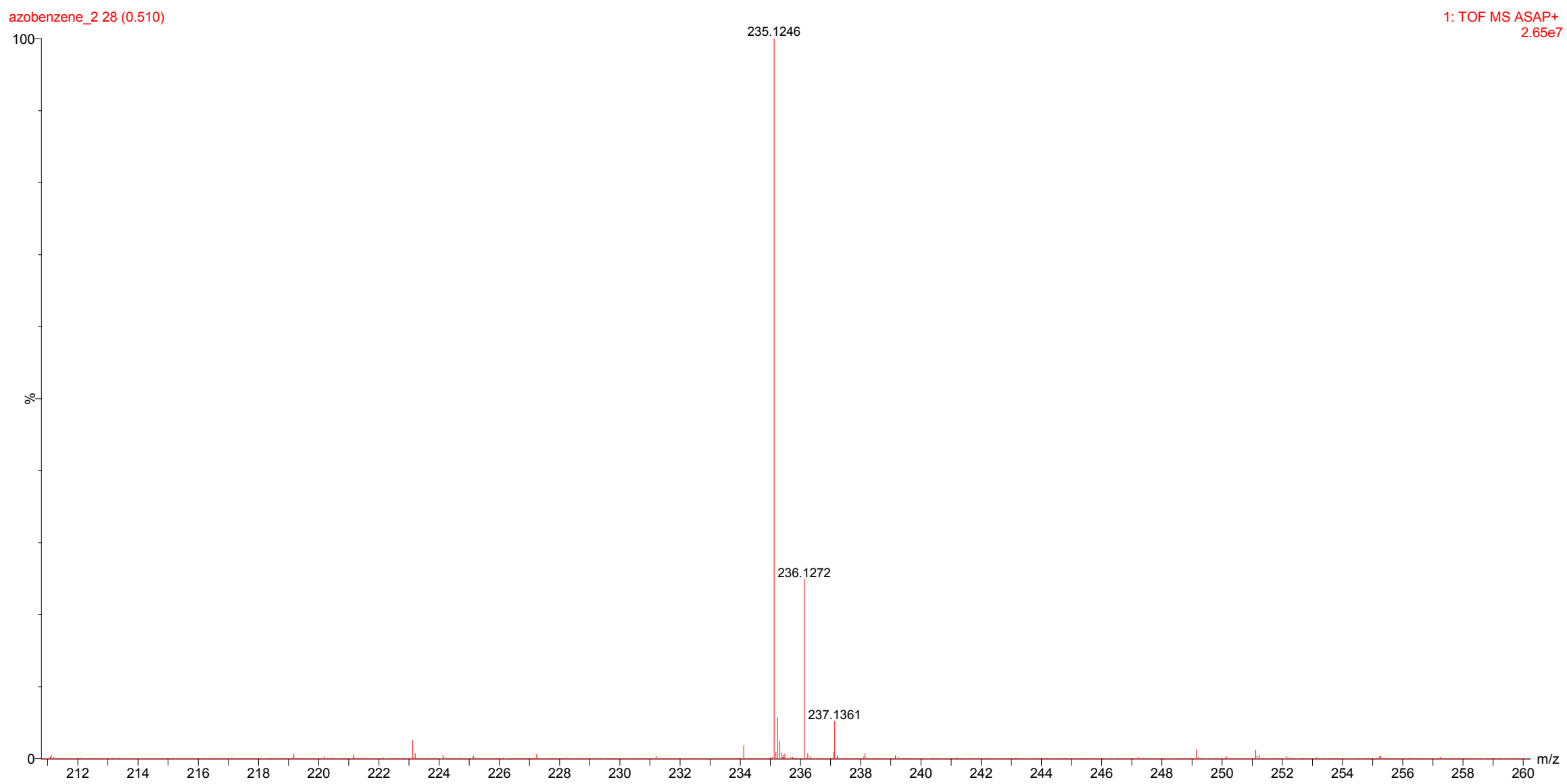

Figure S4. ASAP-HRMS of the 4,4′-divinyl-azobenzene monomer.

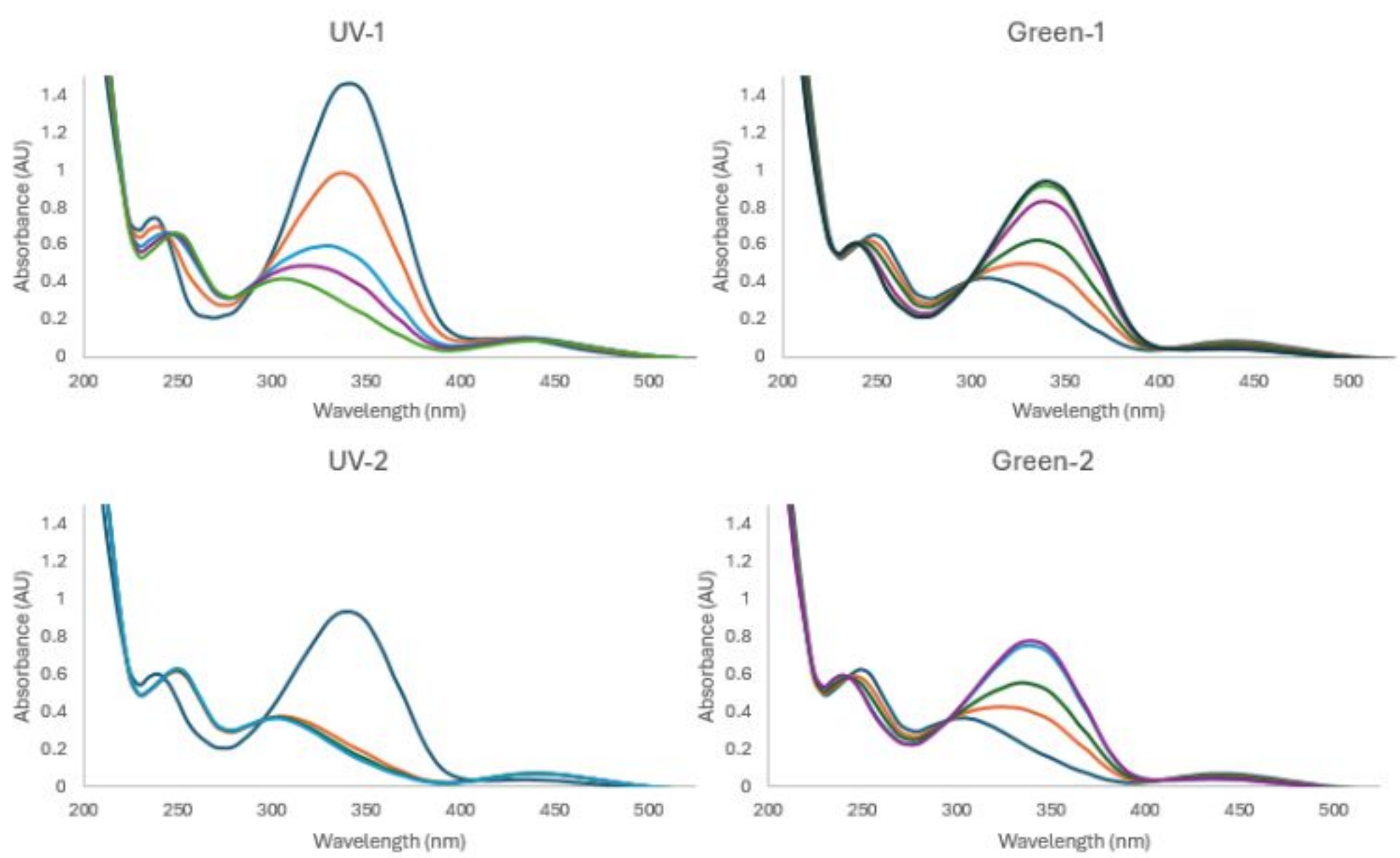

UV-5

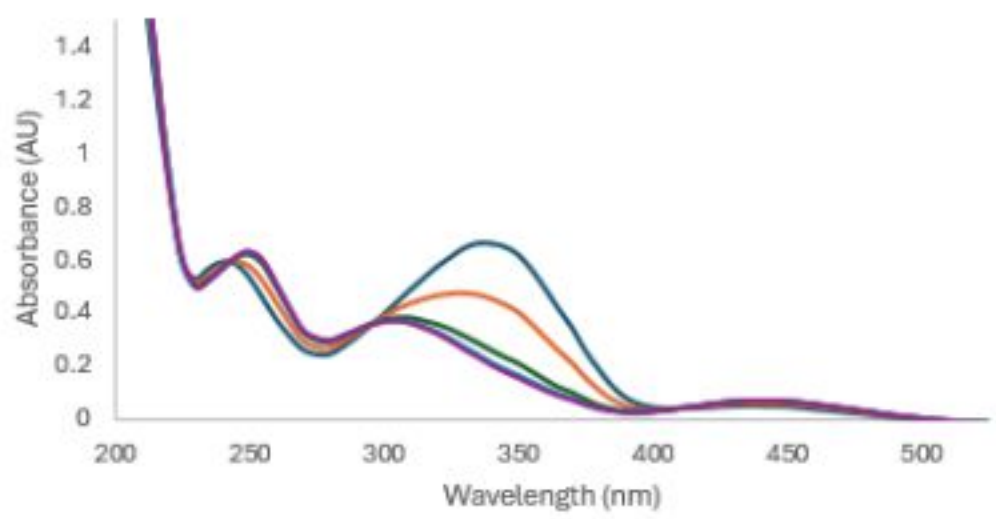

Green-5

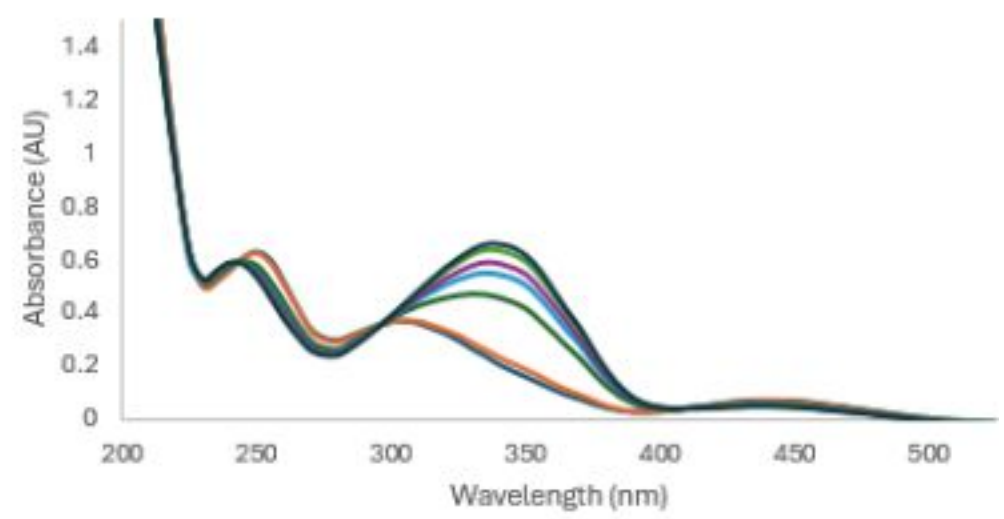

UV-6

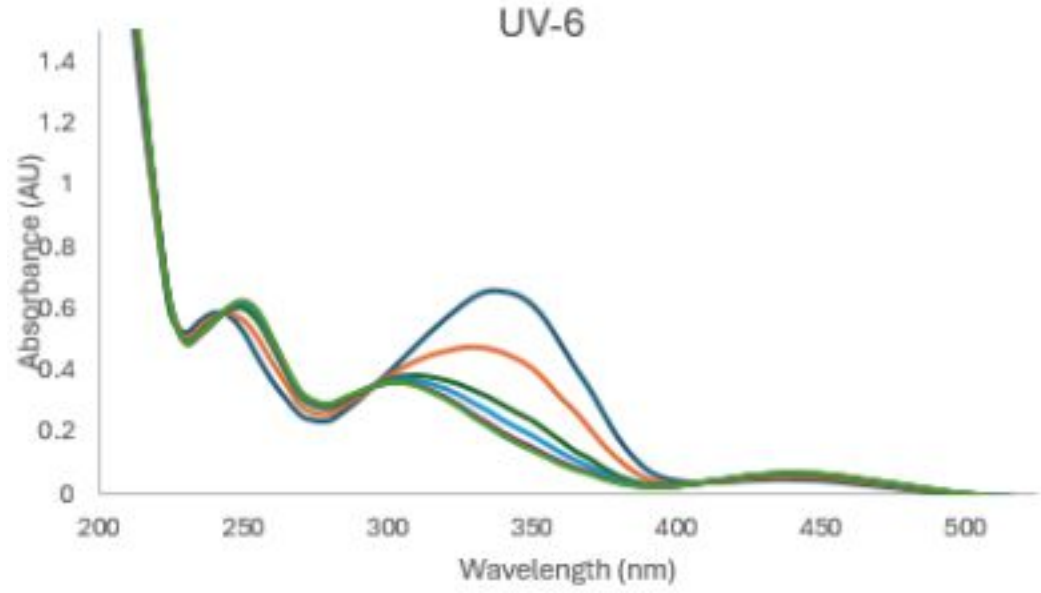

Green-6

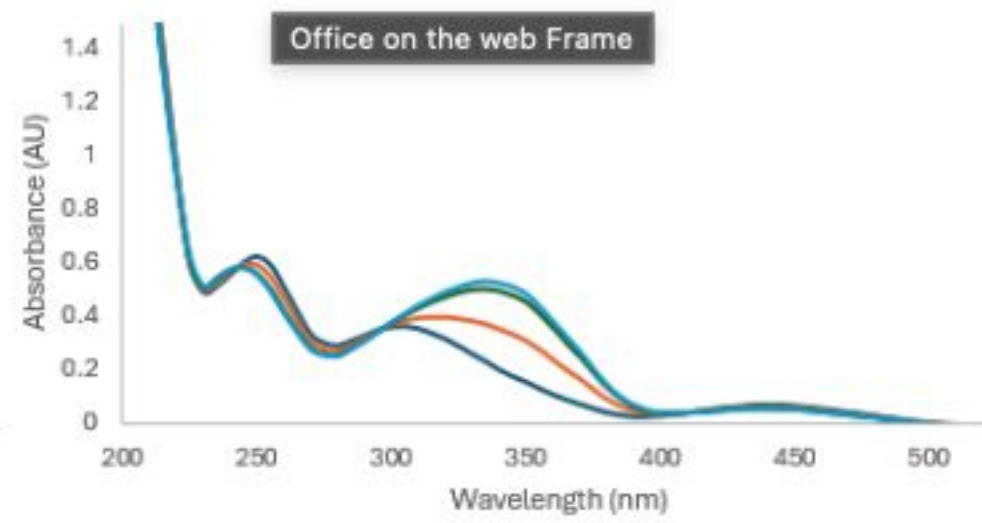

UV-3

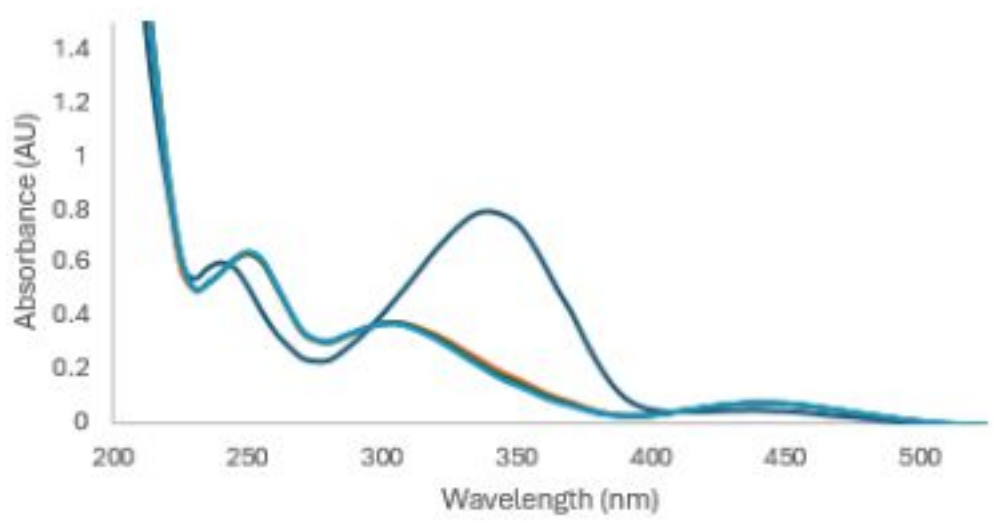

Green-3

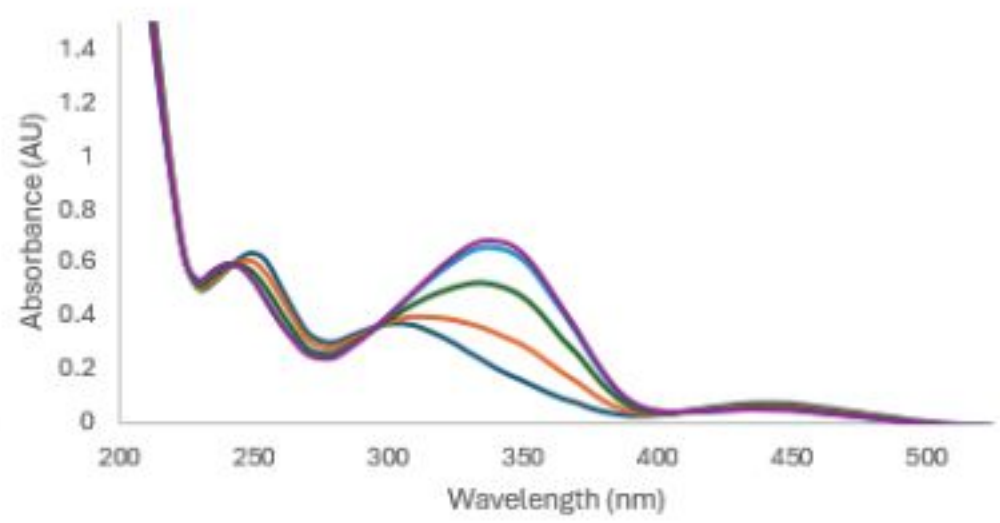

UV-4

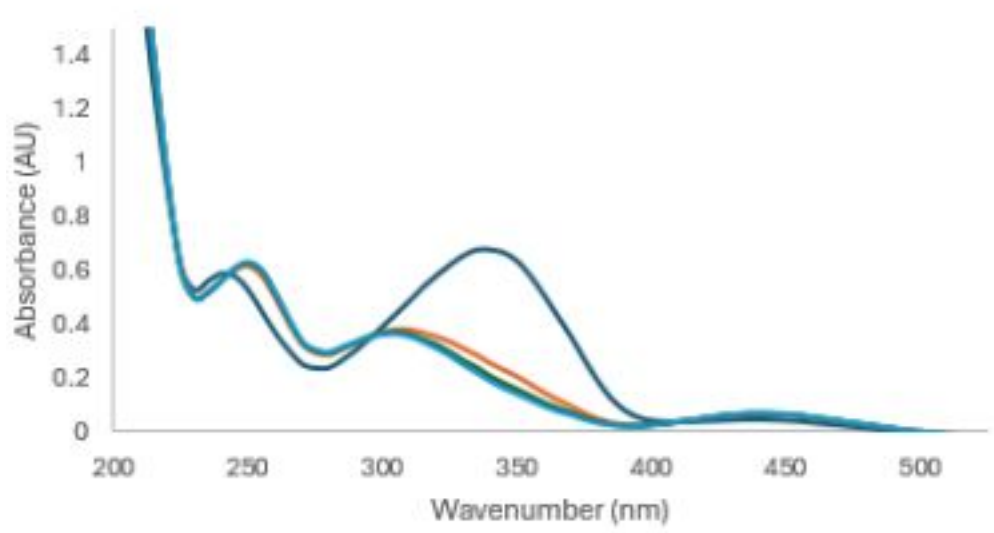

Green 4

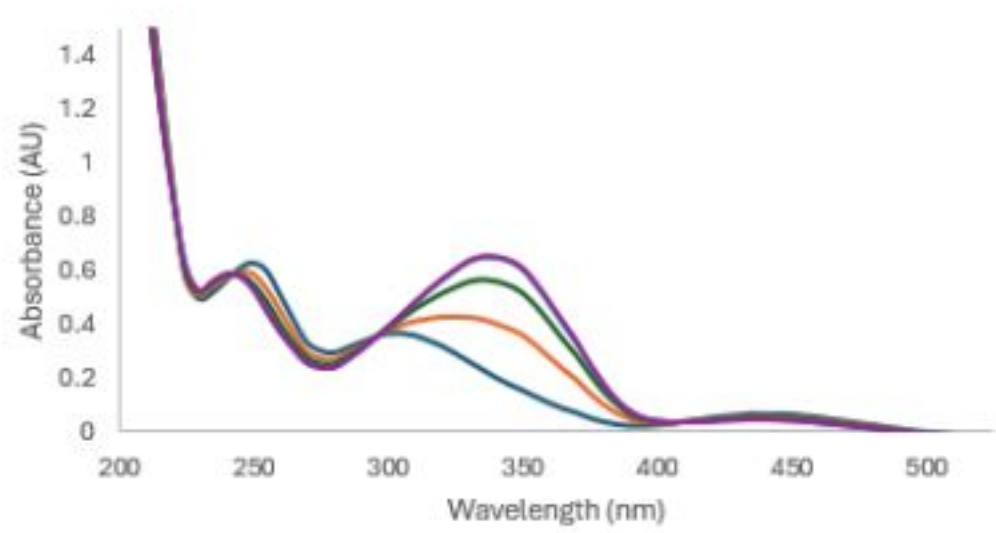

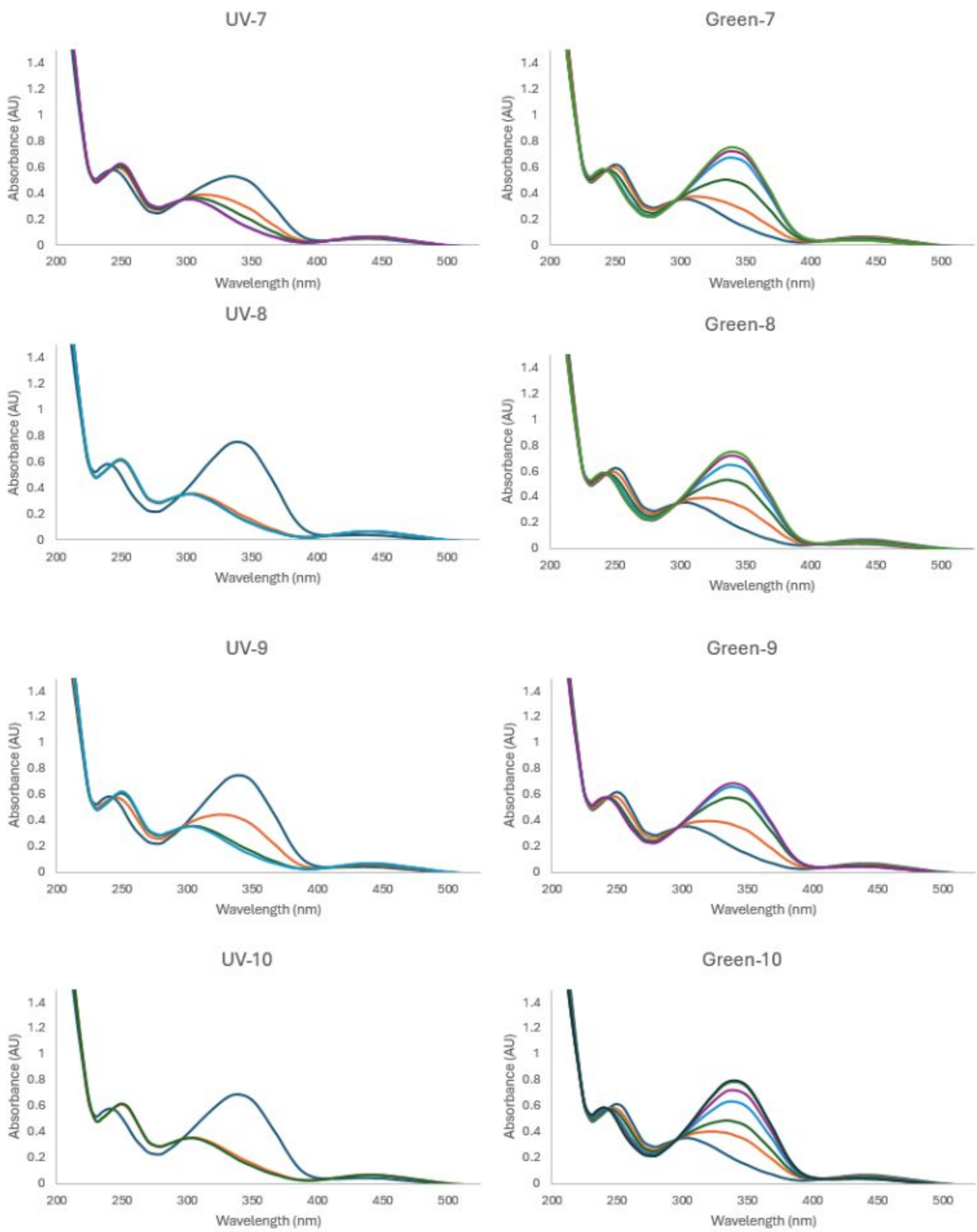
UV-7
Green-7
UV-8
Green-8
UV-9
Green-9
UV-10
Green-10
Absorbance (AU)
Wavelength (nm)
0
0.2
0.4
0.6
0.8
1
1.2
1.4
200
250
300
350
400
450
500

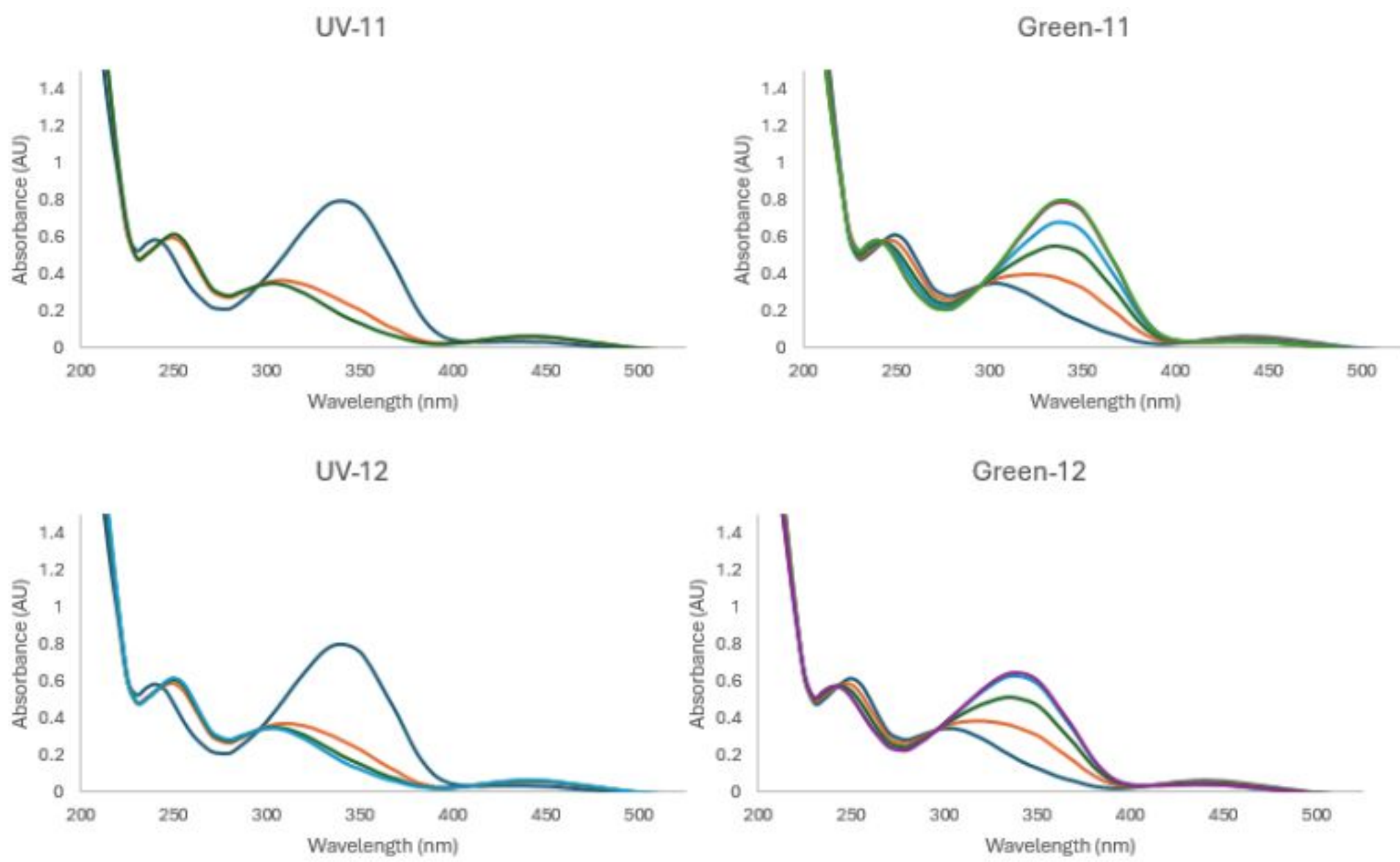

Figure S5. UV-Vis cycling study for the 1:2 DVA gel system.

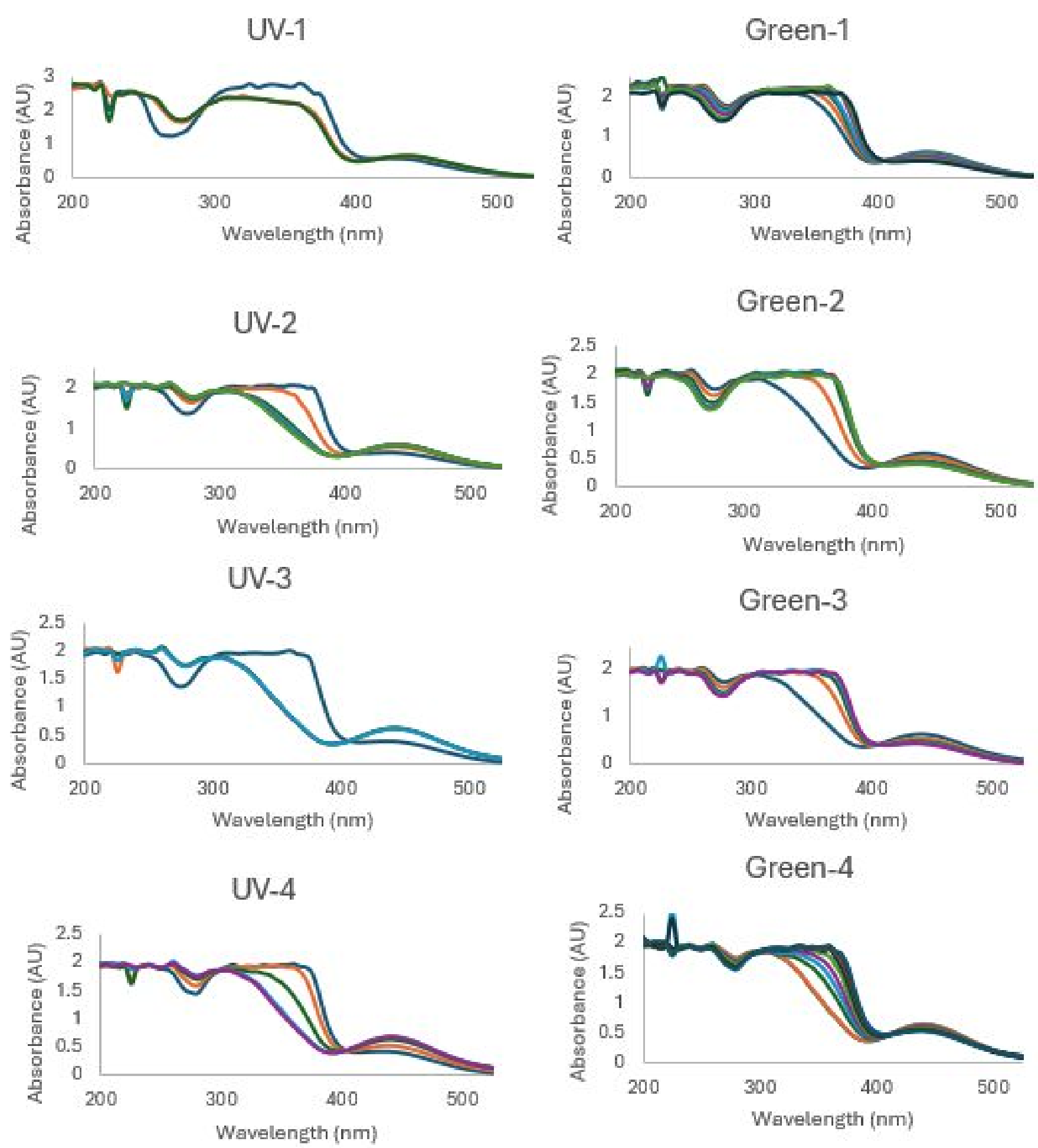

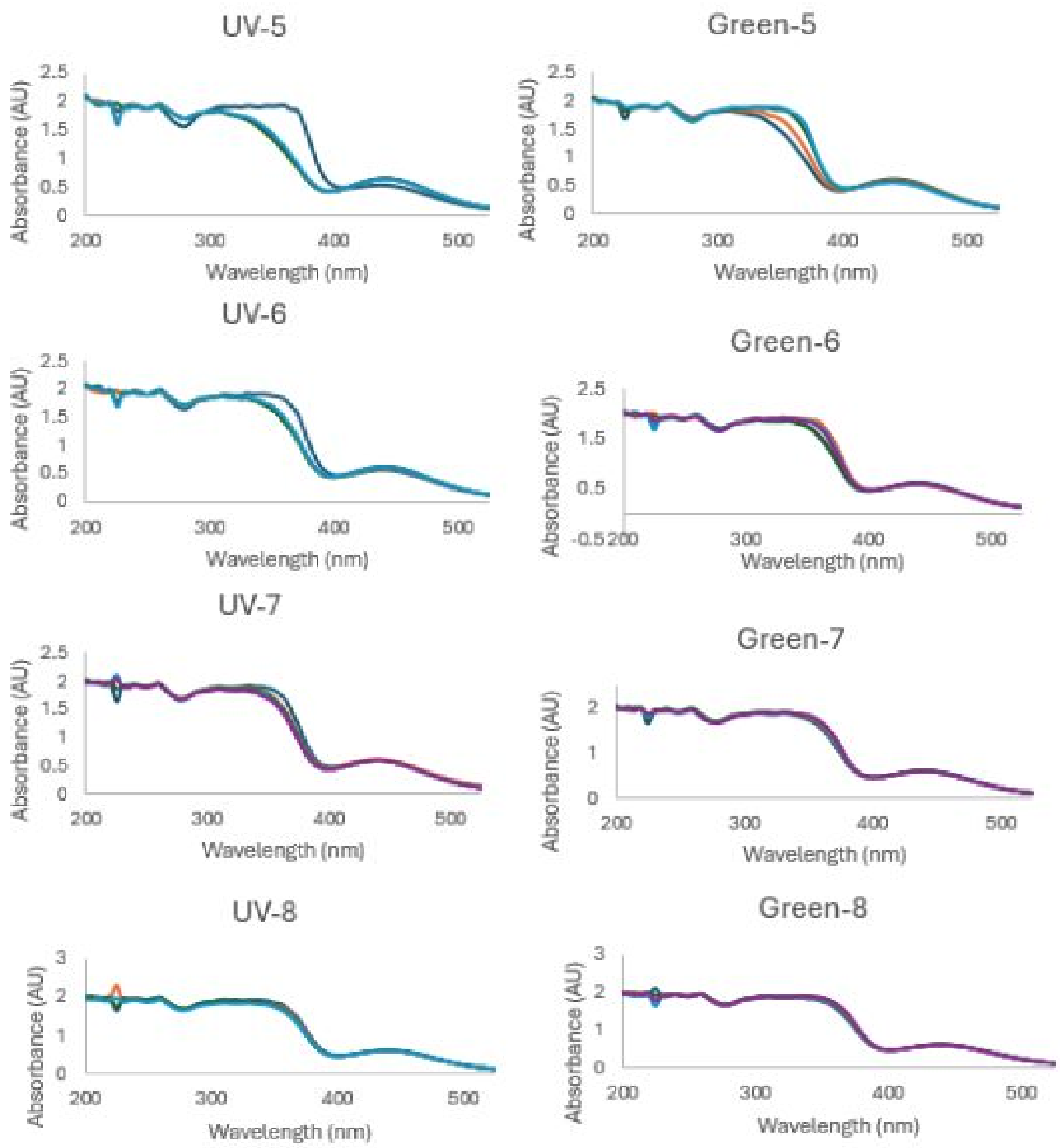

Figure S6. UV-Vis cycling study for the 1:3 DVA gel system. Note that absorption is high for the 1:3 due to the increased amount of DVA in the system at the same overall Q_8 concentration. The broader peaks are likely due to more interactions (i.e. π-π*) present between the azobenzenes themselves.

Table S1. UV-Vis cycling study for the 1:2 Vinyl gel system.

Maxima (post-green) (AU)	AVG	Minima (post-UV) (AU)	AVG
0.591501594	0.456129	0.128725782	0.078245808
0.492727399		0.072179548	
0.424933732		0.072971448	
0.412546486		0.07838881	
0.407995135		0.079390831	
0.320214003		0.077767797	
0.479426324		0.070470713	
0.471149236		0.071094215	

0.432490617		0.071508527	
0.504712224		0.072777472	
0.506653607		0.075408667	
0.429200918		0.068265885	

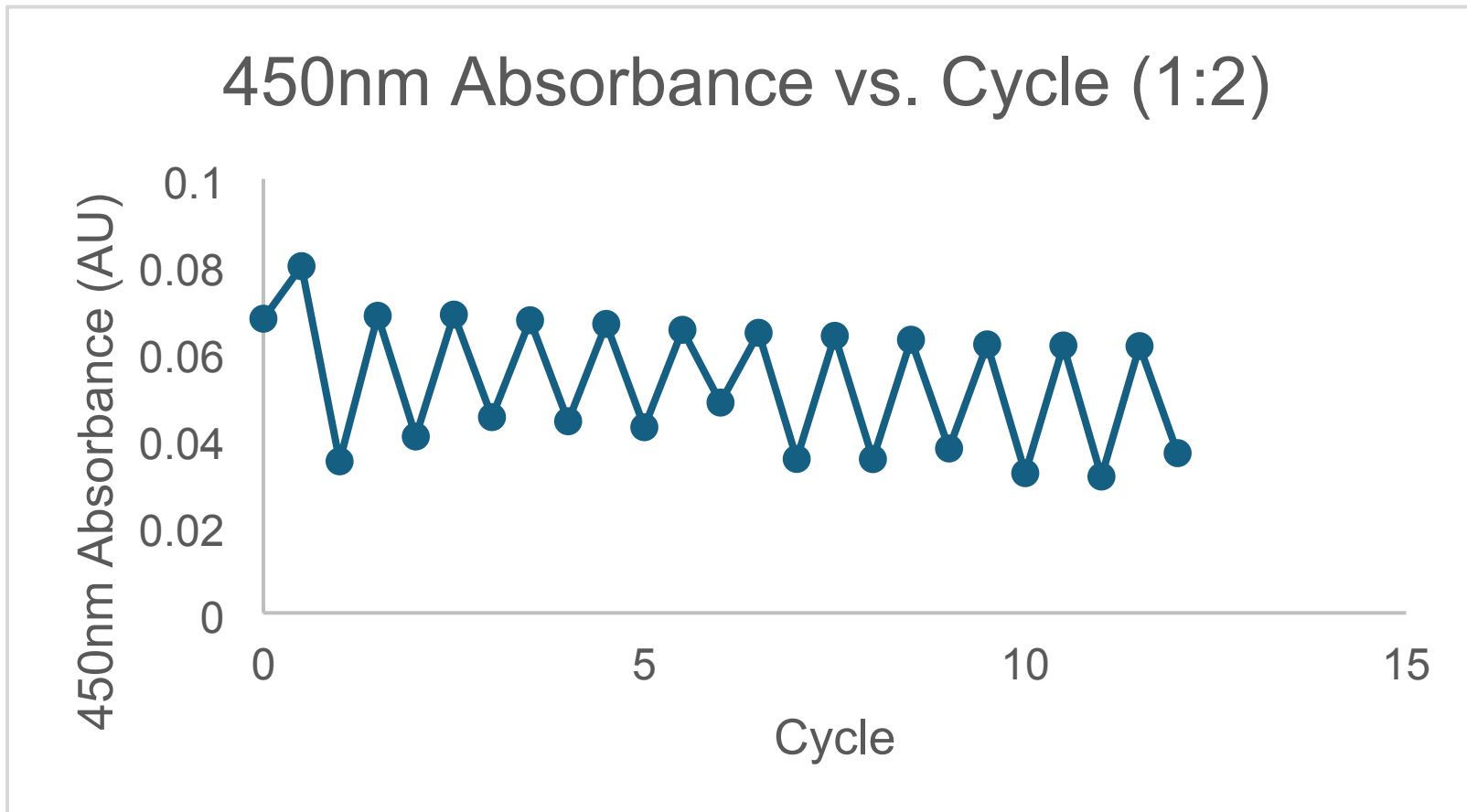

Figure S7. UV-Vis cycling study for the 1:2 DVA gel system followed at the 450 nm n-π* band.

Table S2. UV-Vis cycling study for the 1:3 DVA gel system.

Maxima (post-green) (AU)	AVG	Minima (post-UV) (AU)	AVG
0.034929693	0.038778	0.079885811	0.066045377
0.040646598		0.068455704	
0.045114912		0.06872344	
0.044154555		0.067424364	
0.042793084		0.066583298	
0.048454955		0.065222122	
0.035476472		0.064531088	
0.035439789		0.063838065	
0.037884887		0.062937744	
0.032200642		0.061865497	
0.031424593		0.061607949	
0.036812093		0.061469436	

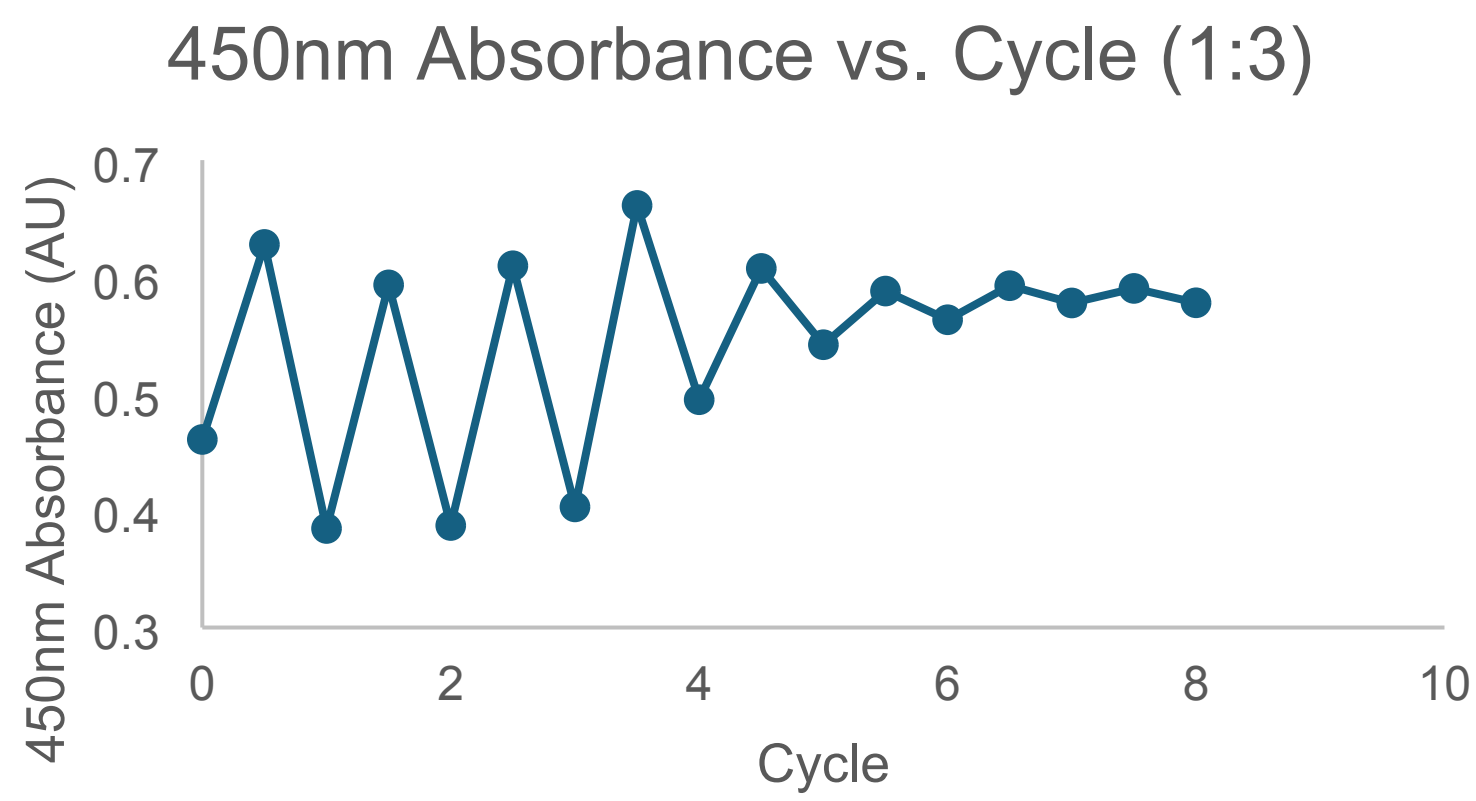

Figure S8. UV-Vis cycling study for the 1:3 DVA gel system followed at the 450 nm n-π* band.

Table S3. Effects of solvent on the surface area of each gel when allowed to relax in solution. Percentages given are based on each gels maximum in DCM.

Solvent	1:2 Allyloxy (%)	1:2 Vinyl (%)	1:3 Vinyl (%)
DCM	100	100	100.00
Toluene	92.60	42.21	99.03
Acetone	66.54	74.31	76.82
THF	97.50	75.10	95.85
Gas	75.26	44.10	-
Diesel	38.53	42.77	-
Water	-	36.81	-
Hexafluorobenzene	47.48	-	-
CFC	51.18	-	-

Table S4. Adjustments were made to each gel's initially observed surface areas by the percentages in Table 1 under the "Compared to allyloxy" column.

Solvent	1:2 Allyloxy (%)	1:2 Vinyl (%)	1:3 Vinyl (%)
DCM	100	51.90	79.88
Toluene	92.60	21.91	79.11
Acetone	66.54	38.56	61.36
THF	97.50	38.97	76.57
Gas	75.26	22.88	-
Diesel	38.53	22.19	-
Water	-	19.10	-
Hexafluorobenzene	24.64	-	-
CFC	51.18	-	-

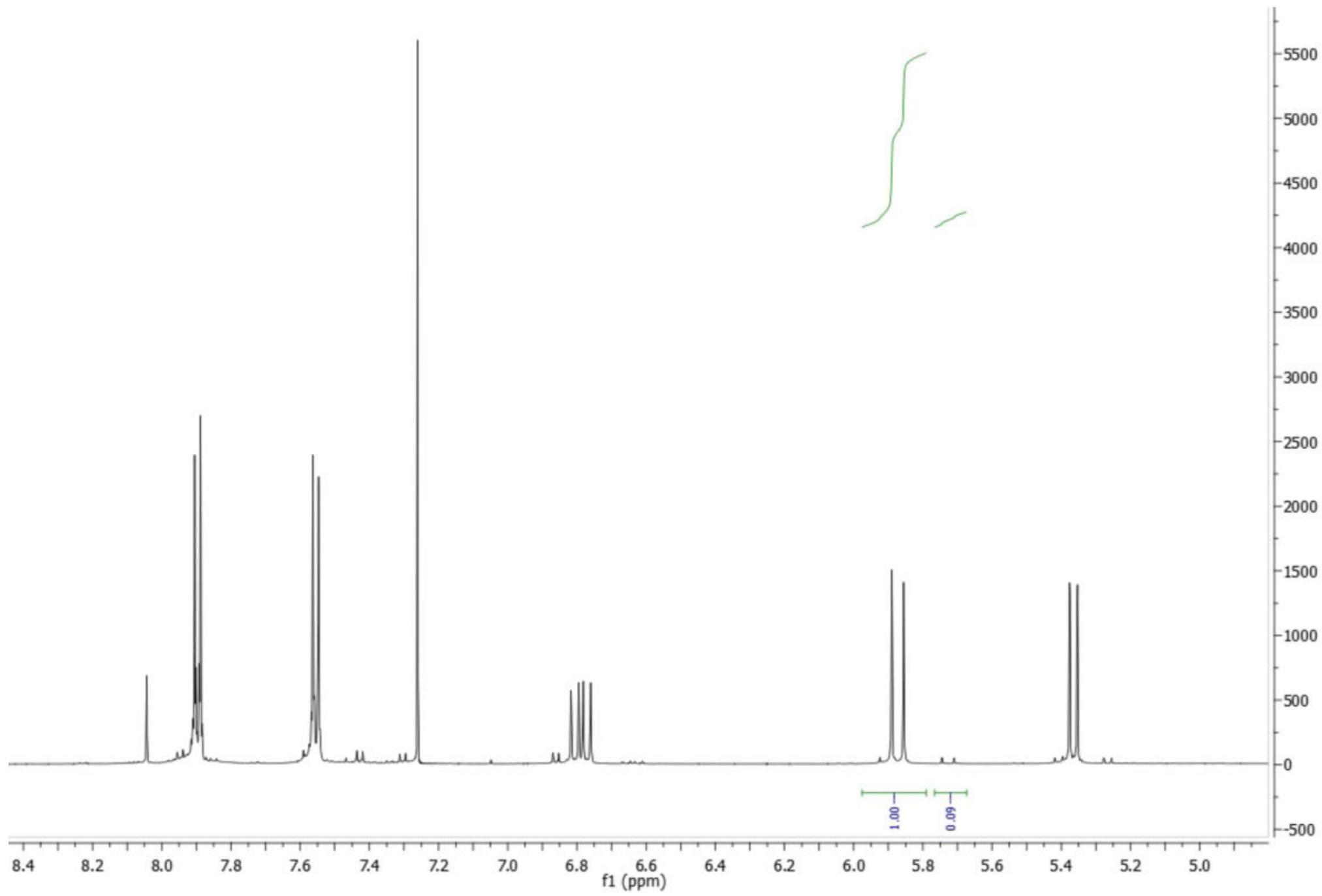

Figure S9. ^{1}H NMR spectra of 4,4'-divinylazobenzene used to estimate the Trans:Cis ratio.

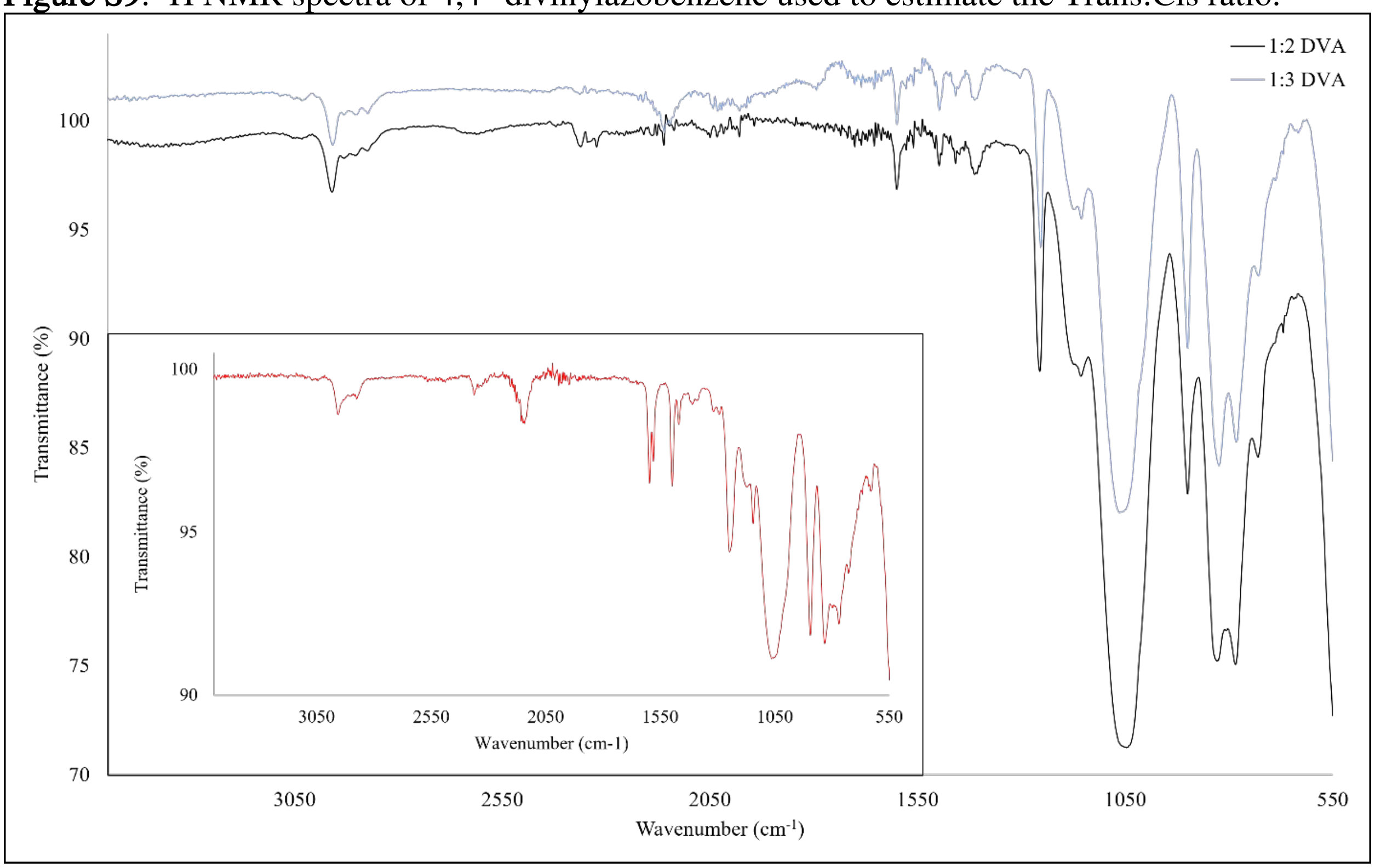

Figure S10. ATR-FTIR overlay of both DVA gel systems showing the 1:2 gel in black, 1:3 gel in blue, and an inlay of the DAA gel is given in red.

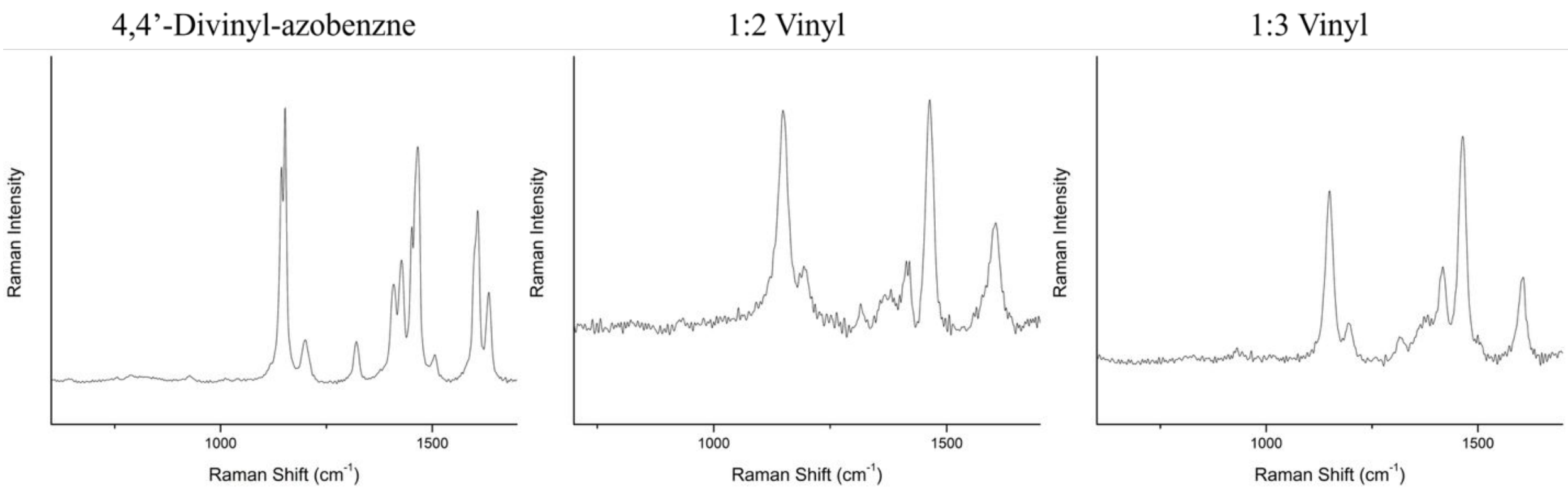

Figure S11. Raman spectra of the 4,4'-divinyl-azobenzene and both 1:2 and 1:3 vinyl gels. The vinyl peak at 1605 cm^{-1} is lost in the gels, while both the trans (1440-1450 cm^{-1}) and cis (1570-1590 cm^{-1}) N=N are present.

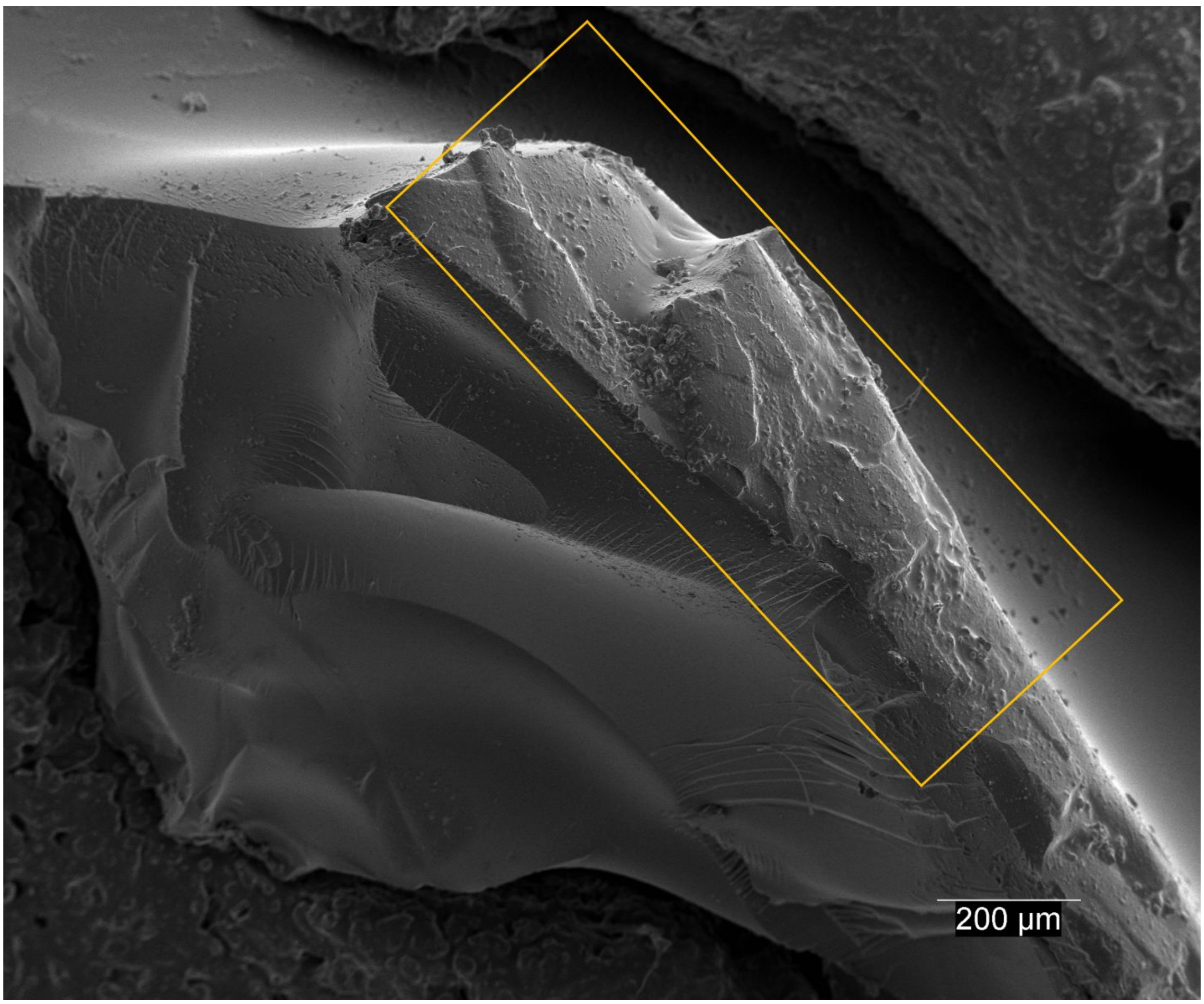

Figure S12. SEM image of the 1:2 DVA gel at 70x magnification. The yellow box outlines the coating-infused gel. No pores are observed; however, the surface is notably rougher in the region with the gel than in the surrounding area, which is composed only of the coating.

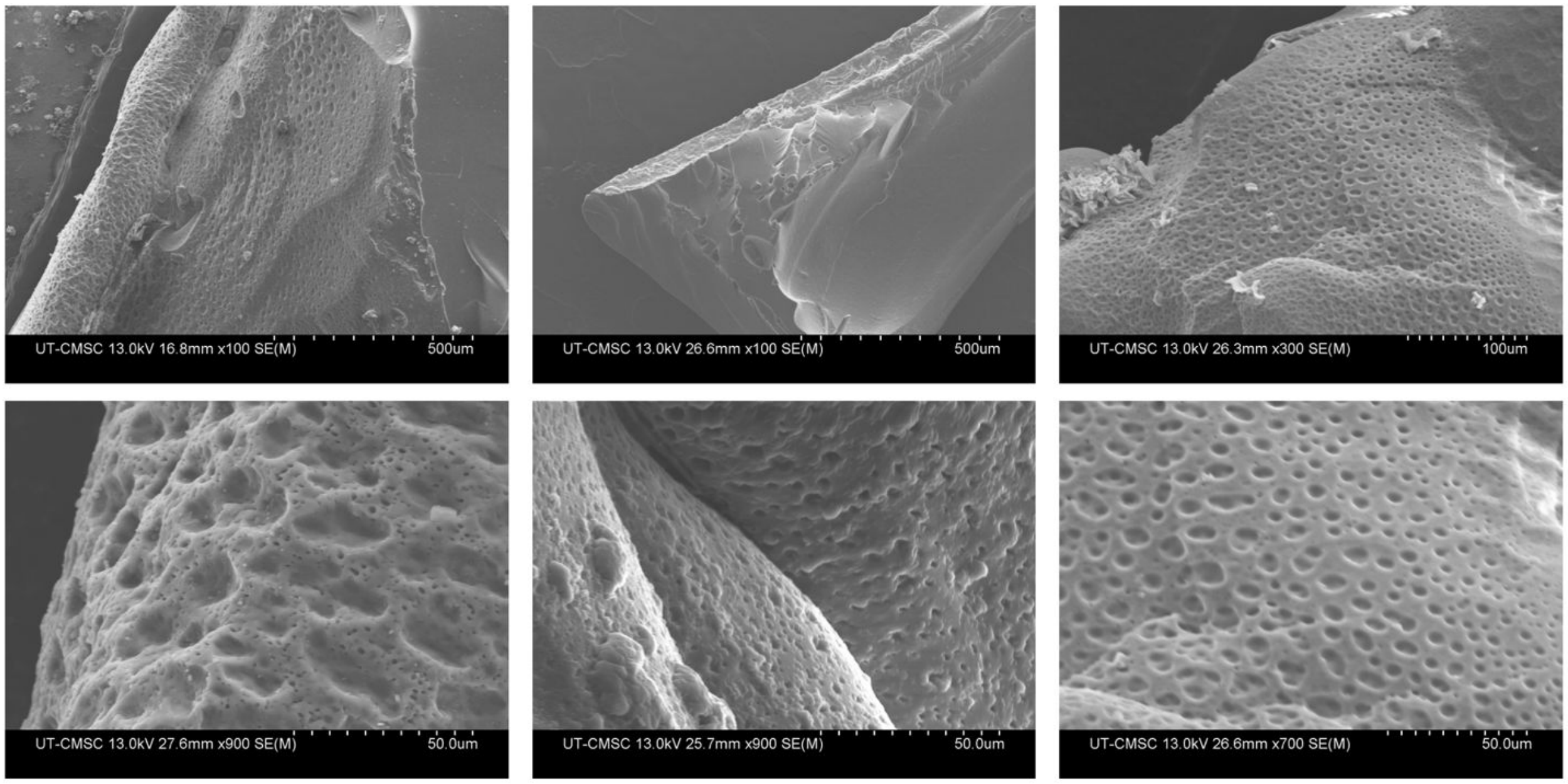

1:2 DVA – Freeze Dried | 1:3 DVA – Freeze Dried | 1:2 DAA – Freeze Dried

Figure S13. Example SEM images of freeze-dried gel materials from (DMSO). The surfaces of the particles are still mostly smooth, but the fractures show large micron-sized pockets in the samples. These may be induced by vacuum drying rather than being responsible for swellability.

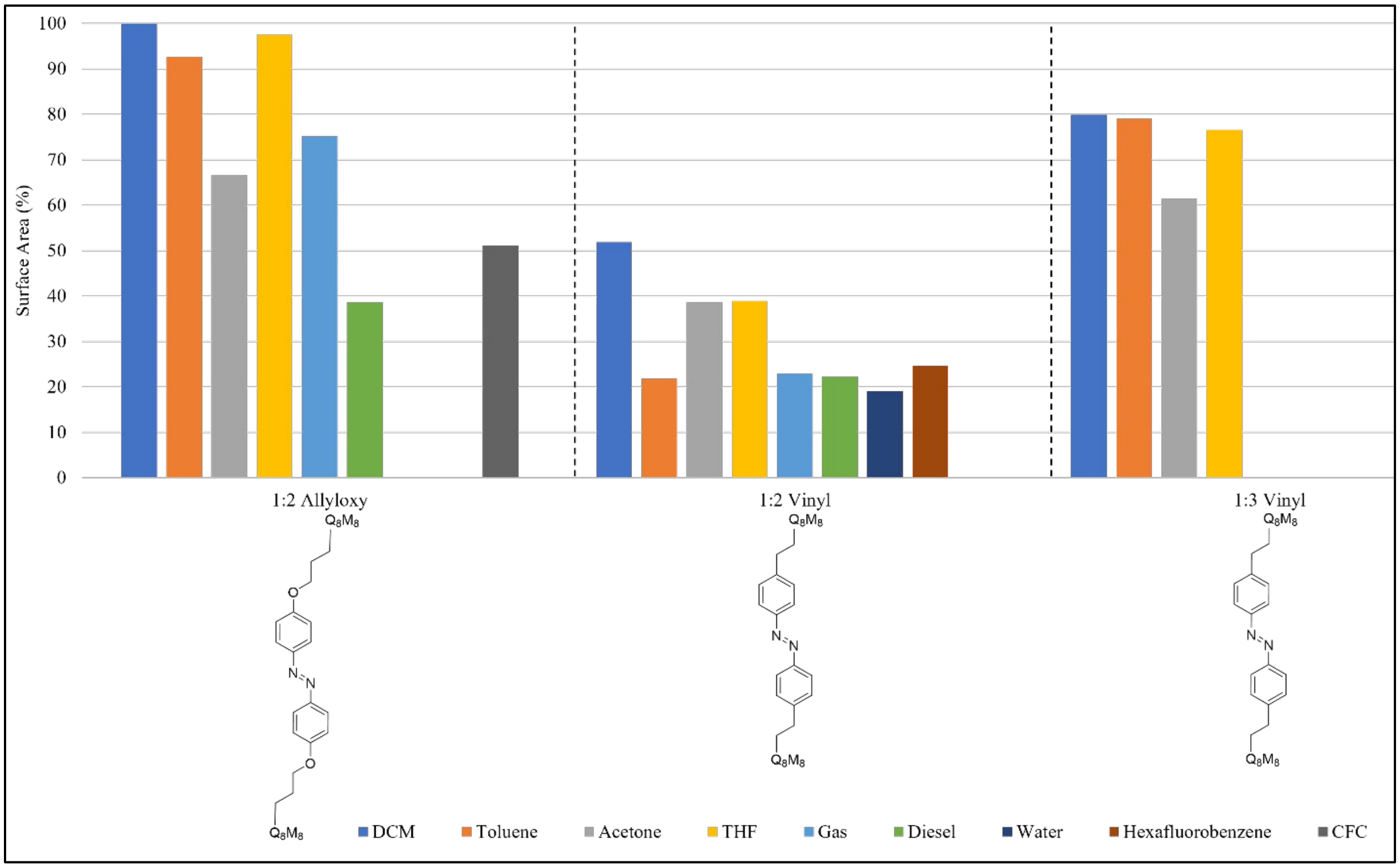

Figure S14. Ambient swelling of each material in different solvents after adjusting the swelling capacity to the proportion of the allyloxy materials, DCM swell given in Table S2.

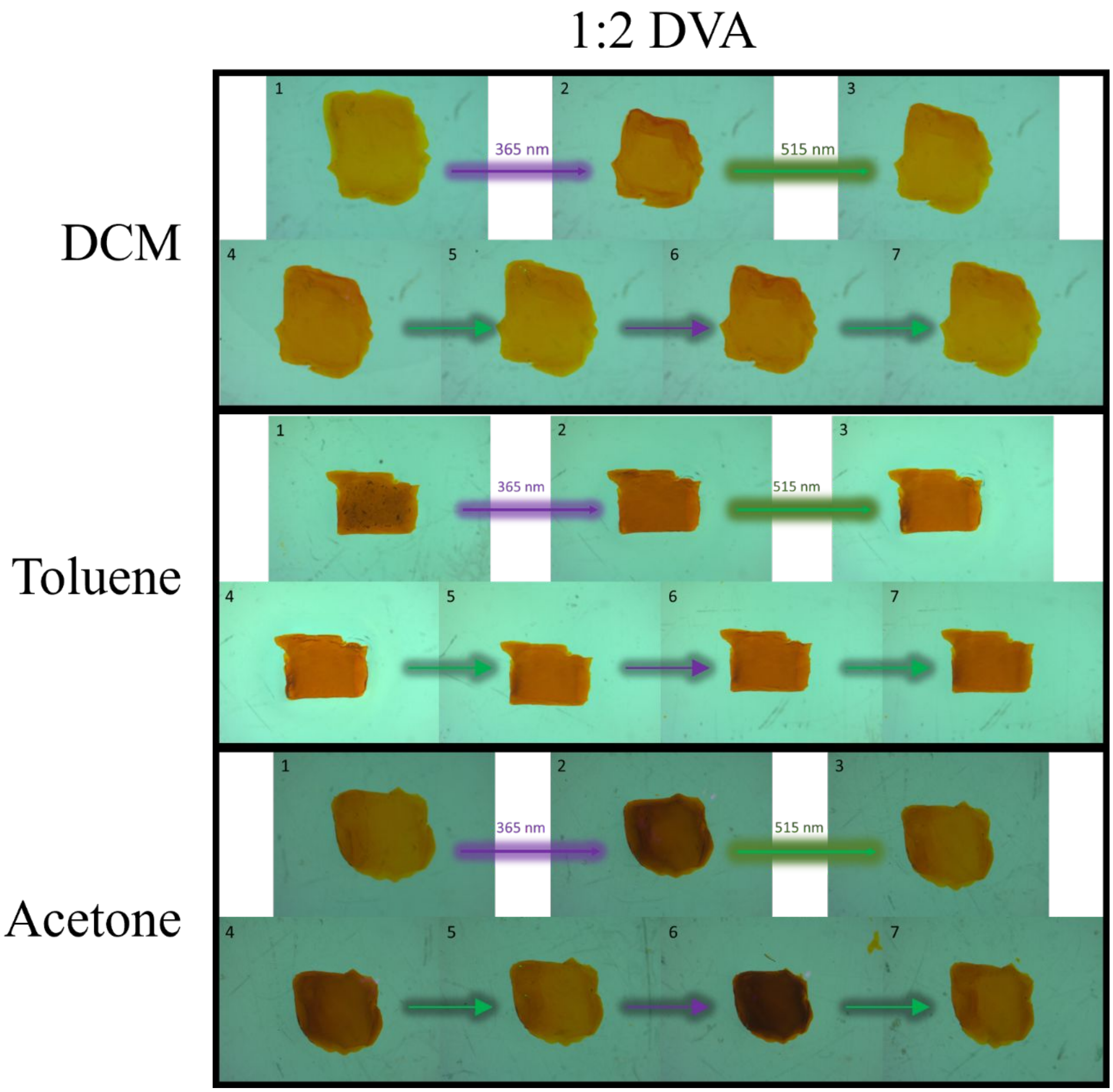

Figure S15. Actuation of the 1:2 DVA gels in DCM, toluene, and acetone by 365nm (violet) and 515nm (green) lights. In each numbered sequence, image 1 is the ambient, unactuated sample. Colored arrows indicate the irradiation between each step of the cycle.

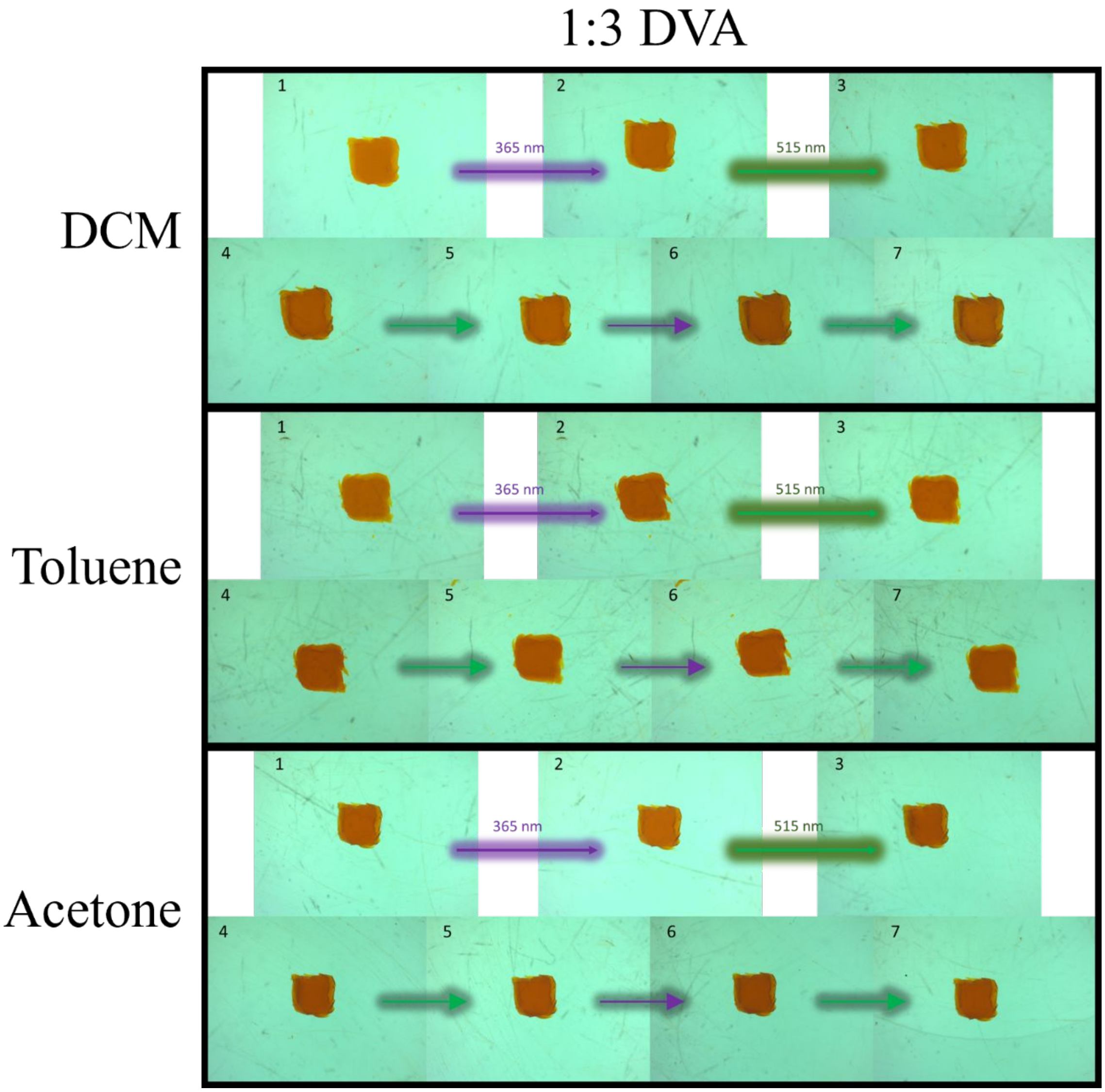

Figure S16. Actuation of the 1:3 DVA gels in DCM, toluene, and acetone by 365nm (violet) and 515nm (green) lights. In each numbered sequence, image 1 is the ambient, unactuated sample. Colored arrows indicate the irradiation between each step of the cycle.

Table S5. Table of actuation surface area measurements for each DVA gel compared to itself in DCM (All Gels formed in DCM).

Functional Group	Solvent	Initial	1st UV	1st Vis	2nd UV	2nd Vis	3rd UV	3rd Vis
1:2 Vinyl	DCM	100.00	67.65	80.59	82.63	87.93	76.36	85.21
	Toluene	42.21	42.99	43.76	44.29	41.39	40.62	41.67
	Acetone	74.31	57.97	63.46	60.33	63.70	47.38	59.16
	THF	75.10	72.67	78.00	73.35	78.89	74.53	78.51
	Hexafluorobenzene	47.48	47.72	49.10	46.80	47.72	46.18	46.99
	Diesel	42.77	39.64	39.72	39.71	40.14	40.14	40.41
	Gasoline	44.10	43.35	43.05	43.64	43.12	43.12	44.00
	Water	36.81	37.25	37.30	37.50	37.44	37.55	37.55
1:3 Vinyl	DCM	100.00	103.07	98.86	108.05	101.71	107.34	104.45
	Toluene	99.03	97.77	97.62	96.75	96.99	95.14	97.50
	Acetone	76.82	75.11	78.96	73.58	73.06	72.20	71.98
	THF	95.85	97.45	102.24	100.63	100.45	101.47	100.61

Table S6. Table of actuation surface area measurements for each DVA gel compared to the maximum swell of the DAA gel in DCM.

Functional Group	Solvent	Initial	1st UV	1st Vis	2nd UV	2nd Vis	3rd UV	3rd Vis
1:2 Vinyl	DCM	51.90	35.11	41.82	42.88	45.63	39.63	44.22
	Toluene	21.91	22.31	22.71	22.98	21.48	21.08	21.62
	Acetone	38.56	30.08	32.93	31.31	33.06	24.59	30.70
	THF	38.97	37.71	40.48	38.07	40.94	38.68	40.74
	Hexafluorobenzene	24.64	24.77	25.48	24.29	24.76	23.96	24.39
	Diesel	22.19	20.57	20.61	20.61	20.83	20.83	20.97
	Gasoline	22.88	22.50	22.34	22.65	22.38	22.38	22.83
	Water	19.10	19.33	19.36	19.46	19.43	19.49	19.49
1:3 Vinyl	DCM	79.88	82.34	78.97	86.32	81.25	85.74	83.44
	Toluene	79.11	78.11	77.98	77.28	77.48	76.00	77.89
	Acetone	61.36	60.00	63.08	58.77	58.36	57.67	57.50
	THF	76.57	77.85	81.67	80.38	80.24	81.06	80.37

Ultra Small Angle Neutron Scattering:

USANS measurements were conducted on the BL-1A USANS instrument at the Spallation Neutron Source,[1,2] using irradiation at three wavelengths (1.2, 1.8, and 3.6 Å) to cover the q range from 5×10^{-5} to 3×10^{-3} Å^{-1} at ~8 hr per sample run times. The samples were loaded into titanium cells with quartz windows containing a 2 mm spacer. Gel samples were made in situ with a sol solution in either deuterated DCM or toluene being added to the sample cell, followed by the addition of Karstedt's catalyst solution and mixing with a pipette to obtain a homogeneous gel in ~10 min. Samples were left to stand for an additional 45 min before measurement to ensure complete reaction. An empty blank sample cell was measured to facilitate data reduction and background correction. In-situ photon irradiation was set up as shown in Figure S11 with a fiber optic cable and photon fluxes ~20 mW/cm^2 (365 and 515 nm). Samples were measured in their

static native conformation, then under UV irradiation, and, in some cases, back to visible light. Light irradiation was conducted for 30 min before neutron irradiation and continued throughout the neutron scan. The obtained data were corrected for an empty-cell background and presented in absolute intensity units (Figure S12).

Guinier–Porod (as well as Guinier and Power Law) fits were performed in Igor using the Irena package developed at the Advanced Photon Source, and in sasmodels at ORNL.[3] However, BGSU and ORNL scientists could not determine rational fits to the data, suggesting that the data were too noisy to accurately capture structural change information.

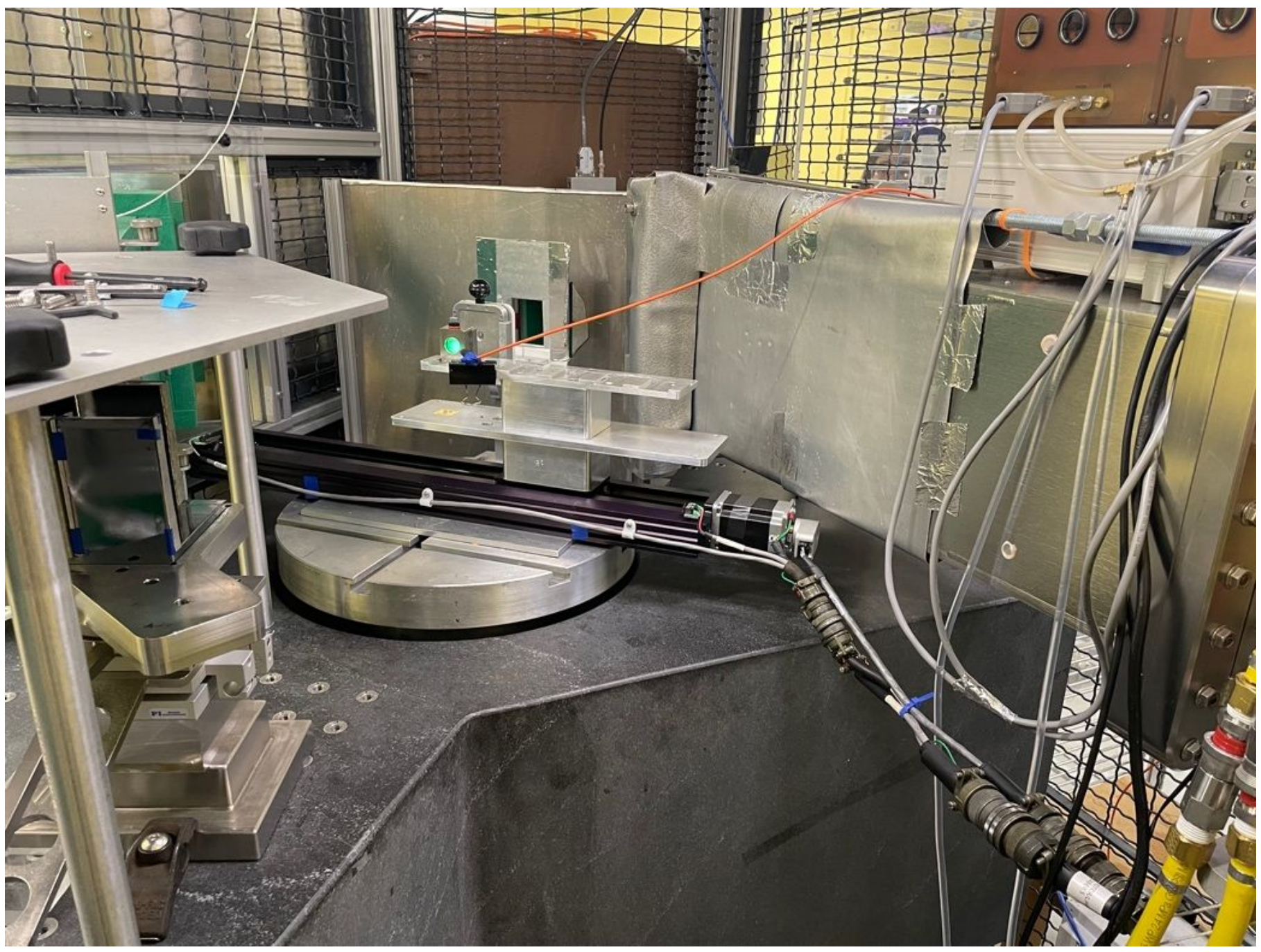

Figure S17. USANS sample environment for in-situ light irradiation studies (UV 365 nm, and Visible 515 nm).

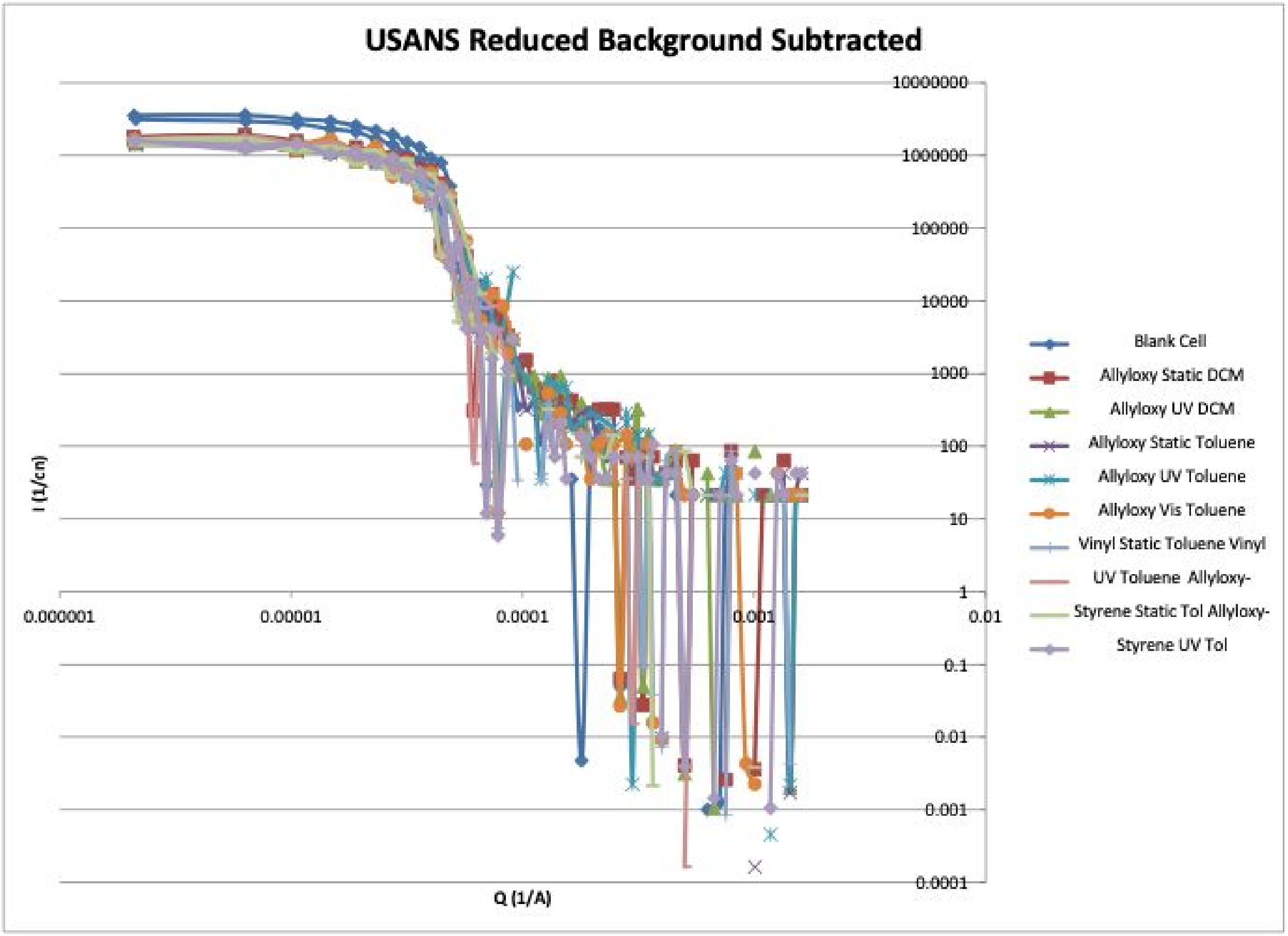

Figure S18. USANS reduced and background subtracted q-data for measured samples 1:2 Allyloxy Azo (DAA), 1:2 Vinyl Azo (DVA), 1:2:4 Q_8: Allyloxy Azo: Styrene under UV and Visible/Static conditions.

Small Angle Laser Light Scattering (SALS): SALS was conducted in the Morozova lab at Case Western Reserve University using methods outlined in the manuscript by Dhakal et al.[4] for measurement and fitting. Gel samples were prepared from a sol solution of Q-cage and azobenzene (divinyl or diallyloxy) in DCM. Karstedt's catalyst in xylenes (~1 μL) was added to the quartz sample holder, sealed, and allowed to gel for 10 min with gentle warming in the hands. A separate stand was used to mount a UV 365 nm LED irradiation source (Thorlabs), which was directed at the sample during measurement. Light scattering was performed on samples both before and after UV irradiation to observe structural changes (Figure S12). While no material shows a clear trend between ambient and irradiated conditions, there is a notable difference between the DAA and DVA systems. This suggests that the shorter crosslinker results in smaller pores overall.

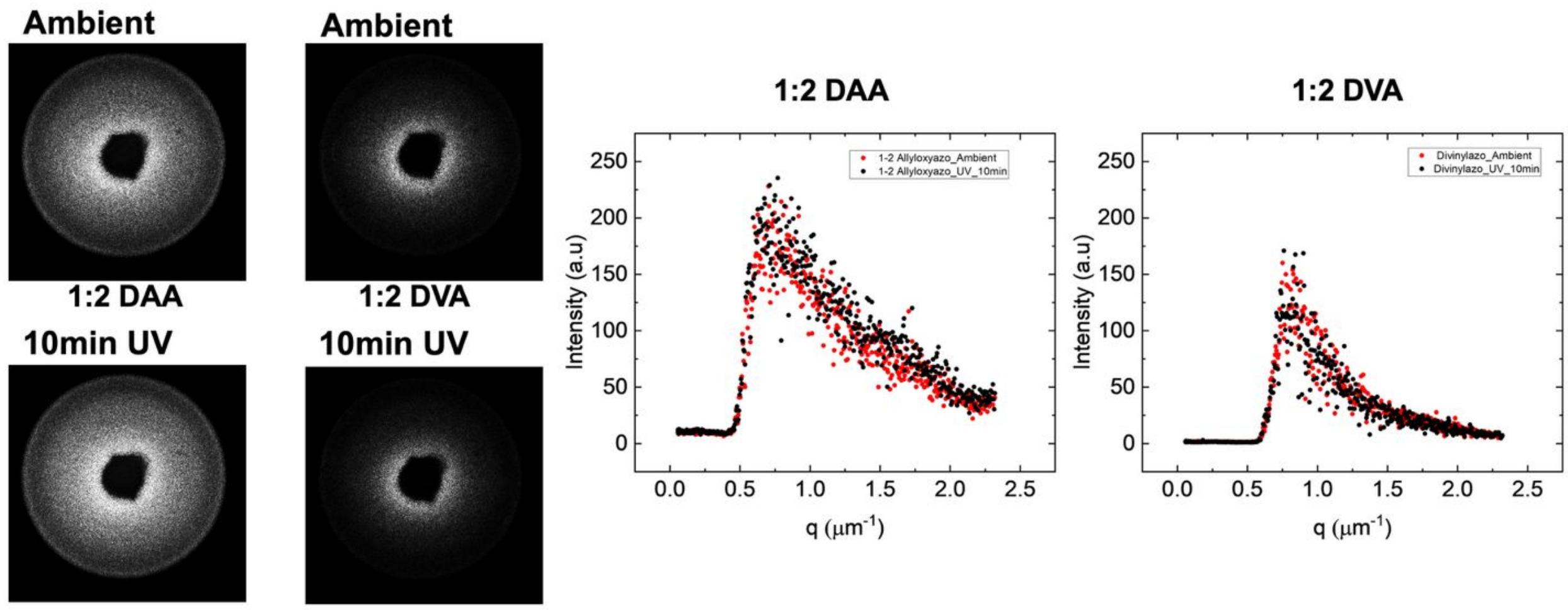

Figure S19. SALS light scattering images and q-plots of two different azobenzene-bridged Q-cage gel networks, all at 1:2 cage to azobenzene ratios.

Notes: No significant changes were observable between irradiated and non-irradiated samples, suggesting size changes are outside of the q-range (100 nm to 2 μm features) or sample absorption issues limiting scattering intensity. Differences in scatter intensity could relate to structural feature size differences, but are more likely due to differences in sample absorption, which made these samples challenging to analyze.

<u>Small Angle X-ray Scattering (SAXS): Discussion and methods in the manuscript.</u>

Abbreviations
Rg: Radius of gyration
I(0): Xray scattering intensity (arbitrary units)
Dmax: maximum intramolecular distance within a particle – pair distribution function P(r) drops to zero
$\mathbf{V_p}$: Porod Volume (excluded volume in the swollen structure)
Guinier: Linear analysis at the low q region of the SAXS data
GNOM: A program within the ATSAS SAS data analysis software for the indirect Fourier transform of scattering data to calculate the pair-distance distribution function P(r) that takes into account fits in the low and high q regions of data.
$\boldsymbol{\chi^2}$(fit): Closer to 1, the better the data fits the GNOM Indirect Fourier Transform (IFT) model
$\mathbf{R^2}$(fit): Guinier fit – typical R^2 linear fit.

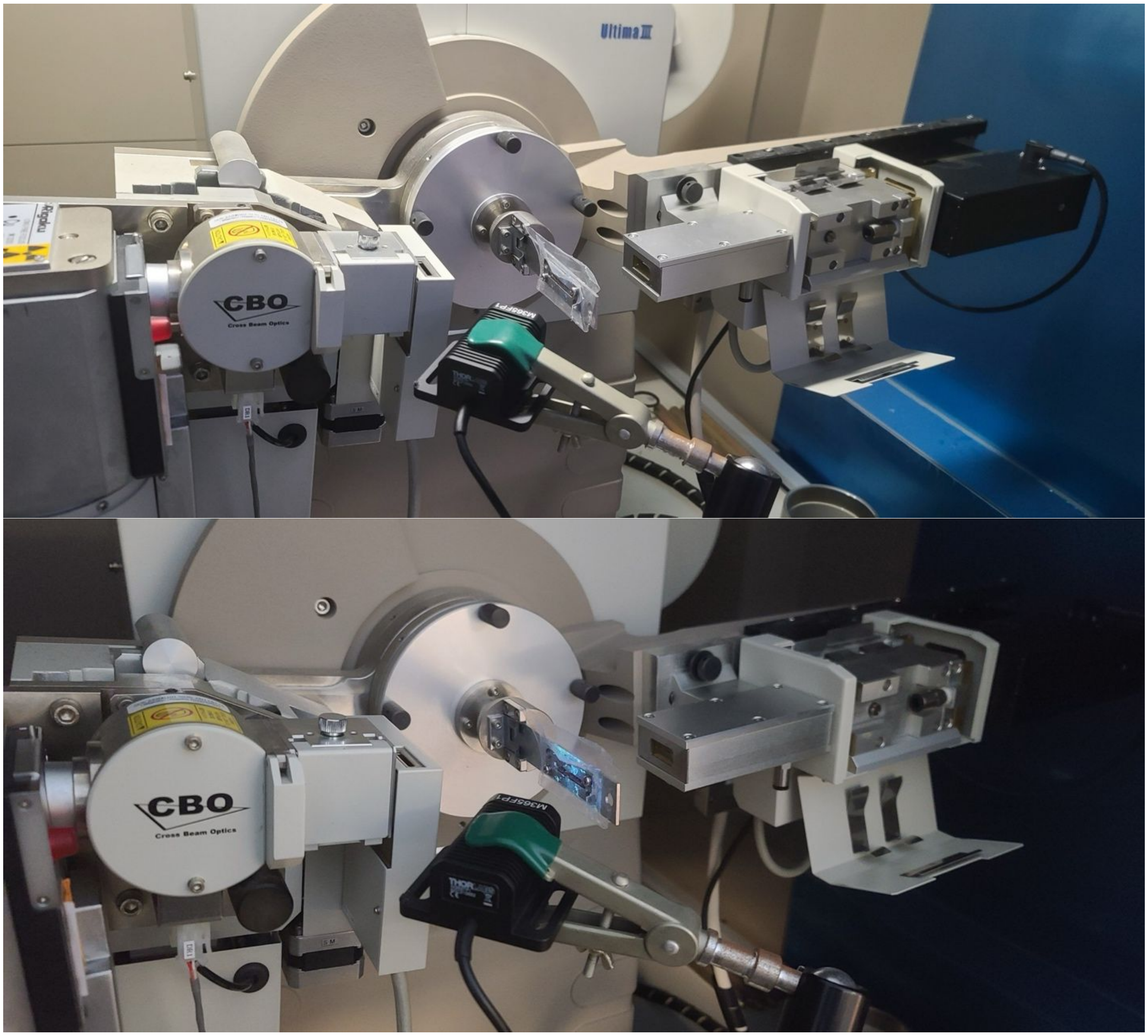

Figure S20. SAXS transmission setup within the Rigaku Ultima III showing non-irradiated (upper) and UV-irradiated (lower) sample positioning.

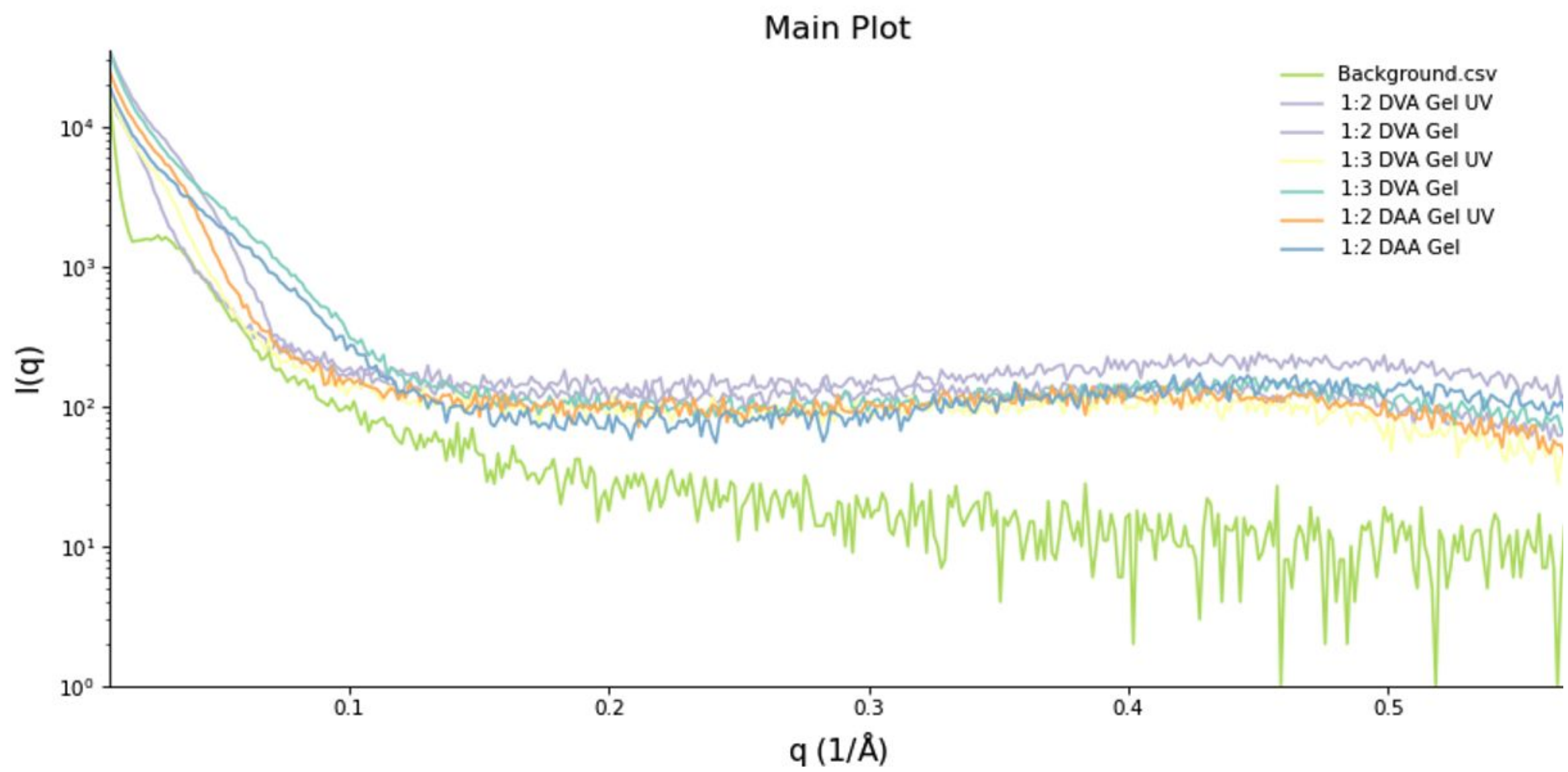

Figure S21. Raw scattering log intensity data from gels and background (Mylar) samples.

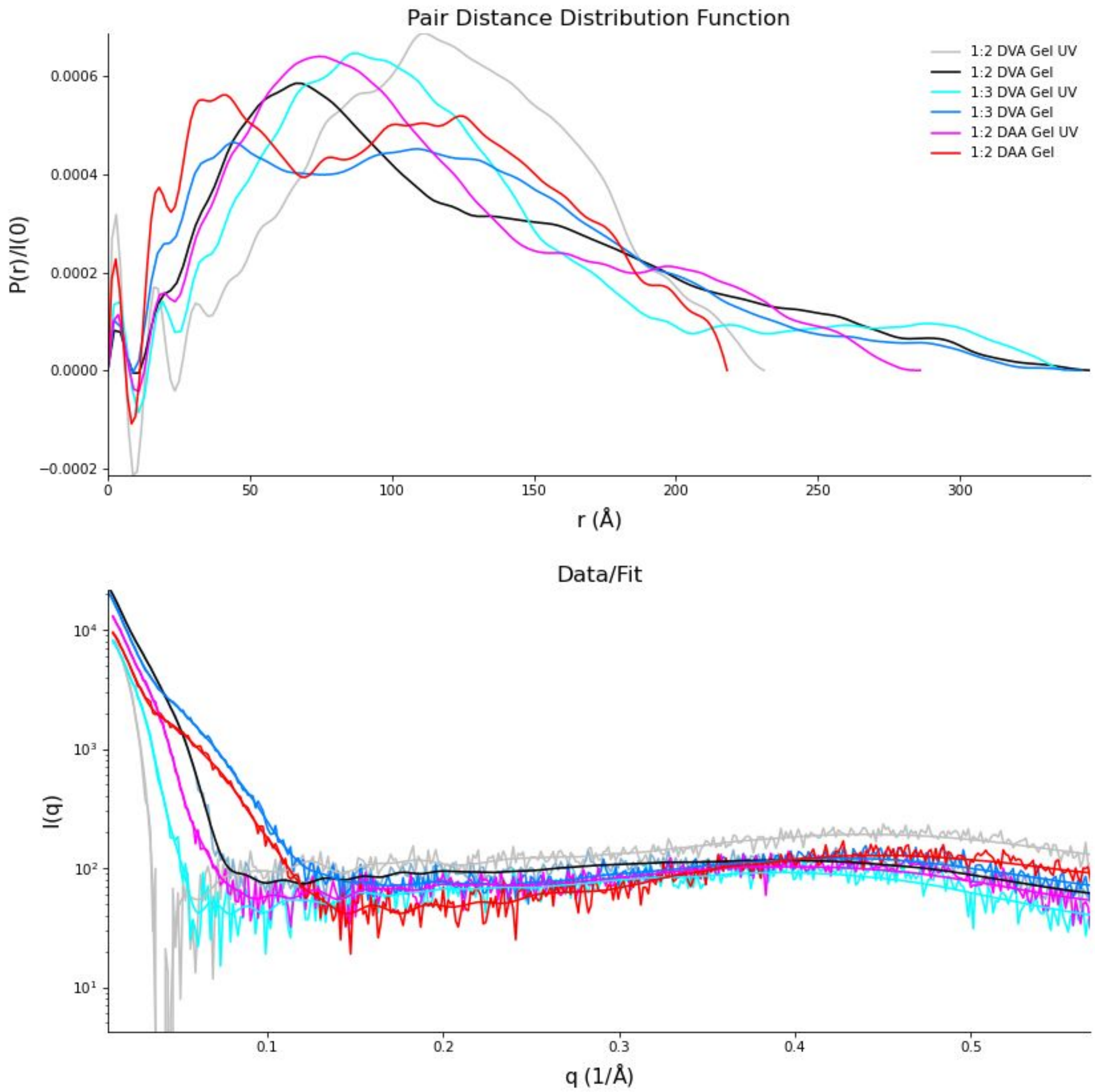

Figure S22. GNOM Pair Distance Distribution Functions and data analysis fits for scattering data.

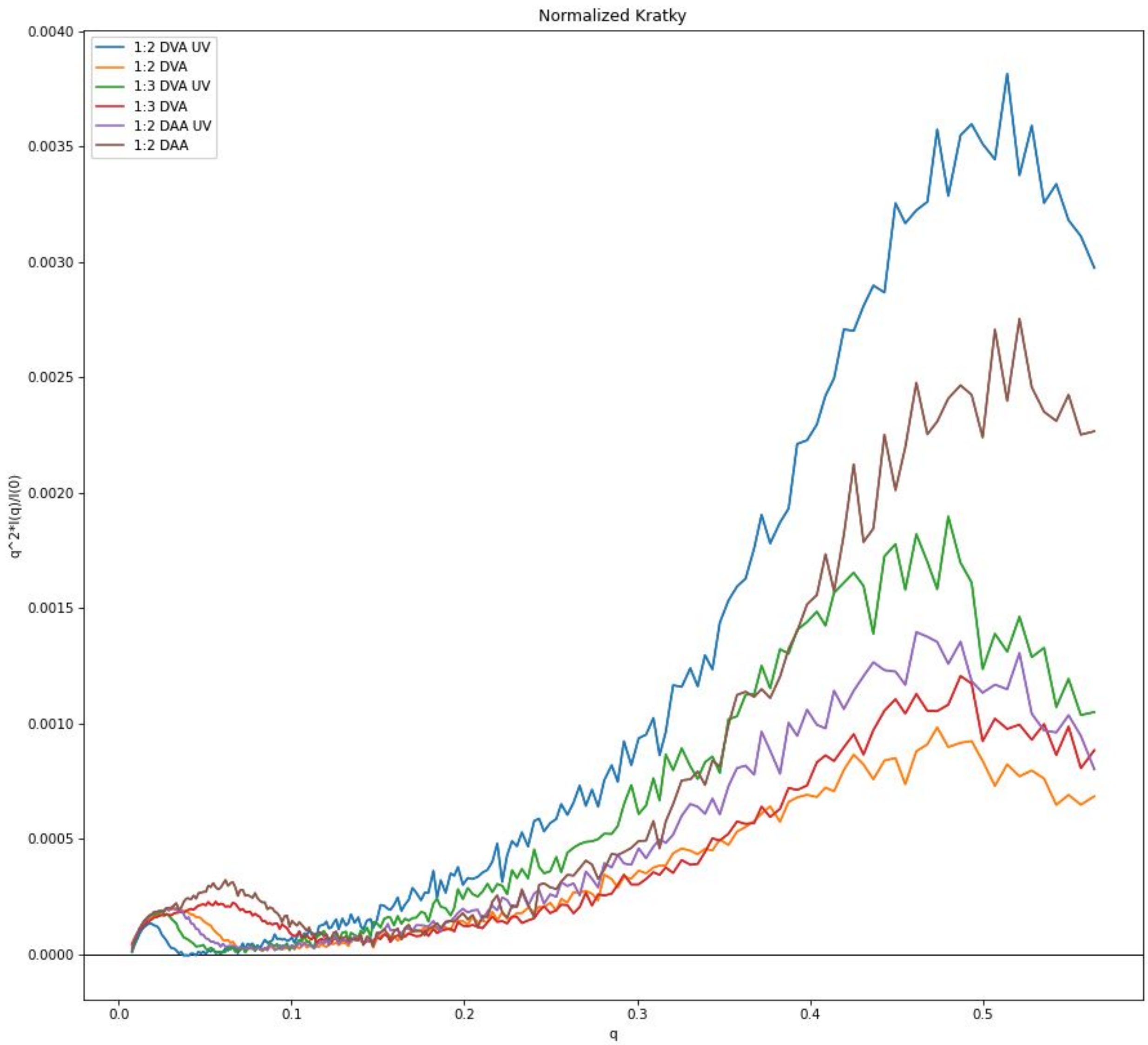

Figure S23. Normalized Kratky analysis versus q (Å^{-1}).

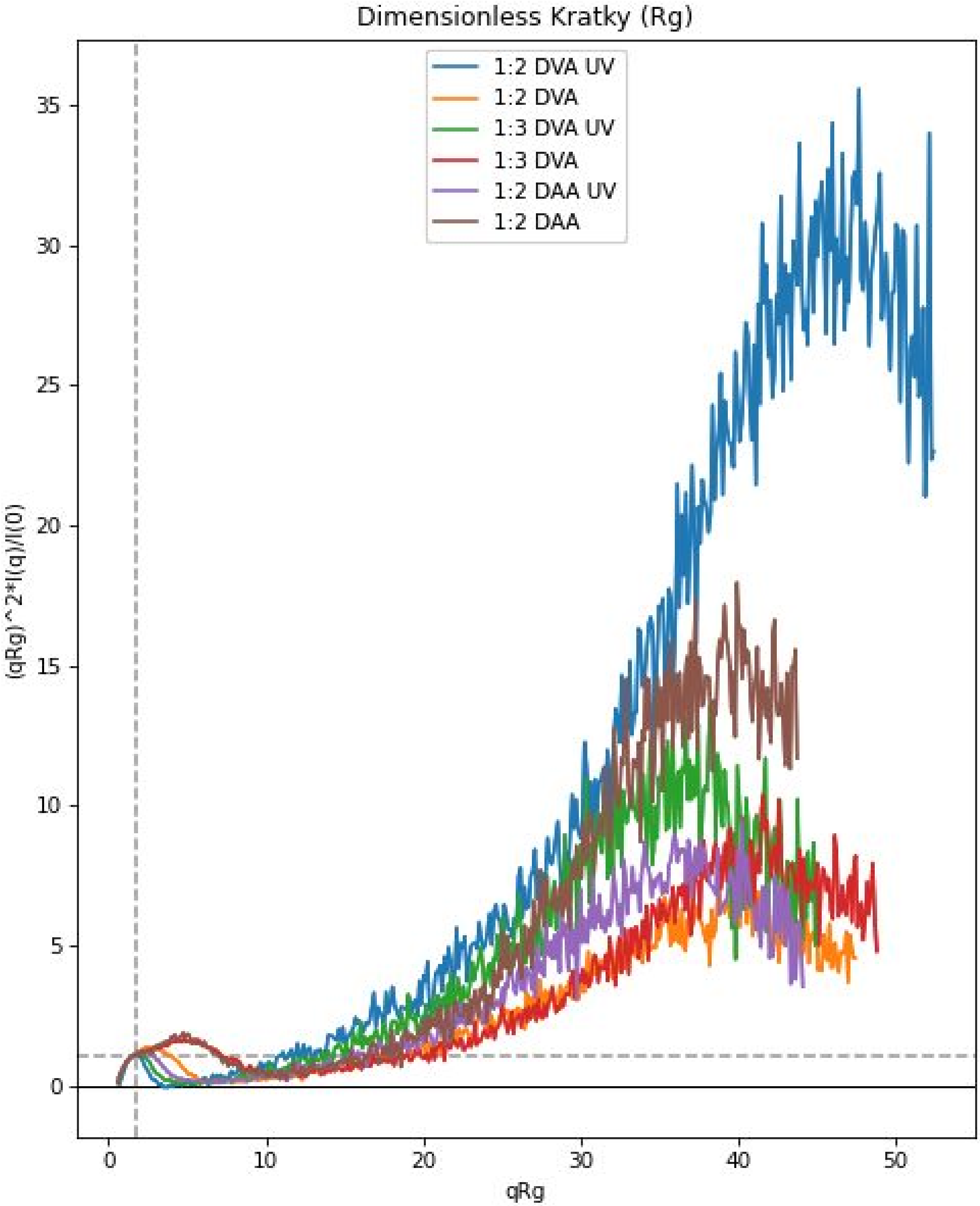

Figure S24. Normalized Kratky analysis versus qRg (Å^{-1}), which normalizes to the Rg values.

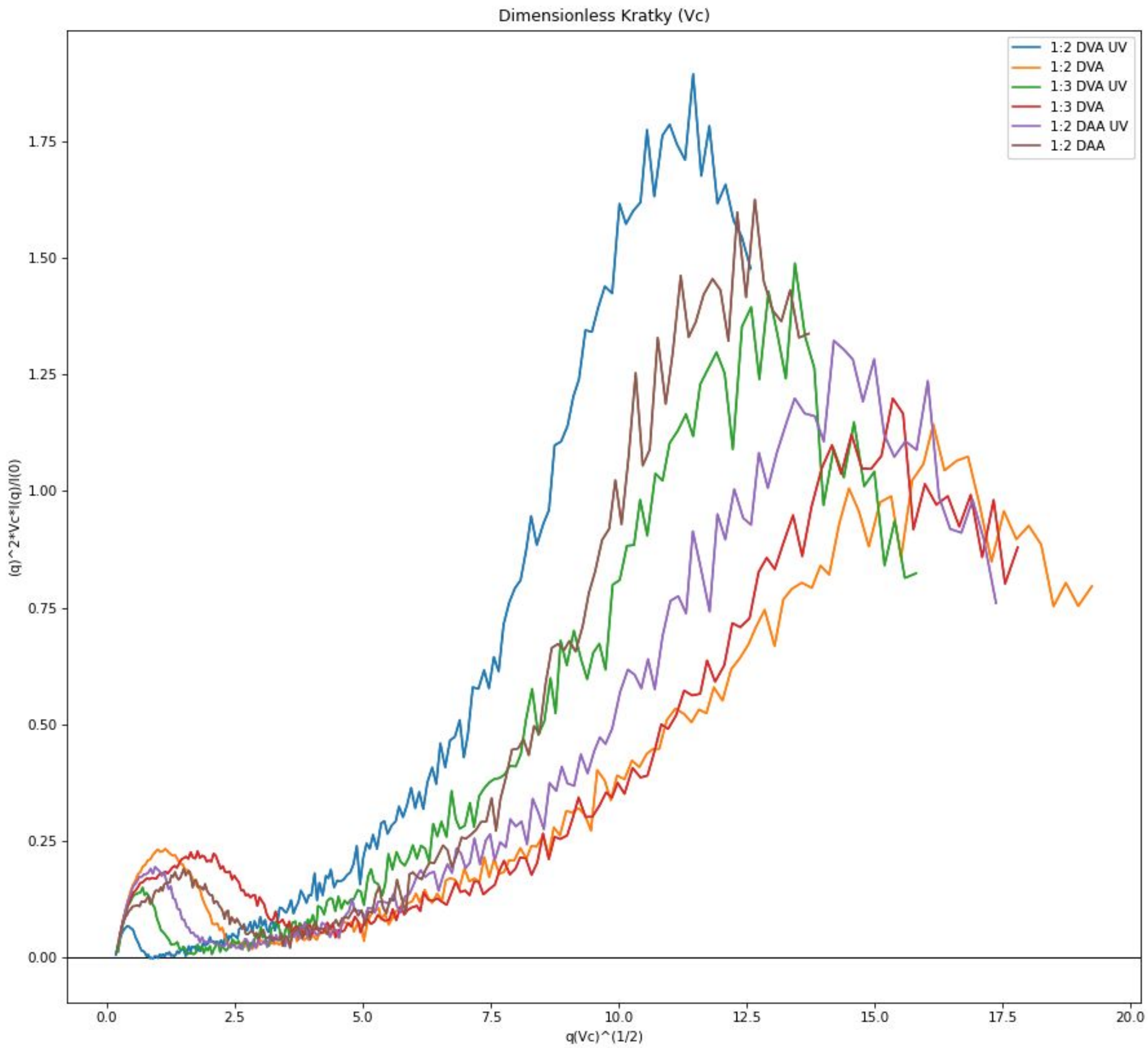

Figure S25. Normalized Kratky analysis versus qRg ($Å^{-1}$), which normalizes to the Vc (correlation volume) values.

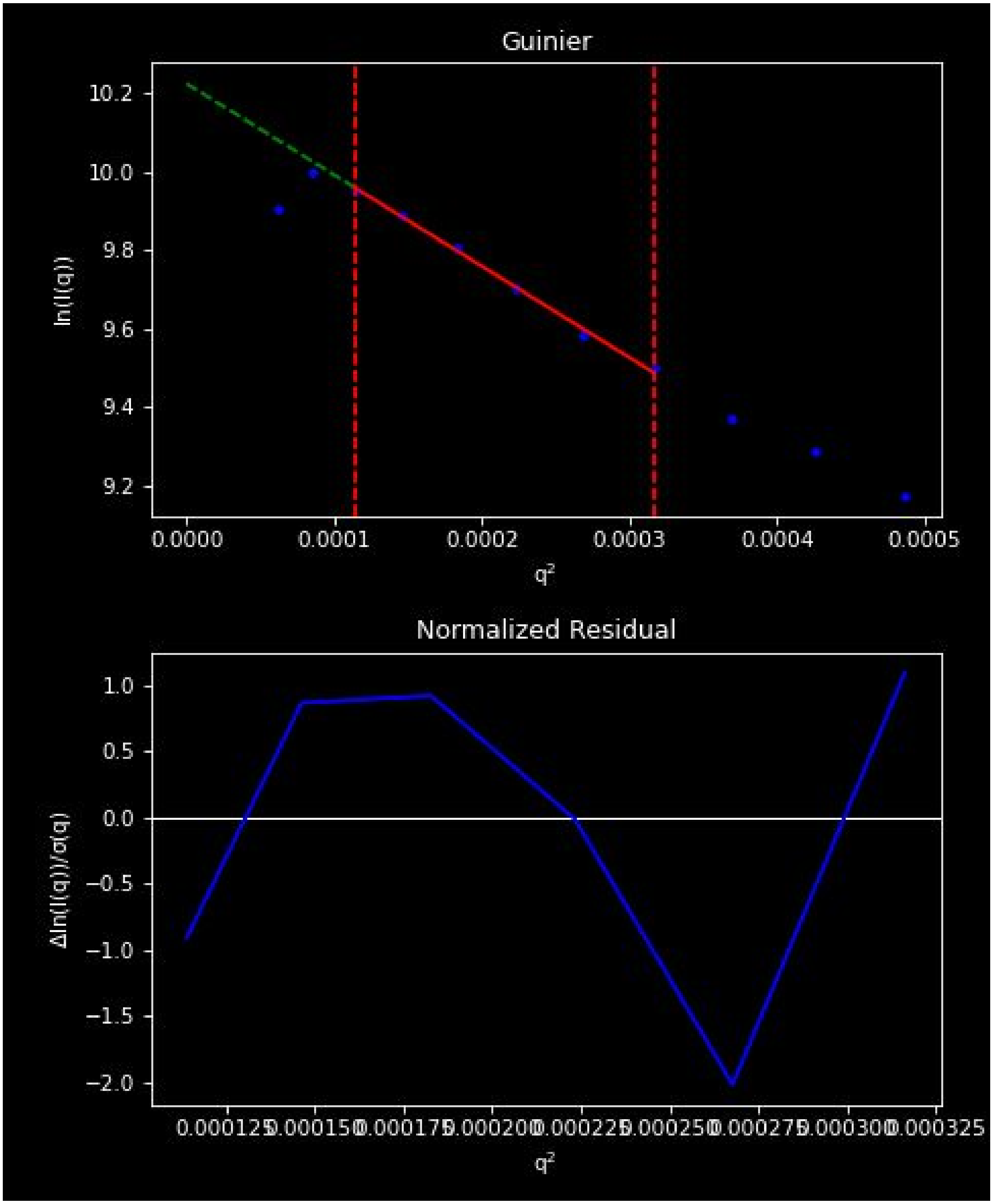

Figure S26. Guinier analysis of 1:2 DVA.

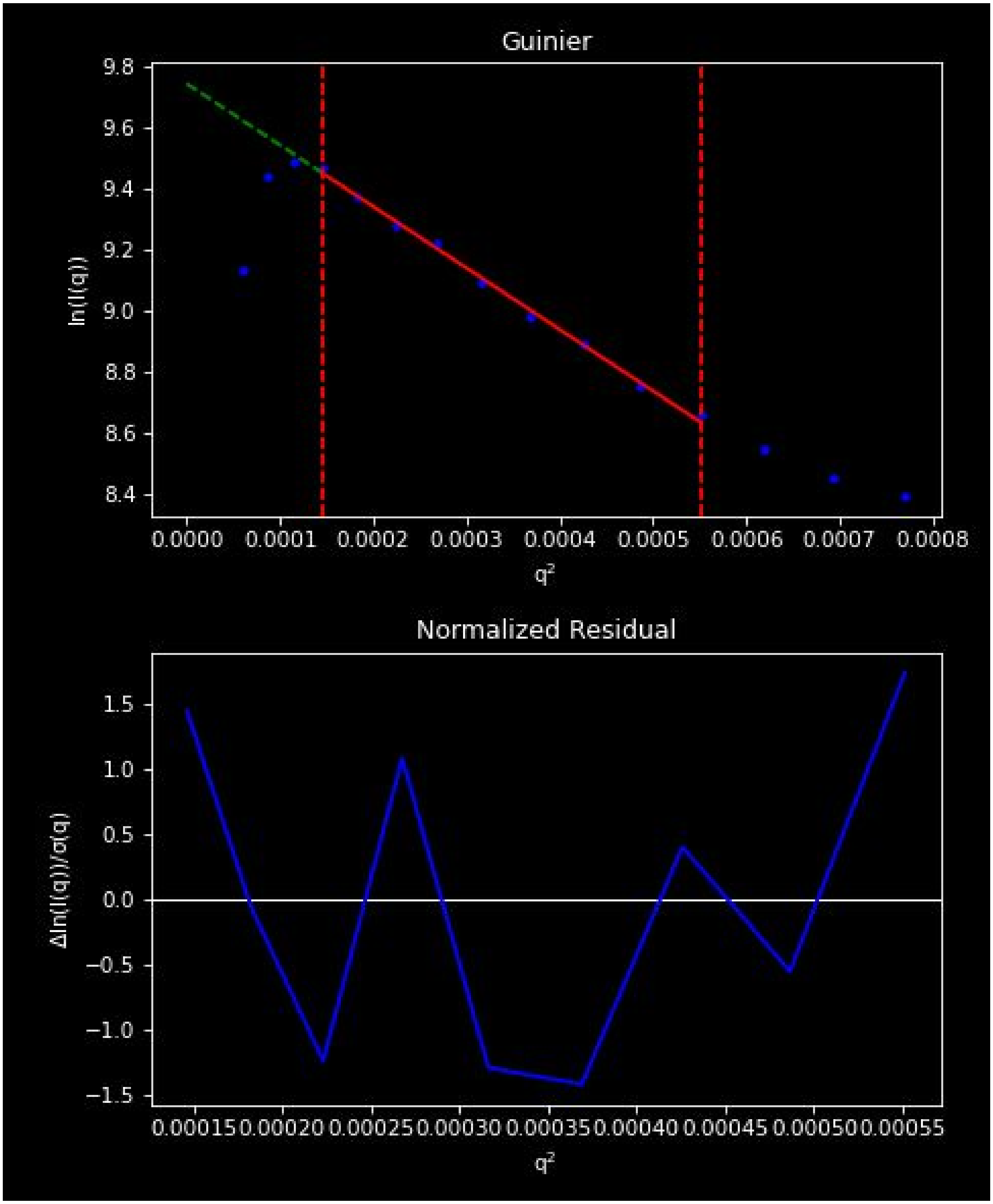

Figure S27. Guinier analysis of 1:2 DVA UV irradiated.

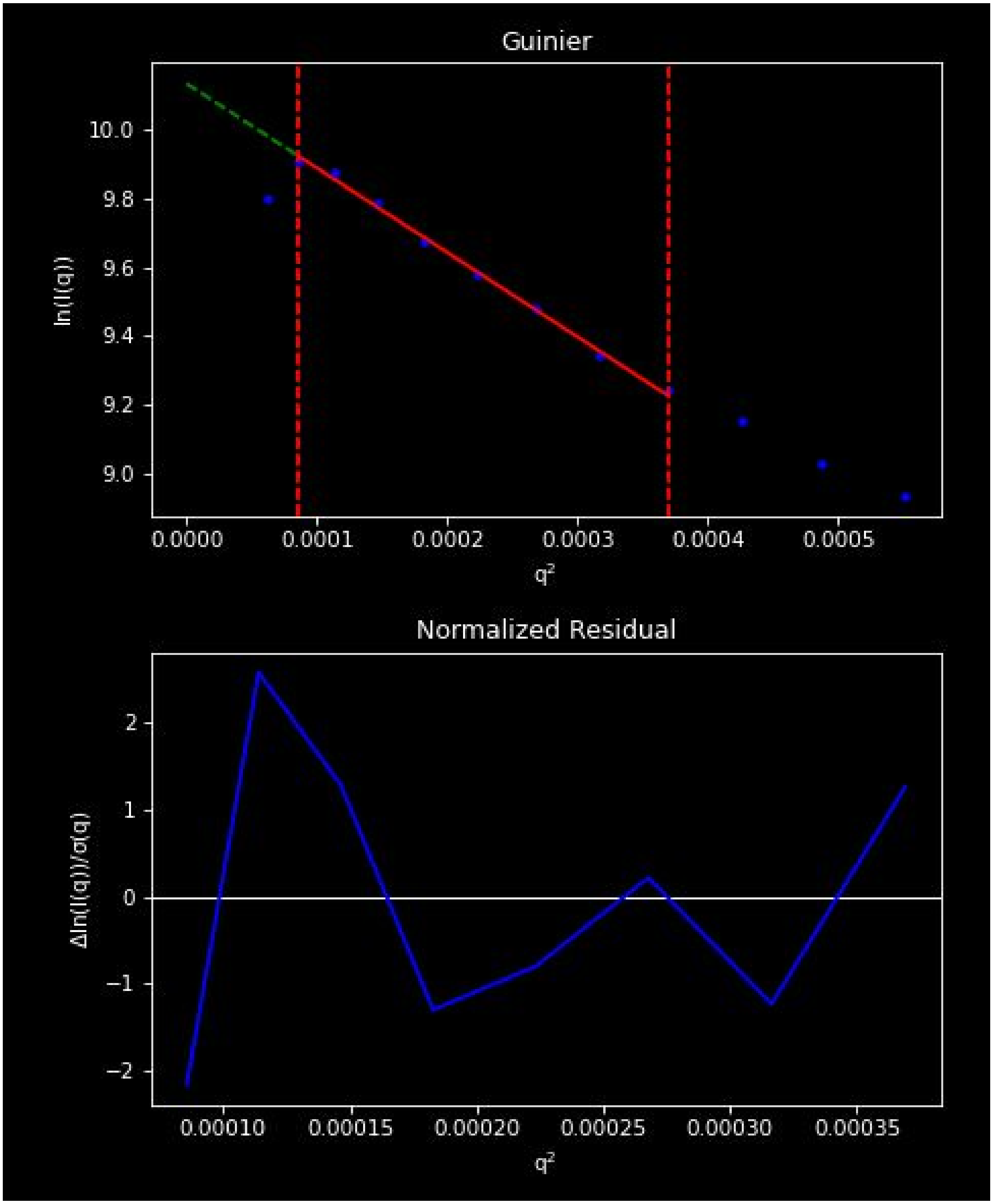

Figure S28. Guinier analysis of 1:3 DVA.

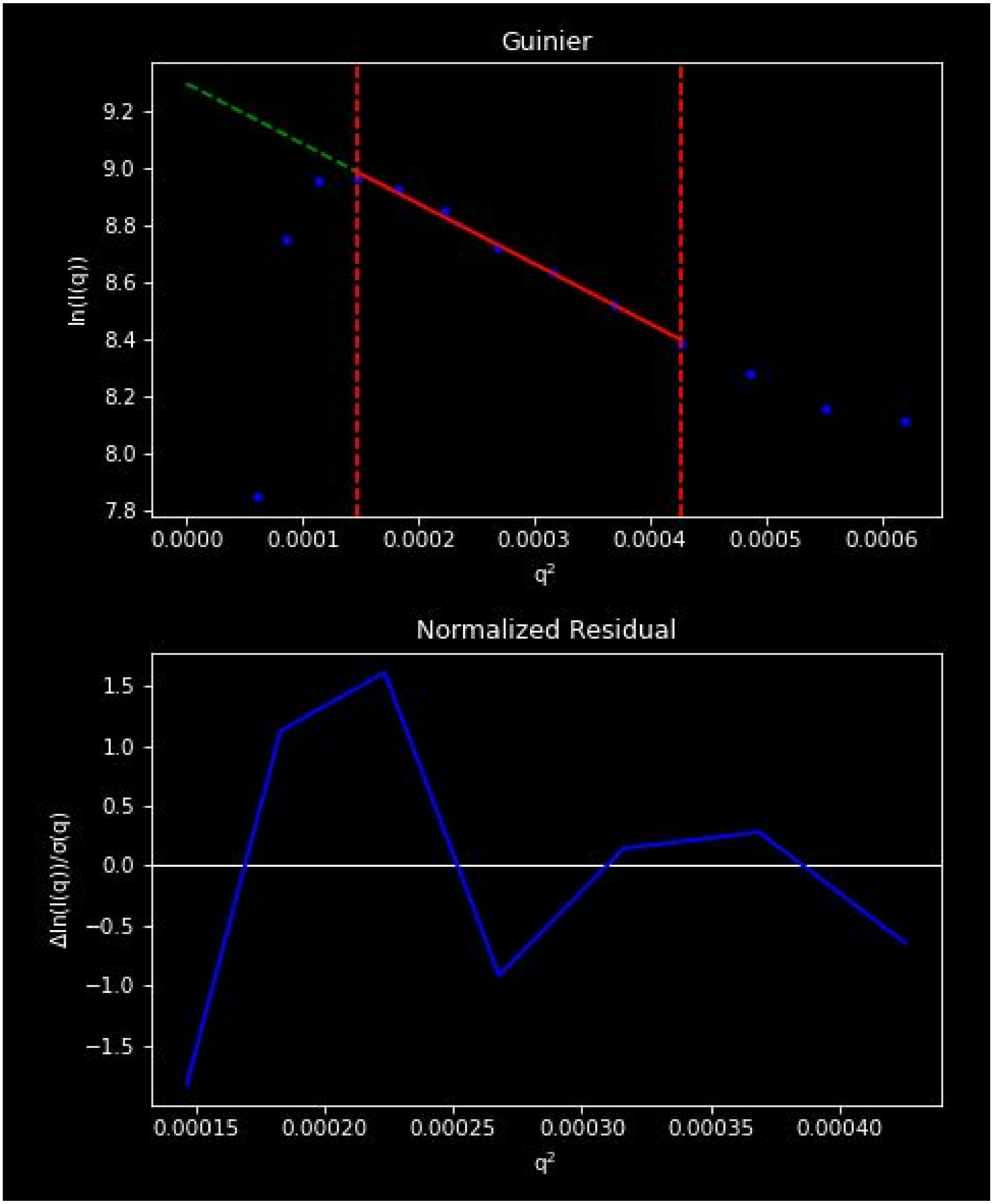

Figure S29. Guinier analysis of 1:3 DVA UV irradiated.

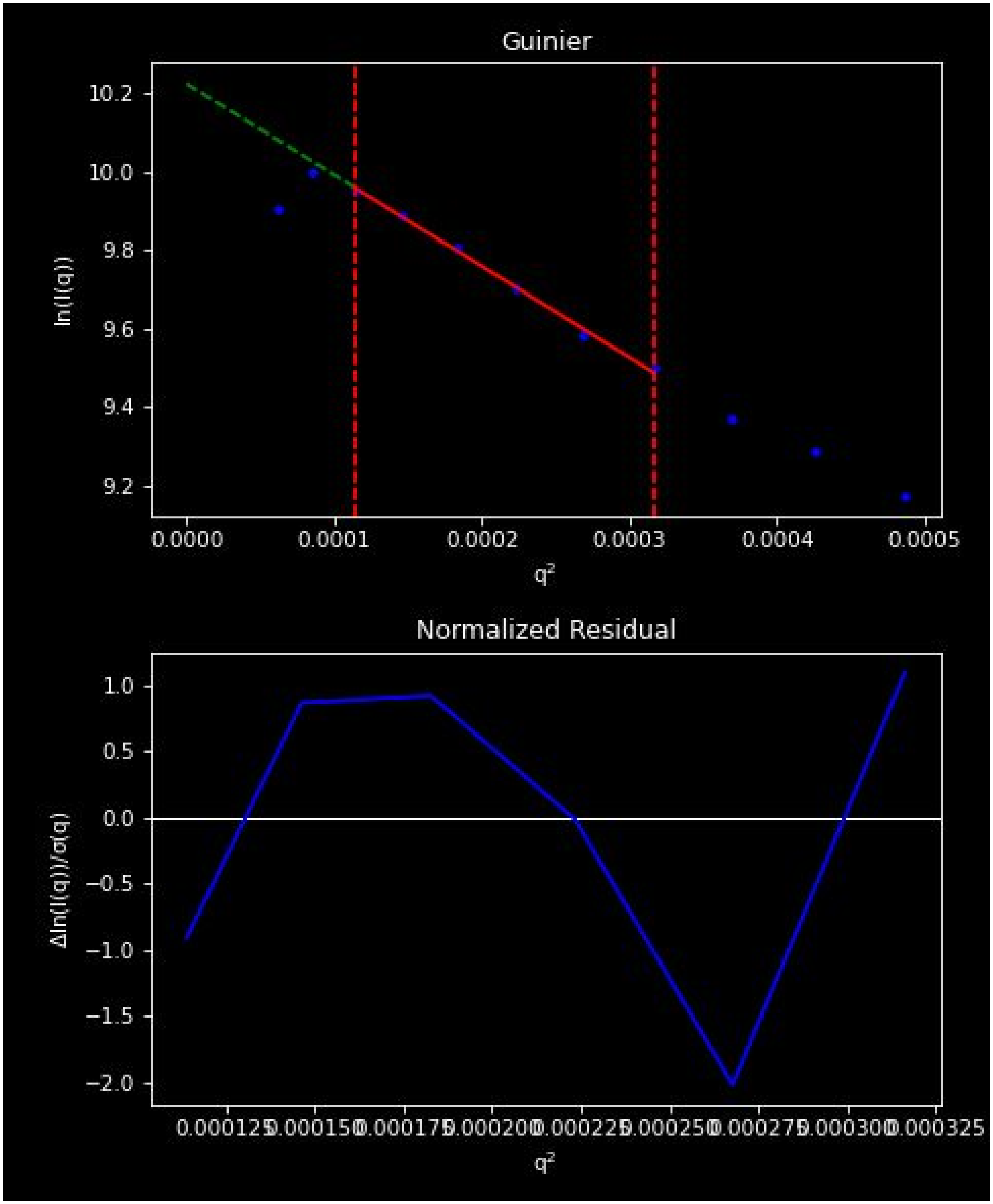

Figure S30. Guinier analysis of 1:2 DAA.

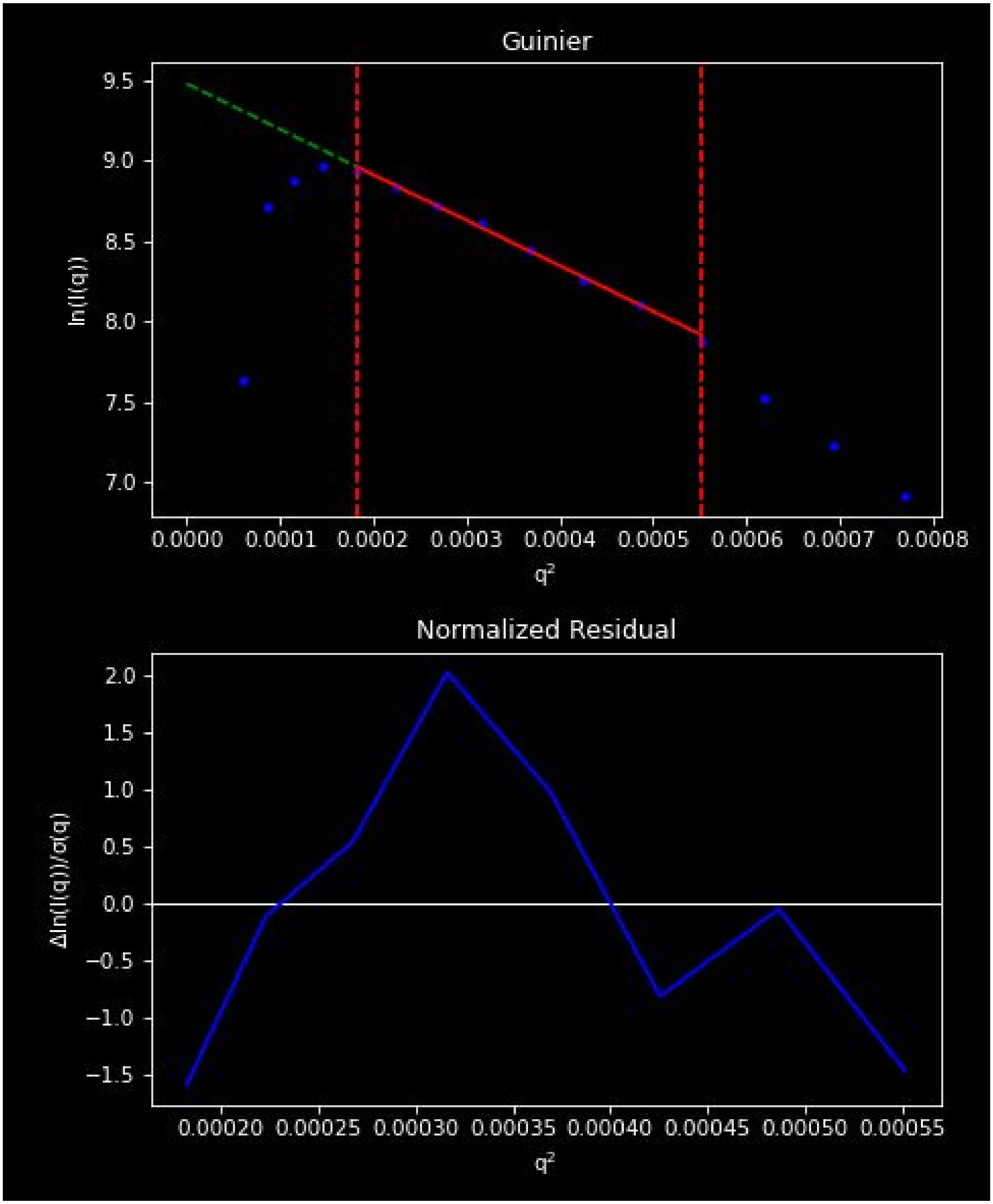

Figure S31. Guinier analysis of 1:2 DAA UV irradiated.

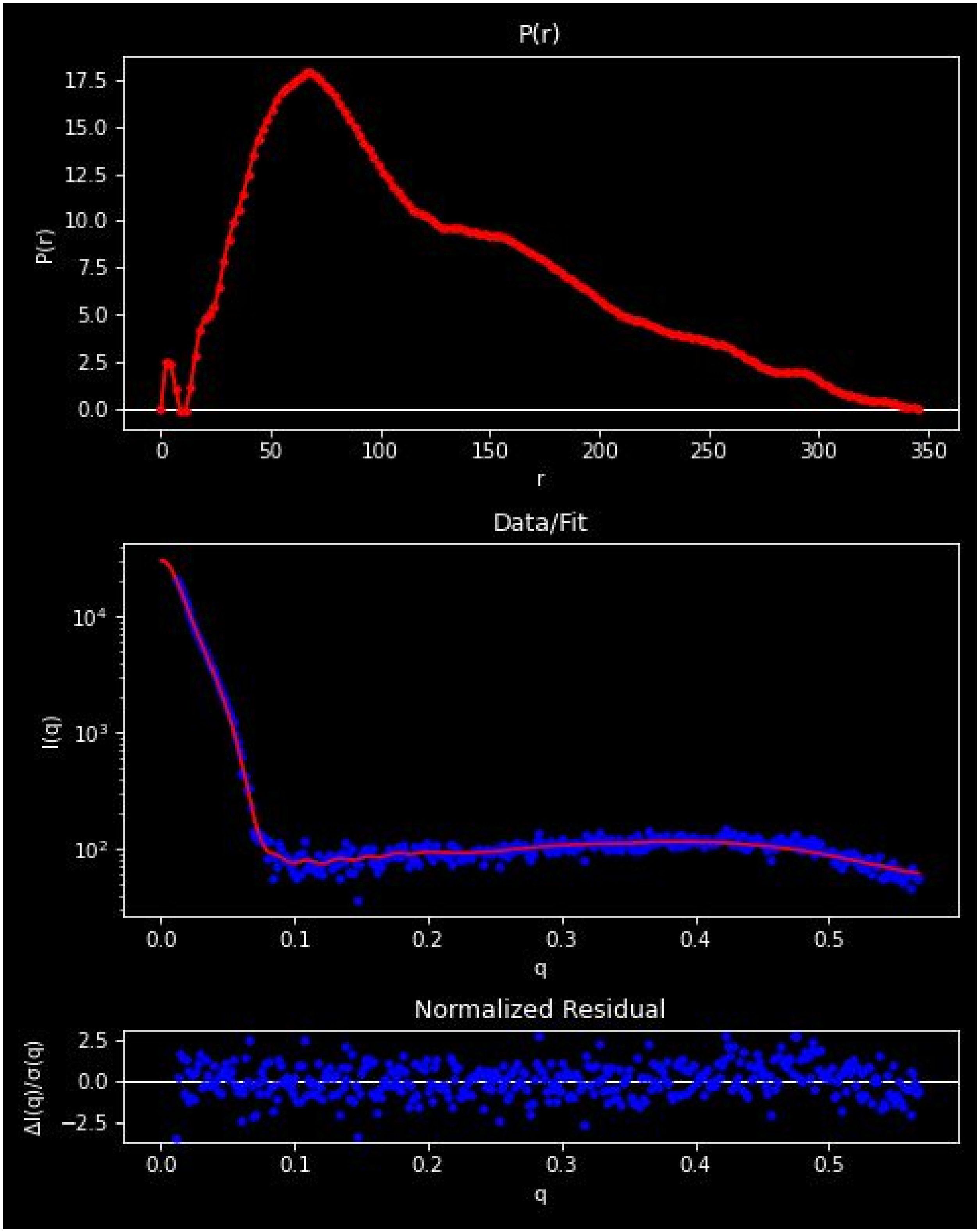

Figure S32. GNOM analysis of 1:2 DVA.

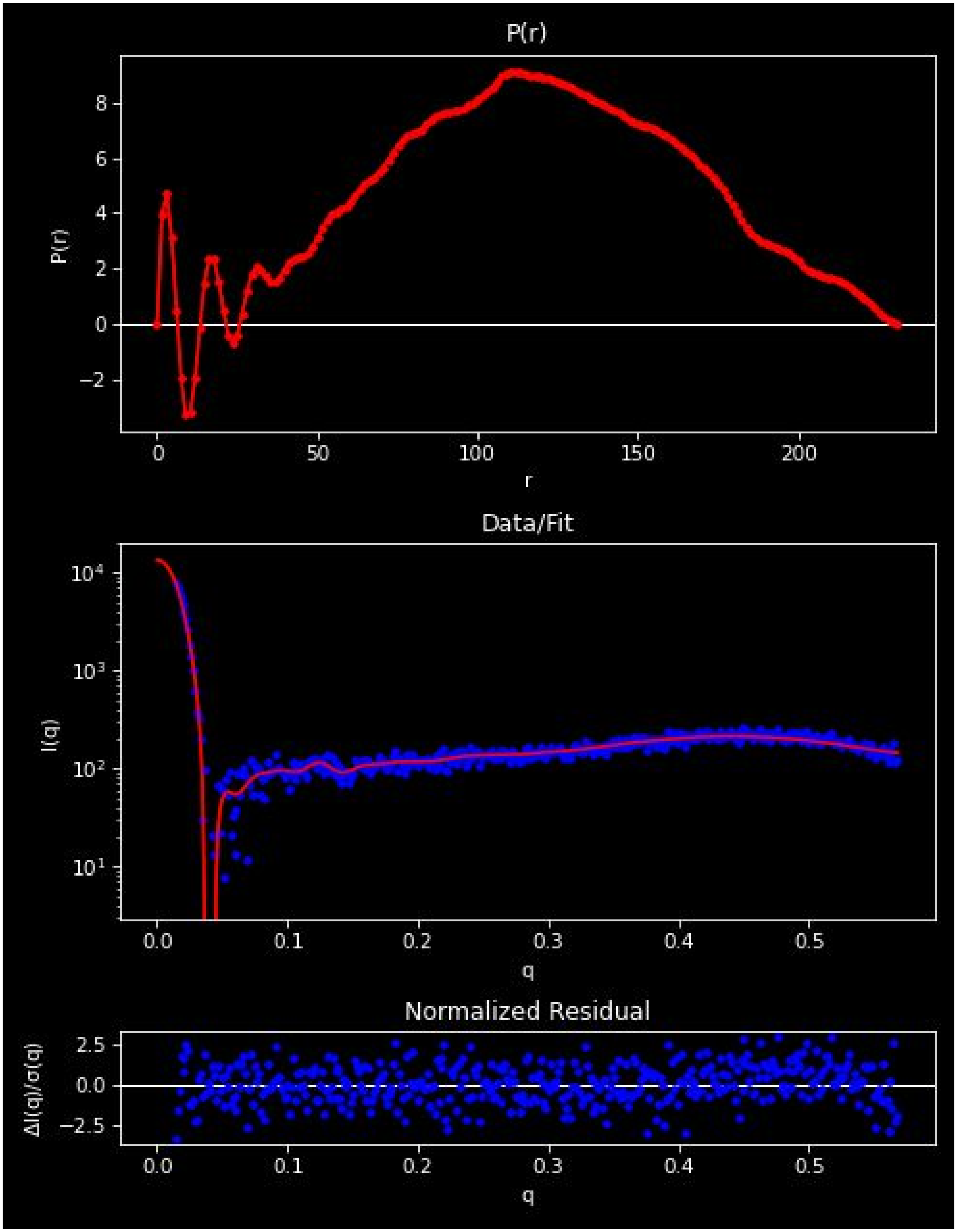

Figure S33. GNOM analysis of 1:2 DVA UV-irradiated.

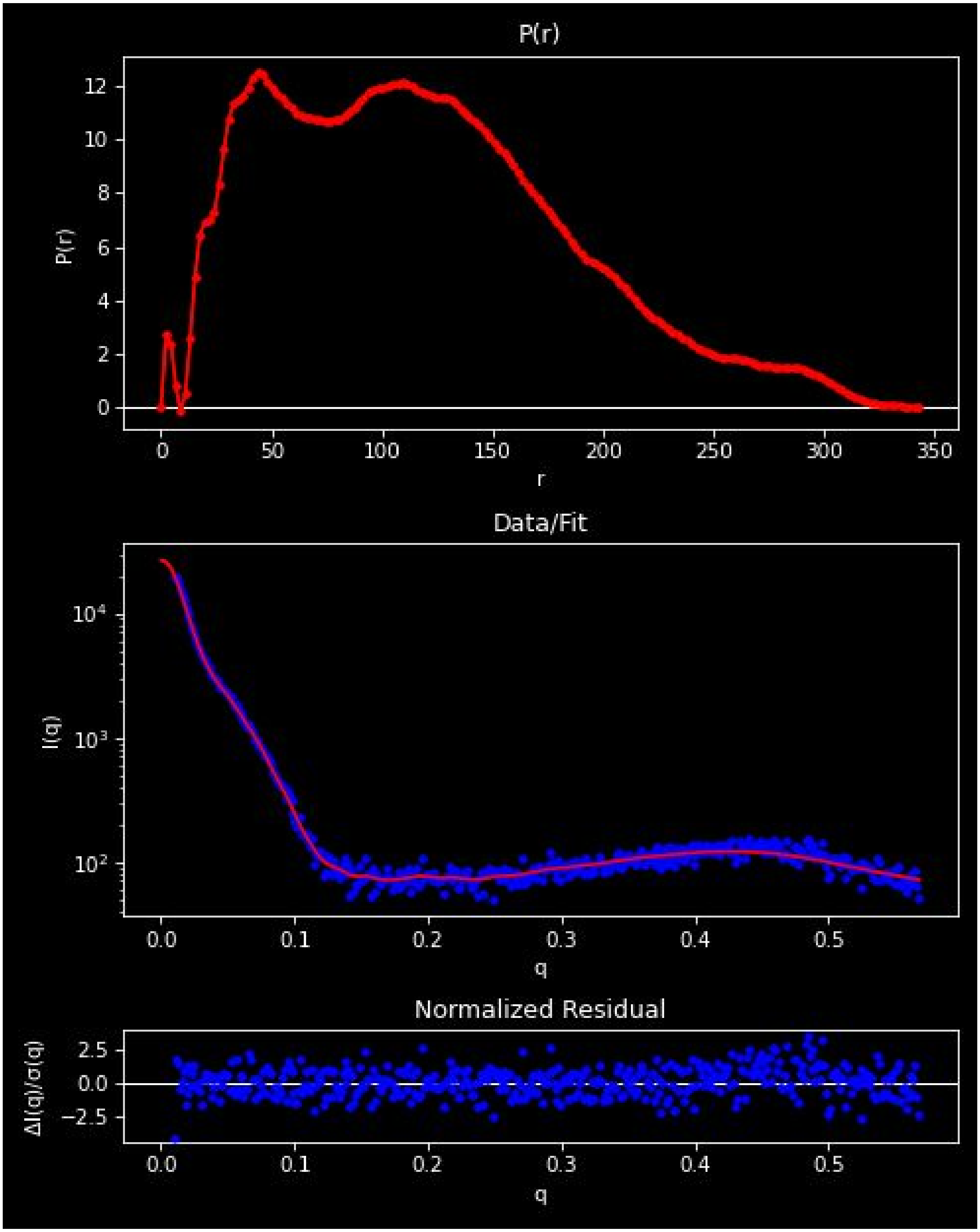

Figure S34. GNOM analysis of 1:3 DVA.

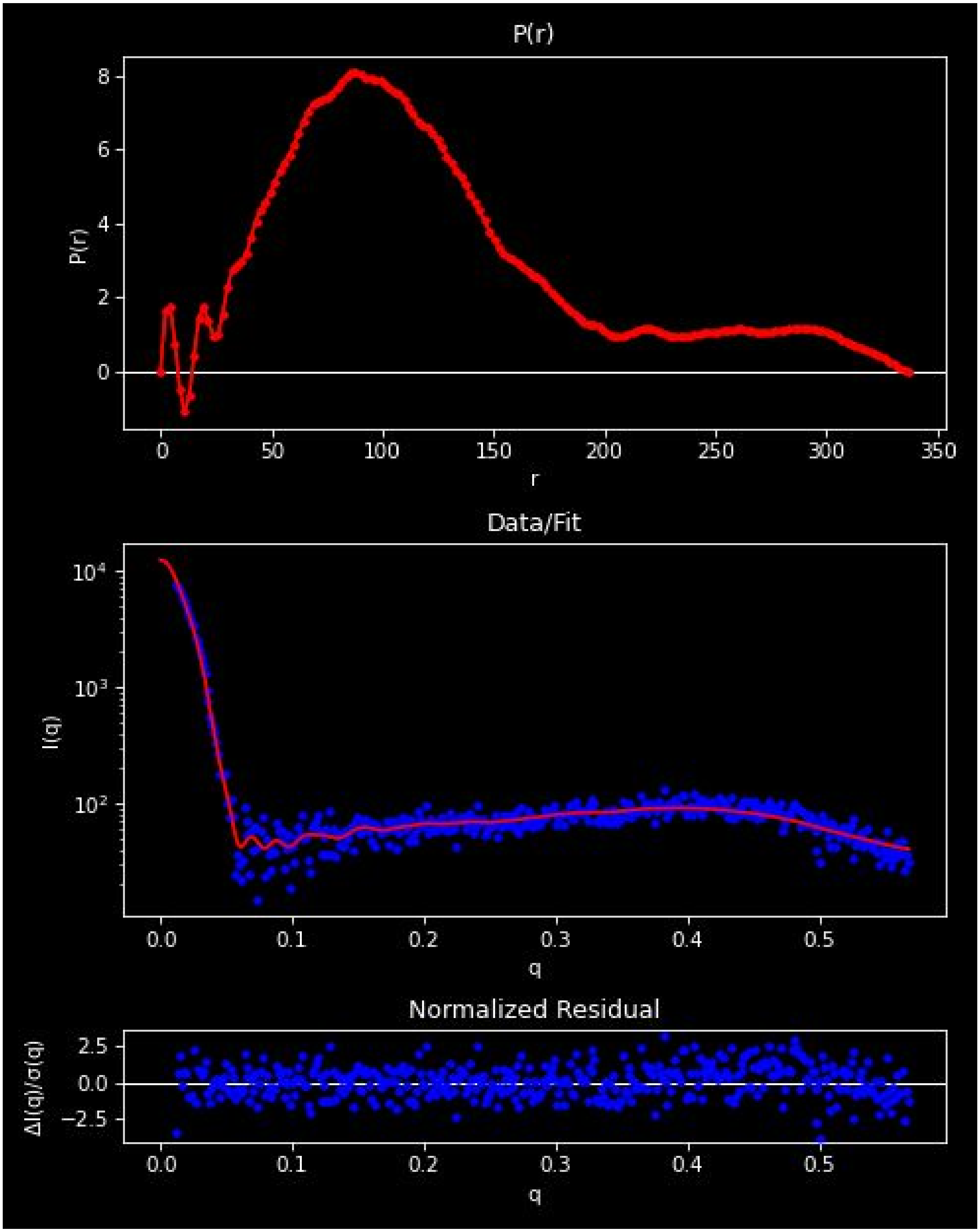

Figure S35. GNOM analysis of 1:3 DVA UV-irradiated.

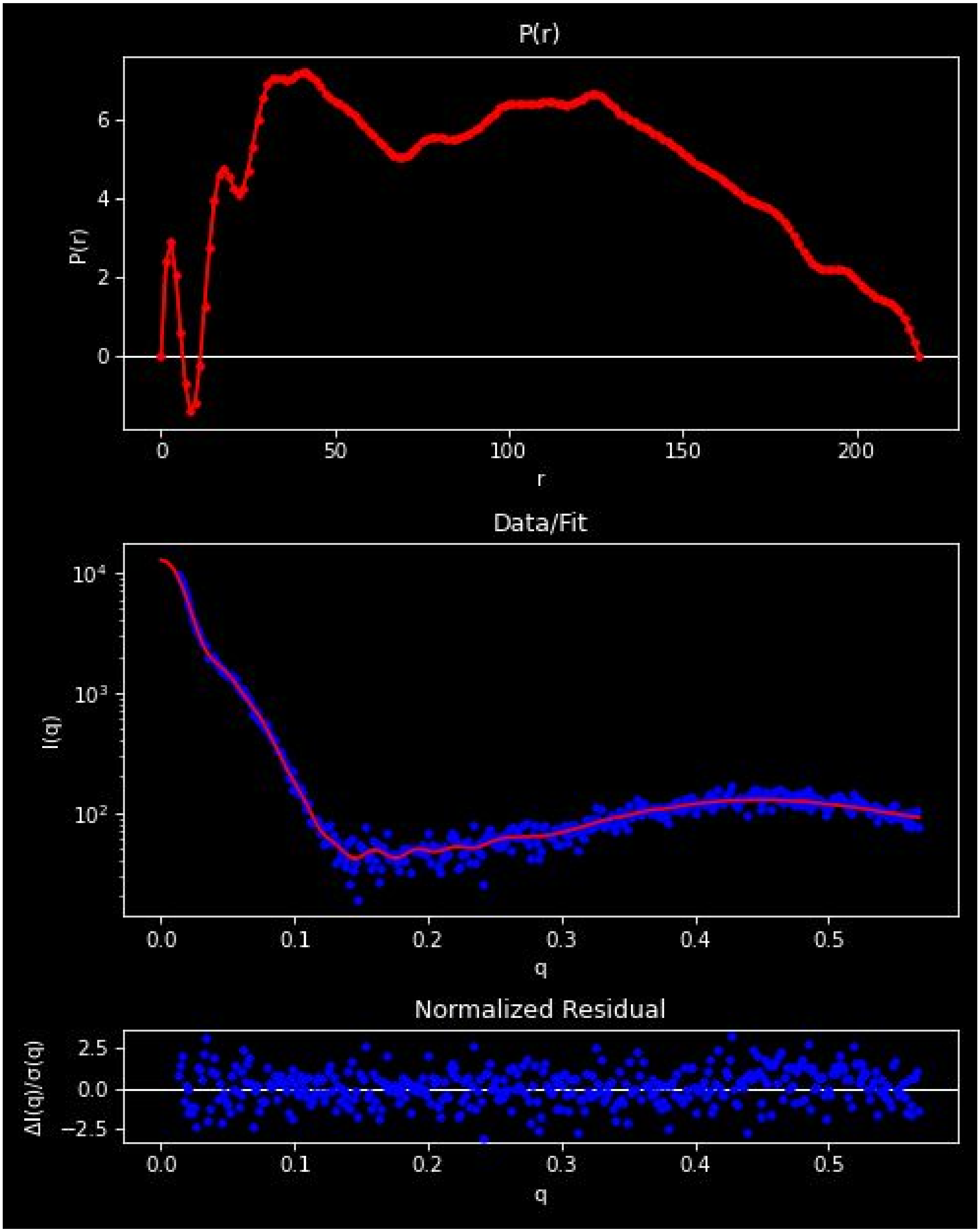

Figure S36. GNOM analysis of 1:2 DAA.

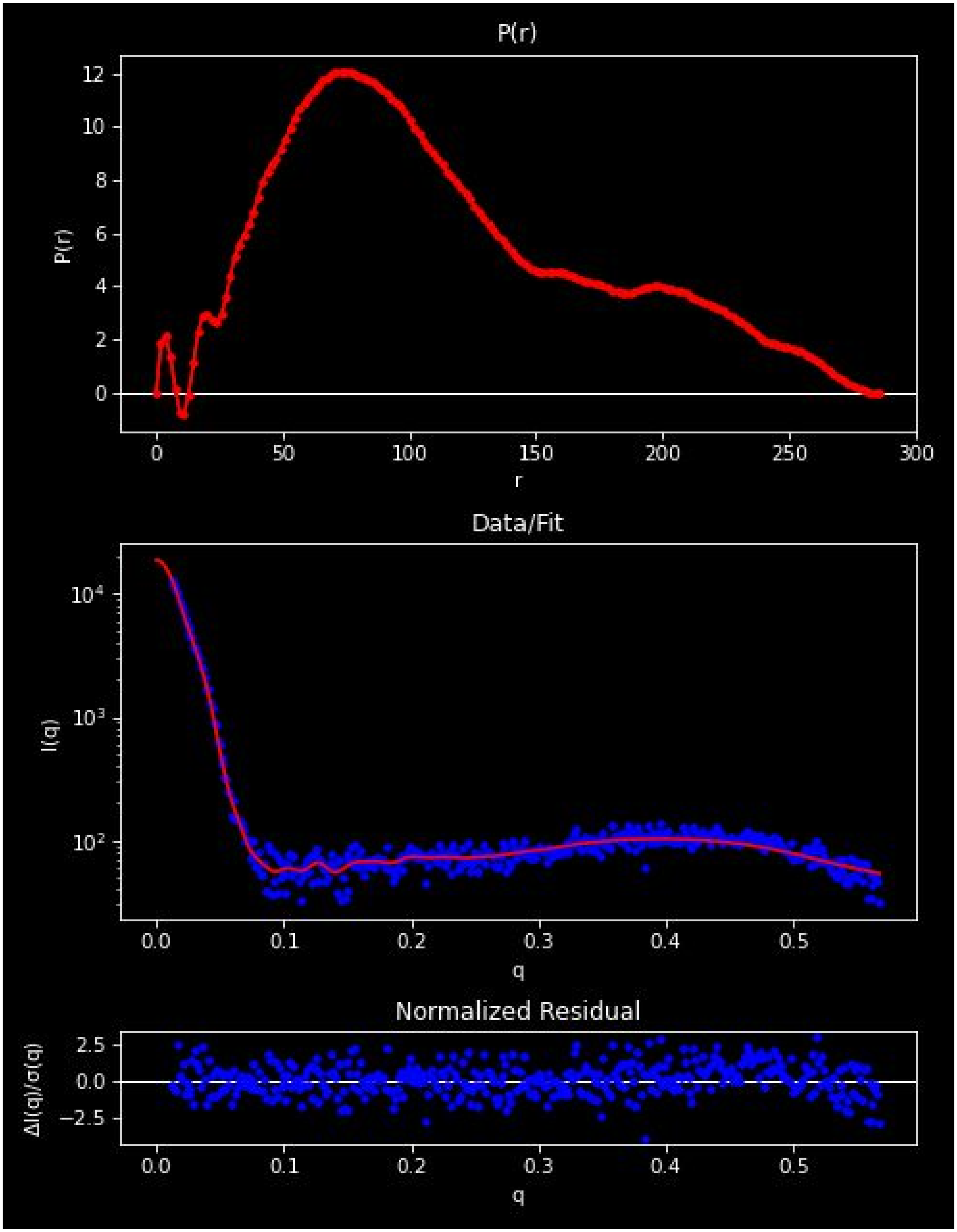

Figure S37. GNOM analysis of 1:2 DAA UV-irradiated.

Reichardt's Dye Polarity Studies: Reichardt's dye was used to investigate internal polarity changes during azobenzene isomerization within the material. Di-vinylazobenzene gel systems were studied by first casting DCM gels as bars in a 2 mm thick x 4 cm x 1 cm Teflon mold. A solution of Reichardt's dye at a 2 mg/mL concentration was then added to the gel in 8 regions using a syringe (0.5 mL total) under low-UV light conditions and allowed to disperse. This resulted in a blue/green colored gel. The gel was covered and left in a DCM vapor-rich environment to limit evaporation. After ~5 min, the dye became transparent, and the orange gel color resurfaced. UV/Vis of the actuation study of the gel are shown in Figures S11 and S12 below. No evidence of dye absorption is observable. Two control experiments were conducted to investigate the reason for dye degradation. The first was a Q-cage solution with the dye, which showed no change in dye color. The second was a divinylazobenzene solution containing the dye, which showed that the dye color was immediately removed upon dripping into the solution. This verified that the azobenzene had a negative influence on the dye structure, causing it to bleach/degrade rapidly.

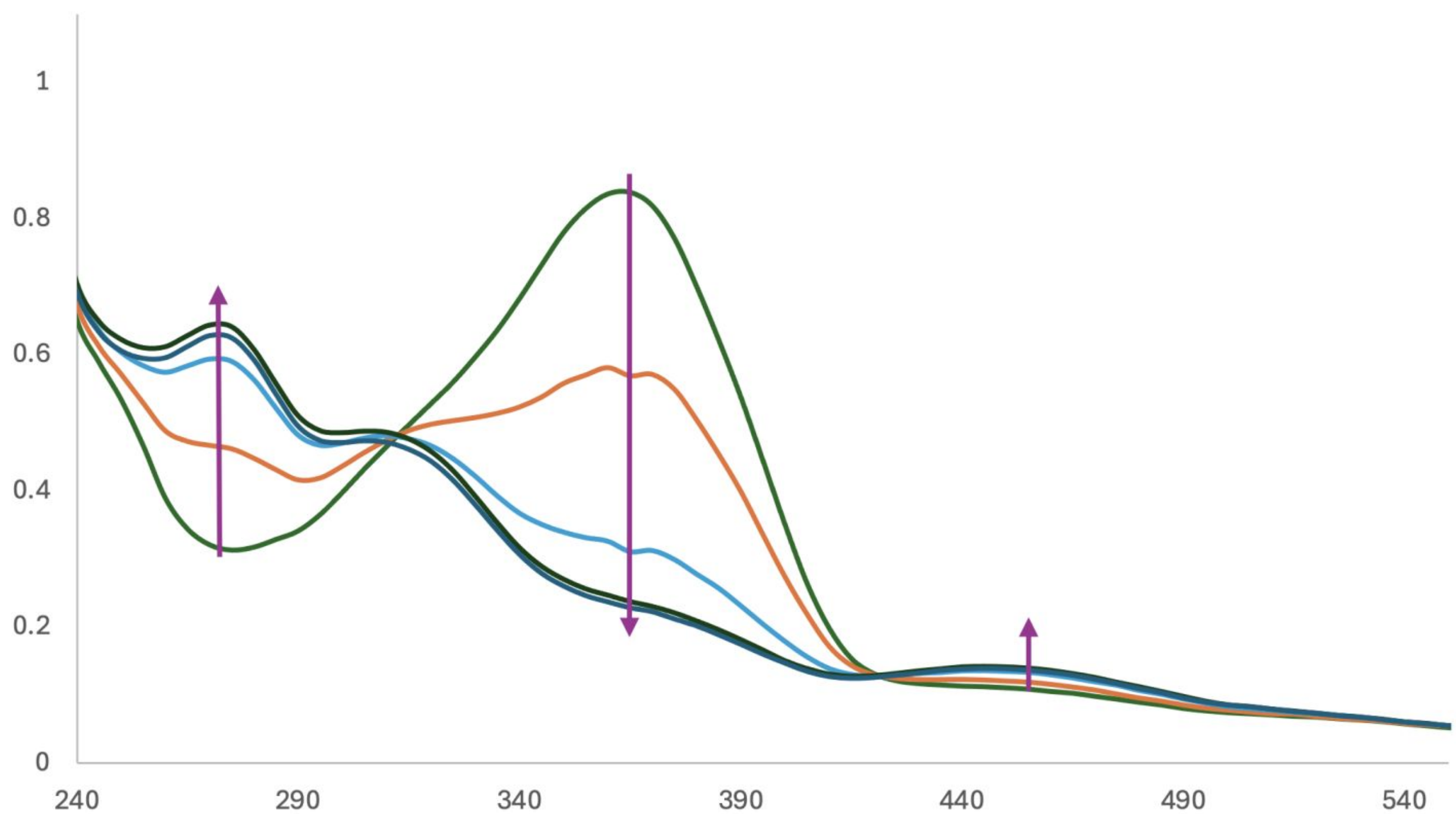

Figure S38. UV/Vis of 1:2 DVA gel under UV irradiation over a 10 min time period (in nm) in the presence of Reichardt's dye.

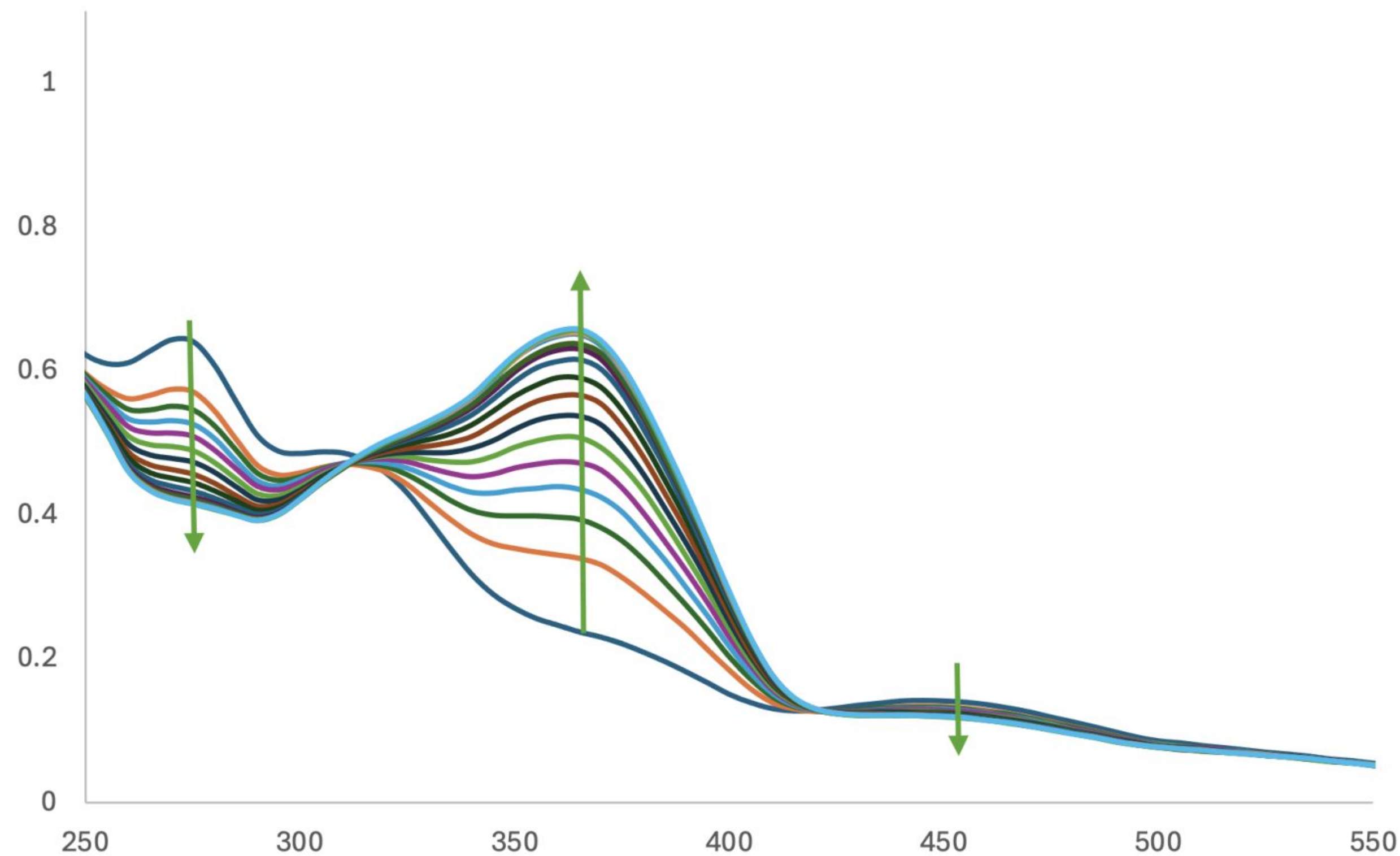

Figure S39. UV/Vis of 1:2 DVA gel under visible irradiation over a 10 min time period (in nm) in the presence of Reichardt's dye.

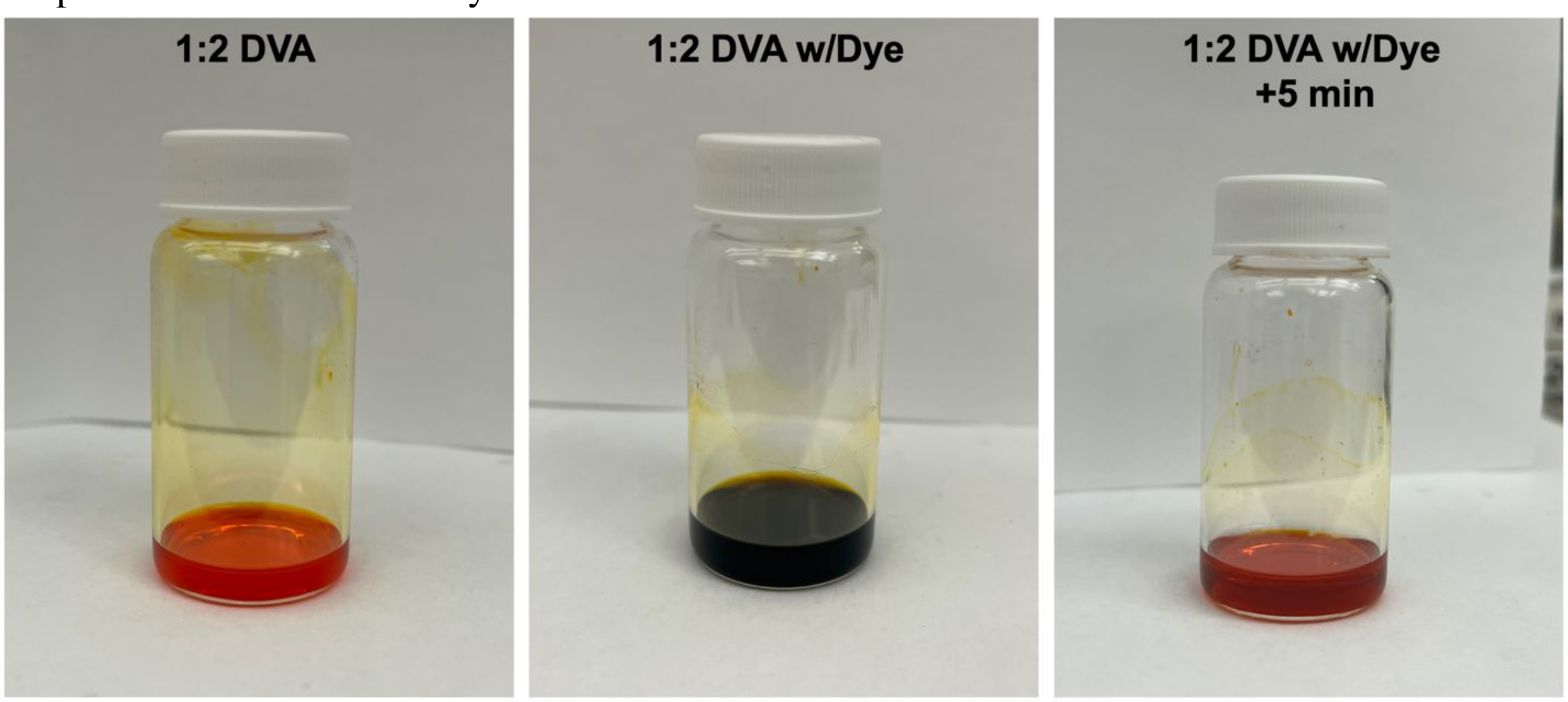

Figure S40. 1:2 DVA gel before and after Reichardt's dye showing dye decomposition within 5 min, even under ambient conditions.

Water Contact Angle Surface Polarity Studies:

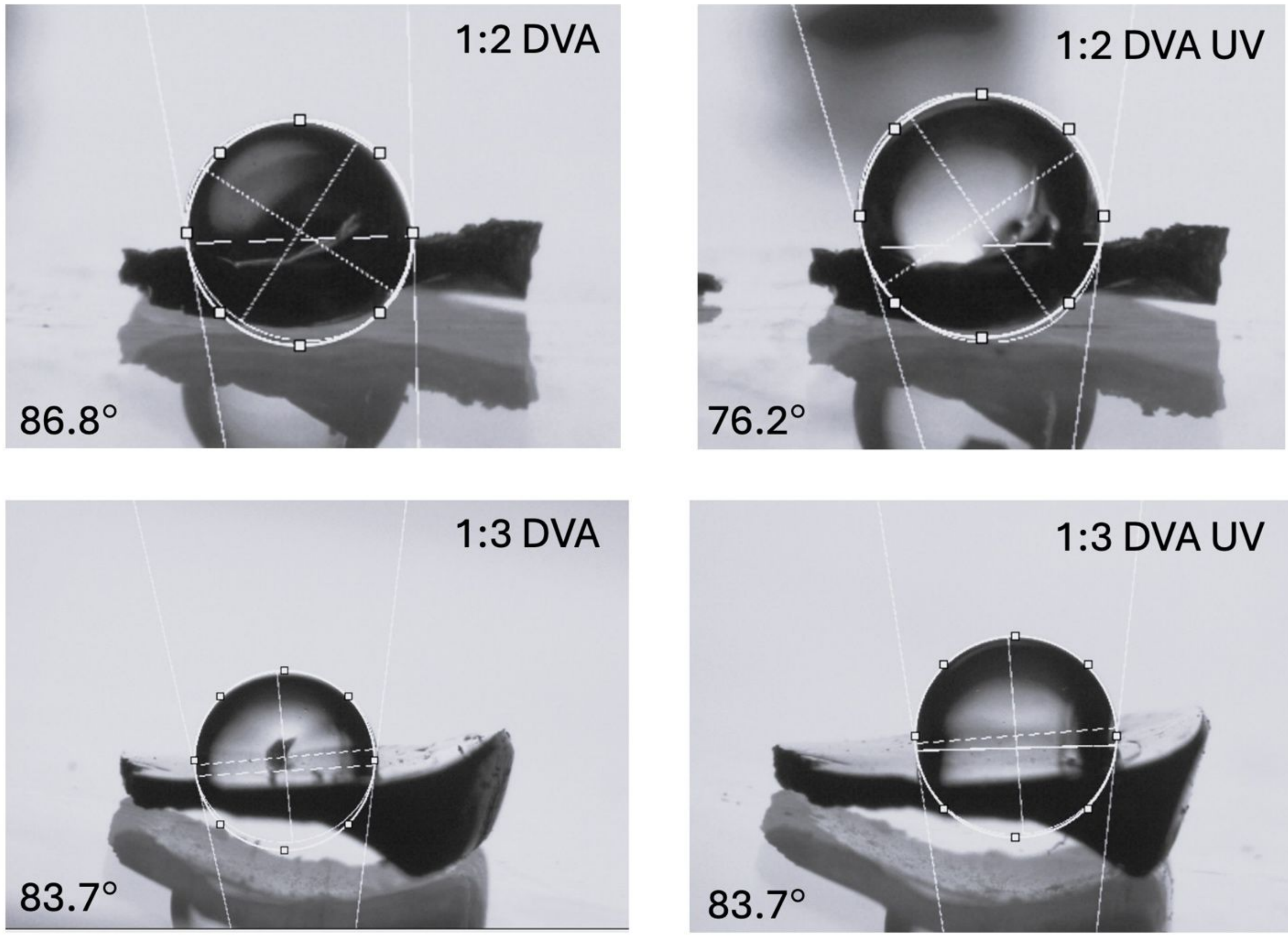

Figure S41. 1:2 DVA and 1:3 DVA water contact angle study.
